# Proteomics- and metabolomics-based analysis of the regulation of germination in Norway maple and sycamore embryonic axes

**DOI:** 10.1093/treephys/tpaf003

**Published:** 2025-01-06

**Authors:** Ewa Marzena Kalemba, Sara Dufour, Kris Gevaert, Francis Impens, Patrice Meimoun

**Affiliations:** Institute of Dendrology Polish Academy of Sciences, Parkowa 5, Kórnik 62-035, Poland; VIB-UGent Center for Medical Biotechnology, VIB, Technologiepark-Zwijnaarde 75, Ghent B-9052, Belgium; Department of Biomolecular Medicine, Ghent University, Technologiepark-Zwijnaarde 75, Ghent B-9052, Belgium; VIB Proteomics Core, VIB, Technologiepark-Zwijnaarde 75, Ghent B-9052, Belgium; VIB-UGent Center for Medical Biotechnology, VIB, Technologiepark-Zwijnaarde 75, Ghent B-9052, Belgium; Department of Biomolecular Medicine, Ghent University, Technologiepark-Zwijnaarde 75, Ghent B-9052, Belgium; VIB-UGent Center for Medical Biotechnology, VIB, Technologiepark-Zwijnaarde 75, Ghent B-9052, Belgium; Department of Biomolecular Medicine, Ghent University, Technologiepark-Zwijnaarde 75, Ghent B-9052, Belgium; VIB Proteomics Core, VIB, Technologiepark-Zwijnaarde 75, Ghent B-9052, Belgium; Laboratoire de Biologie du Développement, UMR 7622, Institut de Biologie Paris-Seine (IBPS), Sorbonne Université, CNRS, F-75005 Paris, France; Laboratoire Interdisciplinaire des Énergies de Demain (LIED UMR 8236), Université Paris-Cité, Paris, France

**Keywords:** *Acer platanoides*, *Acer pseudoplatanus*, metabolite profiling, methionine sulfoxide, posttranslational modification, redox status, seed physiology, vitamin B

## Abstract

Norway maple and sycamore belong to the *Acer* genus and produce desiccation-tolerant and desiccation-sensitive seeds, respectively. We investigated the seed germination process at the imbibed and germinated stages using metabolomic and proteomic approaches to determine why sycamore seeds germinate earlier and are more successful at establishing seedlings than Norway maple seeds under controlled conditions. Embryonic axes and embryonic axes with protruded radicles were analyzed at the imbibed and germinated stages, respectively. Among the 212 identified metabolites, 44 and 67 differentially abundant metabolites were found at the imbibed and germinated stages, respectively, in both *Acer* species. Higher levels of amines, growth and defense stimulants, including B vitamins, were found in sycamore. We identified 611 and 447 proteins specific to the imbibed and germinated stages, respectively, in addition to groups of proteins expressed at different levels. Functional analysis of significantly regulated proteins revealed that proteins with catalytic and binding activity were enriched during germination, and proteins possibly implicated in nitrogen metabolism and metabolite interconversion enzymes were the predominant classes. Proteins associated with the control of plant growth regulation and seed defense were observed in both species at both germination stages. Sycamore proteins possibly involved in abscisic acid signal transduction pathway, stress tolerance and alleviation, ion binding and oxygenase activities appeared to accompany germination in sycamore. We identified peptides containing methionine (Met) oxidized to methionine sulfoxide (MetO), and functional analyses of proteins with significantly regulated MetO sites revealed that translation, plant growth and development and metabolism of nitrogen compounds were the main processes under Met/MetO redox control. We propose that higher levels of storage proteins and amines, together with higher levels of B vitamins, supported more efficient nitrogen utilization in sycamore, resulting in faster seedling growth. In conclusion, omic signatures identified in sycamore seem to predispose germinated sycamore seeds to better postgerminative growth.

## Introduction

Norway maple (*Acer platanoides* L.) and sycamore (*Acer pseudoplatanus* L.) are widespread tree species in Europe that grow in temperate mixed forests and mixed softwood deciduous forests, respectively (Caudullo and de Rigo 2016, Pasta et al. 2016). Norway maple and sycamore regularly produce seeds that differ in terms of desiccation tolerance. Norway maple seeds are desiccation and freezing tolerant (orthodox), whereas sycamore seeds are sensitive to desiccation and freezing and are classified as recalcitrant (Hong and Ellis 1990, Dickie et al. 1991, Walters 2015). Notably, Norway maple and sycamore seeds are characterized by a deep physiological dormancy, and completion of germination requires 12–20 and 8–15 weeks, respectively (Suszka et al. 1996).

The transition from a seed to a plant is a critical stage in a plant’s life cycle, implicating ecological success in the environment and economic profit from a human perspective. Seed germination is a complex process coordinated by the interplay among external factors (light, hydration, oxygen), physical forces (mechanical forces between seed tissues) endogenous factors, including hormones (abscisic acid (ABA), gibberellins (GA) and ethylene (ET)), repair of desiccation damage and molecular control through specific transcription factors, epigenetic remodeling of chromatin and small RNAs (Carrera-Castaño et al. 2020, El-Maarouf-Bouteau 2022, Waterworth et al. 2022). Moreover, germination sensu stricto starts with imbibition and ends with the emergence of the radicle (Bewley and Black 2013). Seed vigour is a quantitative trait determining overall seed performance, including seed germination and seedling growth (Basu and Groot 2023). Seedling emergence and establishment are particularly vulnerable stages in global warming scenarios (Badano and Oca 2022). Therefore, climate change is postulated to affect seed vigour (Reed et al. 2022). In the genus *Acer*, sugar maple (*Acer saccharum*) and red maple (*A. rubrum*) trees are in the spotlight because of global warming-derived difficulties (Ahmed et al. 2023). However, Norway maple and sycamore species remain less investigated with regard to other *Acer* species. In this work, this gap is addressed by revealing regulators of seed germination metabolism in the two species at the imbibed stage as germination-promoting processes and at the germinated (protruded radicles) stage as seedling development-promoting processes.

Omics studies of tree non-model species with unsequenced genomes are challenging but necessary for environmentally and economically important tree species (Staszak and Pawłowski 2012). Seed proteomics studies provide data describing protein expression changes controlling seed development and maturation, germination and seedling performance (Narula et al. 2016). Functional analysis of differentially abundant proteins contributes to the explanation of molecular networks and pathways governing each seed developmental stage (Wang et al. 2015). Proteomic analysis of seed germination has been performed in many species, predominantly including model plants such as Arabidopsis (Gallardo et al. 2002), rice (He and Yang 2013) and barley (Osama et al. 2021), and has revealed the role of hormones, morphological changes and the activation of transcription, translation and metabolism in the regulation of this process. Proteomics research in forest trees, however, remains underrepresented (Castillejo et al. 2023), as well as genome-wide association studies and metagenomic studies (Naidoo et al. 2019). Gel-based proteomics of *Acer* seeds has been performed to analyze dormancy (Pawłowski 2009, Staszak and Pawłowski 2014, Pawłowski and Staszak 2016). In this study, we aim to provide a proteomic interpretation of the germination process at the imbibed and germinated stages in *Acer* species.

Among the different posttranslational modifications (PTMs), the widely studied protein phosphorylation and protein ubiquitination events are assumed to be involved in the regulation of seed germination, with a minor role for protein sumoylation, carbonylation, glycosylation, acetylation and succinylation (Yu et al. 2021). Other PTMs might also be involved in regulating seed germination; however, the shortage of experimental and computational methods has limited the discovery of many such PTMs (Ramazi and Zahiri 2021). Indeed, the integrative database of quantitative post-translational modification in plants (qPTMplants), for instance, contains data on phosphorylated, acetylated and ubiquitinated proteins detected in adult trees but not seeds (Xue et al. 2022). Oxidative PTMs are less studied, with studies to date focused on protein carbonylation in germinating seeds (Job et al. 2005, Zhang et al. 2016, Boucelha et al. 2021). Reactive oxygen species (ROS)-mediated oxidation of proteins contributes to the redox state of germinating tissue (Chakrabarty et al. 2019). Information related to the role of the oxidation of methionine (Met) to methionine sulfoxide (MetO) in seed germination is not currently available; however, its role in seed longevity was recently demonstrated (Kalemba et al. 2024). Met residues in proteins act in antioxidant defense, redox sensing and regulation, as well as in protein structure and activity modulation (Kim et al. 2014). Proteins containing MetO are considered structural antioxidants, with such Mets exposed at protein surfaces to capture ROS, thereby preventing oxidative damage to other residues that are more critical to protein function (Schindeldecker and Moosmann 2015). In addition to studies of protein-bound Met oxidation in 5-week-old *Arabidopsis* plants subjected to oxidative stress conditions (Jacques et al. 2015), global MetO studies on proteins have been performed in nonplant systems. Even less information is available for seeds, as only unstressed mature Arabidopsis seeds (Kalemba et al. 2022) and aged beech seeds have been analyzed (Kalemba et al. 2024). Redox cycling of Met residues in proteins during *Acer* seed germination requires further study, as seeds seem to differ greatly in protein-bound MetO levels at the imbibed and germinated stages (Wojciechowska et al. 2020*b*).

Forest tree metabolomics mainly involves species with economic and ecological significance, particularly the *Pinus*, *Quercus* and *Eucalyptus* genera (Rodrigues et al. 2021). Such comprehensive and high-throughput analysis of complex metabolite mixtures has also been applied in seed studies in recent years, enabling the discovery of metabolite biomarkers. Galactose and gluconic acid act as metabolomic biomarkers of seed vigour and ageing in rice (Chen et al. 2022). Seven key metabolites (N-acetyl-l-glutamic acid, l-ascorbate, lysine butyrate, *N-caffeoyl* putrescine, anthranilic acid, LysoPE 18:0 and inositol) promote the germination of rice seeds and are highly correlated with seedling performance (Yang et al. 2019). Free amino acids, members of the glutamic acid family, such as gamma-aminobutyric acid (GABA) and predominantly glutamine (Gln), shape metabolic homeostasis in Arabidopsis seeds and are implicated in seed vigour (Slaten et al. 2020). Among volatile metabolites, benzaldehyde, *n*-nonanal and 1-butanol are key metabolites identified as markers of seed vigour (Zhang et al. 2022). Except for *Eugenia* tropical seeds, the metabolic fingerprint of germinating seeds exhibiting contrasting desiccation tolerance in the dry state is less characterized (Rodrigues et al. 2024). B vitamins act as precursors of enzymatic cofactors modulate metabolic processes in seeds by increasing antioxidant activity and enhancing germination (Neumann et al. 1996, Xin Chi et al. 2021, Zeboon and Baqir 2023). Therefore, among metabolites, B vitamins got our special attention in this study.

Our previous results revealed that sycamore seeds germinated 3 weeks earlier than Norway maple seeds, and both exhibited high germination rates (Alipour et al. 2021). The biometric parameters of the seedlings established from these seeds differed, as sycamore seedlings exhibited greater root biomass (Alipour et al. 2023) and better seedling performance (~80% in sycamore compared with ~60% in Norway maple). In this study, we investigated Norway maple and sycamore embryonic axes at the imbibed and germinated stages. To map the mechanisms governing seed germination and determine the basis of more successful seedling performance under controlled conditions, we performed proteomic and metabolomic analyses comparing sycamore and Norway maple. Additionally, we investigated the oxidative PTM of methionine (MetO) in proteins to evaluate whether redox changes in selected proteins are involved in the modulation of the germination process and to showcase whether redox regulation differs at the imbibed and the germinated stages between *Acer* species.

## Materials and methods

### Seed samples

Seed samples consisted of embryonic axes isolated from Norway maple and sycamore seeds. Mature seeds were collected from individual trees growing in Kórnik (western Poland) and dehydrated to 7% (Norway maple) and 30% (sycamore) of water content (WC) expressed on a fresh weight basis. The average dry weight (DW) of one embryonic axis of mature *Acer* seeds used in our studies was similar in Norway maple and sycamore (2.15 ± 0.27 and 2.23 ± 0.12 mg, respectively). Dried, nonstored seeds were used. Respecting the definition of germination sensu stricto (first phase—imbibition, second phase—reactivation of metabolism, third phase—radicle protrusion) (Bewley and Black 2013) sampling occurred at the imbibed stage (completed phase 1) and the germinated stage (completed phase 3). For imbibition, dry seeds were hydrated for 24 h on wet paper towels. The WC of the imbibed embryonic axes was 39–44%. Imbibed seeds were placed between moist paper towels in separate plastic boxes and prepared for germination using cold stratification at 3 °C. Seeds were considered germinated when the radicle protruded by 5 mm across the seed testa and were collected at the ninth (sycamore) and 12th (Norway maple) weeks after imbibition. The WC of the embryonic axis with a protruded radicle at the germinated stage ranged from 57 to 58%. Sampling for the determination of the content of B vitamins and omics analyses occurred at the imbibed and germinated stage when 20 separated embryonic axes (imbibed stage) and 20 embryonic axes with protruded radicles (germinated stage) per sample were frozen in tubes at −80 °C. The same seedlots were used for the germination (Wojciechowska et al. 2020*b*, Alipour et al. 2021) and seedling establishment studies (Alipour et al. 2023). All experiments were designed to compare two *Acer* species, Norway maple and sycamore at the imbibed and the germinated stages using three biological replicates each time.

### Metabolomics

Samples (two *Acer* species × two germination stages × three biological repetitions) consisting of 200 mg of dry powder each were extracted with 75% methanol twice, using 0.5 mL of MeOH per 100 mg of sample. Next, the samples were centrifuged at 11,000×*g* for 5 min, and the obtained supernatants were transferred to new Eppendorf tubes. The samples were then dried in a vacuum centrifuge, and methoxyamine hydrochloride (20 mg/mL in dry pyridine) was added to each sample (50 μL/100 mg dw), followed by incubation in a thermomixer for 1.5 h at 37 °C. Then, after centrifugation, N-methyl-N-(trimethylsilyl)trifluoroacetamide (MSTFA) was added to each sample (80 μL/100 mg dw). The samples were incubated in a thermomixer for an additional 30 min at 37 °C and centrifuged at 11,000×*g* for 10 min. One hundred microliters of each sample was transferred to conical glass vials for GC–MS analysis. Metabolites were separated and analyzed using a GC–MS system (TRACE 1310 GC oven with TSQ8000 triple quad MS from Thermo Scientific, USA) comprising a DB-5MS column (30 m × 0.25 mm × 0.25 μm) (J & W Scientific, Agilent Technologies, Palo Alto, CA, USA). The conditions for the gradient during chromatographic separation were as follows: 70 °C for 2 min, then 10 °C/min to 300 °C, and hold at 300 °C for 10 min. Helium was used as the carrier gas. A PTV injector was used to inject the sample with a temperature gradient from 40 to 250 °C, the column interface was kept at 250 °C and the source temperature was set to 250 °C. The EI ion source was operated in the m/z range of 50–850, and the electron ionization energy was set at 70 eV. The retention index (RI) mixture containing alkanes was run prior to relevant analyses.

The raw data files were loaded into MSDial software (v. 4.90). To eliminate the shift in the retention time (Rt) and to determine the RI for each compound, a correction against an alkane series mixture (C-10 to C-36) was implemented directly in MS Dial. For compound identification, an MSP database from the CompMS site containing 28,220 records was used. The identified artefacts (alkanes, column bleeds, plasticizers, MSTFAs and reagents) were excluded from further analyses. The obtained normalized (using quality control (QC) samples and implementing the LOWESS algorithm) results were then exported to Excel for preformatting and then used for statistical analyses. The ion intensities were transferred into Perseus software v. 1.6.1.3 (Tyanova et al. 2016), transformed to log_2_ values and filtered for blanks in samples (min. 70% presence in each group). Missing values were imputed from a normal distribution, and such prepared matrices were used for statistical calculations. The differentially abundant metabolites (DAMs) (according to two-way analysis of variance) were plotted in a heatmap after nonsupervised hierarchical clustering using Z-scored LFQ intensities from significantly up- and downregulated metabolites and subjected to metabolic pathway enrichment analysis using the MetaboAnalyst v. 5.0 (https://www.metaboanalyst.ca/) (accessed on 15 November 2023) platform with default settings. Metabolite abundance between pairs of sample groups (sycamore vs Norway maple at the imbibed and the germinated stages) was compared.

### Detection of B vitamins

A total of 12 samples (two *Acer* species × two germination stages × three biological repetitions) were prepared for detection of each vitamin. Samples were ground in liquid nitrogen, and dry powder was homogenized. The method of Nishimune et al. (1988) was used for the detection of vitamin B1. Homogenization was performed in 200 mM potassium phosphate buffer (pH 7.0) containing 1% l-ascorbic acid and 1% l-cysteine. After centrifugation (10 min at 23,000×*g*), the reaction involved the oxidation of vitamin B1 in plant extract by cyanogen bromide (CNBr) followed by the addition of sodium hydroxide at a 5:3:2 v:v:v ratio. The fluorescence was measured at excitation and emission wavelengths (375 and 440 nm, respectively). The content of vitamin B2 was measured using the method described by Bartzatt and Wol (2014). The homogenate in sodium borate buffer (pH = 7.52) was centrifuged for 10 min at 36,000×*g*, and the absorbance of the supernatant was measured at 440 nm. Vitamin B9 was measured using a General Folic Acid ELISA Kit (MyBioSource, San Diego, CA, USA), and the manufacturer’s protocol enabled the detection of vitamin B9 in tissue homogenates prepared in 1× phosphate-buffered saline (PBS). The content of vitamin B12 was measured following the method of Li and Chen (Li and Chen 2000) based on the fluorescence of vitamin B12. The homogenate in 10 mM monopotassium phosphate buffer (pH = 7.0) was examined at λ_ex_ = 275 nm and λ_em_ = 305 nm. An Infinite M200 PRO (Tecan, Männedorf, Switzerland) plate reader and Magellan software were used for the measurements. The concentration of vitamins was calculated from calibration curves prepared for B1, B2, B9 and B12 vitamins. Statistically significant difference in vitamin content between sycamore and Norway maple was assessed according to *t*-test results (*P* < 0.05).

### Proteomics

A total of 12 samples (two *Acer* species × two germination stages × three biological repetitions) were prepared for liquid chromatography-tandem mass spectrometry (LC–MS/MS) analyses. Proteins were isolated in 50 mM triethylammonium bicarbonate (TEAB) and 5% SDS buffer (pH 8.5). Proteins (100 μg) were reduced (15 mM dithiothreitol, 30 min at 55 °C) and then alkylated (30 mM iodoacetamide, 15 min at room temperature (RT) in the dark). After the addition of phosphoric acid (1.2% w/v final concentration), the samples were diluted sevenfold with binding buffer (90% methanol in 100 mM TEAB, pH 7.55). The samples were loaded on a 96-well S-Trap™ plate (Protifi, Fairport, NY, USA), placed on top of a deepwell plate and centrifuged for 2 min at 1500×*g* at RT. After protein binding, the S-trap™ plate was washed three times with 200 μL of binding buffer and centrifuged for 2 min at 1500×*g* at RT. A new deepwell receiver plate was placed below the 96-well S-Trap™ plate, and protein digestion was performed overnight at 37 °C using 50 mM TEAB containing trypsin (1/62.5, w/w). Peptides were eluted three times: (i) with 80 μL of 50 mM TEAB, (ii) with 80 μL of 0.2% formic acid (FA) in water and (iii) with 80 μL of 0.2% FA in water/acetonitrile (ACN) (50/50, v/v). Each elution was performed by centrifuging for 2 min at 1500×*g*. The combined eluates were transferred to high-performance liquid chromatography inserts and dried in a vacuum concentrator.

#### LC–MS/MS analysis

The purified peptides were redissolved in 20 μL of loading solvent A (0.1% TFA in water/ACN (98:2, v/v)), and the peptide concentration was determined on a Lunatic instrument (Unchained Labs, Gent, Belgium). Peptides (2 μg) were injected for LC–MS/MS analysis on an Ultimate 3000 RSLCnano system connected in-line to a Q Exactive HF mass spectrometer (Thermo Fisher Scientific, Waltham, Massachusetts, USA). Trapping was performed at 10 μL min^−1^ for 4 min in loading solvent A on a 20 mm trapping column (made in-house, 100 μm internal diameter (I.D.), 5 μm beads, C18 Reprosil-HD, Dr Maisch, Germany). The peptides were separated on a 250 mm Waters nanoEase M/Z HSS T3 column with 100 Å, 1.8 μm and 75 μm inner diameters (Waters Corporation, Milford, MA, USA) kept at a constant temperature of 50 °C. For the germinated stage samples, peptides were eluted using a nonlinear gradient reaching 9% MS solvent B (0.1% FA in water/ACN (2:8, v/v)) in 15 min, 33% MS solvent B in 100 min, 55% MS solvent B in 135 min and 97% MS solvent B in 145 min at a constant flow rate of 300 nL min^−1^. This step was followed by a 35-min wash with 97% MS solvent B and re-equilibration with MS solvent A (0.1% FA in water). For the imbibed stage samples, peptides were eluted using a nonlinear gradient reaching 9% MS solvent B (0.1% FA in water/ACN (2:8, v/v)) in 15 min, 33% MS solvent B in 105 min, 55% MS solvent B in 145 min and 97% MS solvent B in 150 min at a constant flow rate of 300 nL min^−1^, followed by a 35-min wash at 97% MS solvent B and re-equilibration with MS solvent A (0.1% FA in water). The mass spectrometer was operated in data-dependent mode, automatically switching between MS and MS/MS acquisition for the 16 most abundant ion peaks per MS spectrum. Full-scan MS spectra (375–1500 m/z) were acquired at a resolution of 60,000 in the Orbitrap analyzer after accumulation to a target value of 3,000,000. The 16 most intense ions were isolated with a width of 1.5 m/z for fragmentation at a normalized collision energy of 28% after filling the trap at a target value of 100,000 for a maximum of 80 ms. MS/MS spectra (200–2000 m/z) were acquired at a resolution of 15,000 in the Orbitrap analyzer. QCloud was used to control instrument longitudinal performance during the analysis (Chiva et al. 2018).

#### Data analysis

Mass spectrometry data analysis was performed with MaxQuant (version 2.0.0) using the default search settings and a false discovery rate (FDR) of 1% at the peptide-spectrum match (PSM), peptide and protein levels. Spectra were searched against the *Acer* (maple trees, taxid: 4022) protein sequences in the UniProt database (database release version of February 2021) containing 33,470 sequences (https://uniprot.org) and the *Fagus sylvatica* (Beechnut, taxid: 28 930) protein sequences in the UniProt database (database release version of March 2021) containing 59,539 sequences (UniProt Consortium 2021). The settings of the main search included the following: the mass tolerance for precursor (4.5 p.p.m.), the mass tolerance for fragment ions (20 p.p.m.), enzyme specificity (C-terminal to arginine and lysine, also allowing cleavage at proline bonds with a maximum of two missed cleavages), fixed modification (cysteine carbamidomethylation) and variable modifications (oxidation of Met residues and acetylation of protein N-termini). A matching time window (0.7 min) and an alignment time window (20 min) were used for matching between runs. Only proteins with at least one unique or razor peptide were retained. The MaxLFQ algorithm was used for protein quantification with a minimum ratio of two unique or razor peptides. For the imbibed stage, a total of 157,362 PSMs were identified, resulting in 25,223 identified peptides corresponding to 3212 identified proteins. For the germinated stage, a total of 192,508 PSMs were performed, resulting in 20,846 identified peptides, corresponding to 3317 identified proteins. An in-house R script using the proteinGroups output table from MaxQuant was applied for data analysis of the shotgun results. Reverse database hits were removed. LFQ intensities were log2 transformed, and replicate samples were grouped. Proteins with fewer than three valid values in at least one group were removed, and missing values were imputed from a normal distribution centered on the detection limit (package DEP) (Zhang et al. 2018), yielding a list of 2182 quantified proteins in the imbibed stage and 2019 quantified proteins in the germinated stage. Protein abundance between pairs of sample groups (sycamore vs Norway maple at the imbibed and the germinated stages) was compared. Statistical testing for differences between two group means was performed using the limma package (Ritchie et al. 2015). Statistical significance for differential regulation was set to an FDR of < 0.05 and a fold change (FC) of > 4- or <0.25-fold (|log_2_FC| = 2). The results are presented in Tables S3–S14 available as Supplementary data at *Tree Physiology* Online. Z-scored LFQ intensities from significantly up- and downregulated proteins were plotted in a heatmap after unsupervised hierarchical clustering.

Functional analysis of up- and downregulated proteins was performed using the Protein Analysis Through Evolutionary Relationships (PANTHER) knowledgebase based on protein-coding gene classification information, namely, Gene Ontology (GO) annotations. A list of gene identification numbers (ID) corresponding to identified proteins (Tables S2, S4, S7 and S10 available as Supplementary data at *Tree Physiology* Online) was investigated in molecular function, biological process, cellular compartment and protein class categories (Mi et al. 2013). All of the IDs were derived from the UniProt database (UniProt Consortium 2021), specifically from the *Arabidopsis thaliana* genome, because the gene names of *Acer* are not recognized automatically, given the lack of the whole-genome sequence. At first, Arabidopsis homological proteins characterized with similarity ranging 80–100% were searched using Position-Specific Iterated Basic Local Alignment Search Tool, which is the best method of searching for functional homologues of a protein (Bhagwat and Aravind 2007). Then, corresponding gene IDs with the reviewed status (expertly annotated by UniProt curators) were extracted from the UniProt database. GO-based functional comparison was performed between sycamore and Norway maple species separately at the imbibed and germinated stages.

### PTM analysis

Further data analysis of the MetO-containing peptides was performed with an in-house R script using the oxidation (M) site output table from MaxQuant. Reverse database hits were removed, and the site table was expanded. The intensity values were log2 transformed. The median was subtracted, and replicate samples were grouped. MetO-containing peptides with fewer than three valid values in at least one group were removed, and missing values were imputed from a normal distribution centered on the detection limit (package DEP) (Zhang et al. 2018), leading to a list of 226 quantified Met oxidized peptides in the experiment, which were used for further data analysis. To compare Met-oxidized peptide abundance between pairs of sample groups (sycamore vs Norway maple at the imbibed and the germinated stages), statistical testing for differences between two group means was performed using the limma package as described above for the shotgun data.

## Results

### Metabolomic analyses

**Figure 1:**
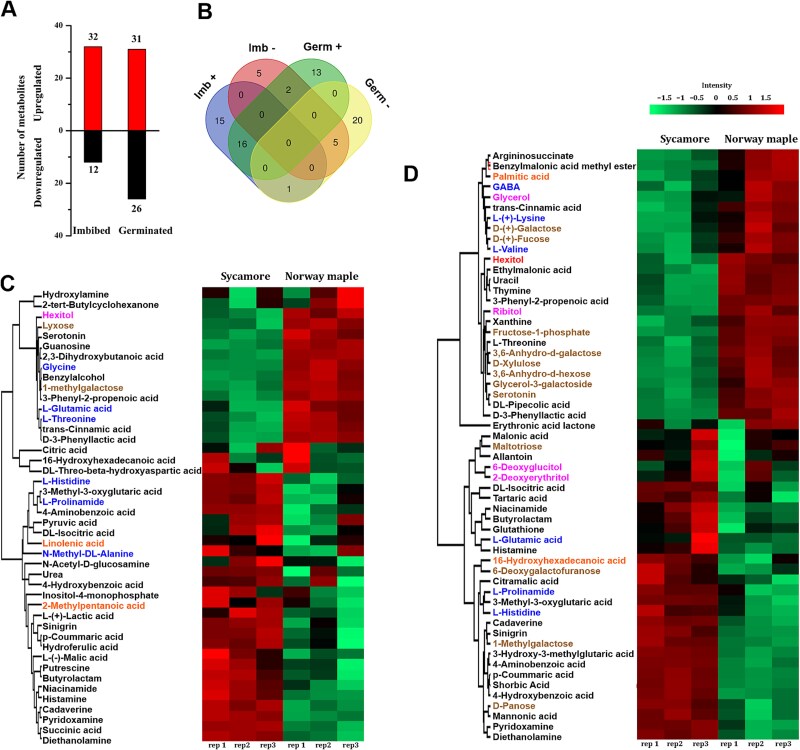
Number of identified and quantified DAMs at the imbibed (Imb) and germinated (Germ) stages in embryonic axes of sycamore as compared with Norway maple involving more abundant and less abundant metabolites (A). The number of common and unique metabolites with higher (+) and lower (−) abundance in sycamore as compared with Norway maple (B). (C, D) the significantly regulated metabolites at the imbibed (C) and germinated (D) stages are visualized in a heatmap after non-supervised hierarchical clustering of Z-scored log_2_ metabolite abundance. Orange names: fatty acids; brown names: carbohydrates; blue names: amino acids; pink names: sugar alcohols; black names: other compounds.

Using untargeted metabolite profiling, 1621 metabolites were detected, and 212 metabolites were successfully identified and quantified in embryonic axes of *Acer* imbibed and germinated seeds. A principal component (PC) analysis using all quantified metabolites as variables explained 95% of the data variance at the imbibed stage (Figure S1A available as Supplementary data at *Tree Physiology* Online) and 88% at the germinated stage (Figure S1B available as Supplementary data at *Tree Physiology* Online). Forty-four DAMs were detected at the imbibed stage (12 downregulated and 32 upregulated in sycamore compared with Norway maple), and 57 (31 upregulated and 26 downregulated in sycamore compared with Norway maple) were detected at the germinated stage (Figure 1A and C, Figure S2A and B, Tables S1 and S2 available as Supplementary data at *Tree Physiology* Online). Notably, 16 metabolites were more abundant at both the imbibed and the germinated stages, and 5 (i.e. serotonin) metabolites were continuously less abundant at both germination stages in sycamore (Figure 1B).

Some metabolites displayed prominent changes in peak intensity (Table S1 available as Supplementary data at *Tree Physiology* Online). The most abundant metabolites in sycamore at the imbibed stage compared with Norway maple at the imbibed stage included diethanolamine (DEA, intensity over 500-fold increase), pyridoxamine (over 400-fold increase), 4-aminobenzoic acid (over 90-fold increase). On the other hand, the least abundant metabolites in sycamore at the imbibed stage included trans-cinnamic acid (over 160-fold reduction), propenoic acid (60-fold reduction) and D-3-phenyllactic acid (over 40-fold reduction) (Table S1 available as Supplementary data at *Tree Physiology* Online). At the germinated stage, DEA was more than 200-fold more abundant in sycamore, whereas pyridoxamine and 1-methylgalactose were more than 80-fold more abundant.

**Figure 2:**
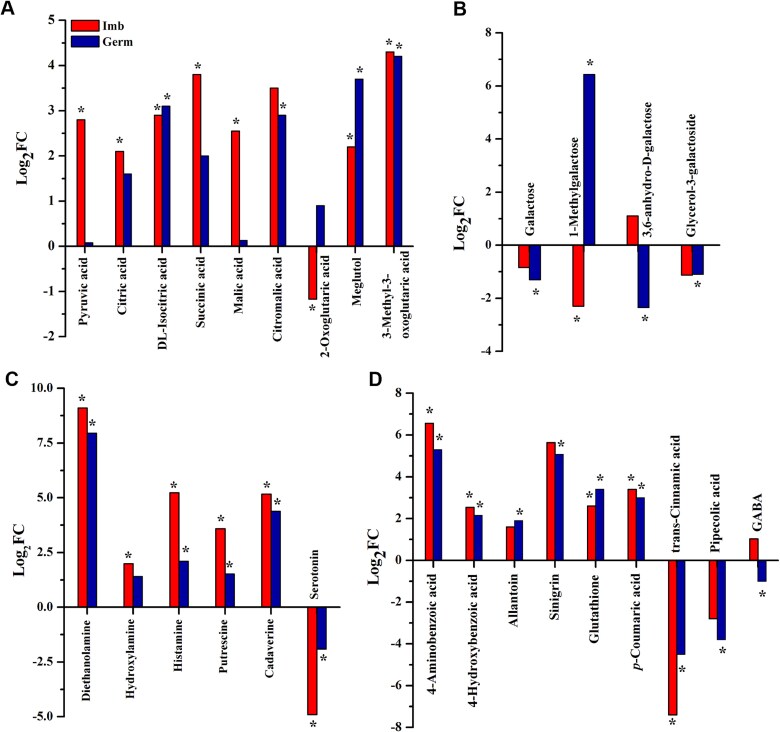
Differentially abundant metabolites found in embryonic axes of Norway maple and sycamore at the imbibed (Imb) and germinated (Germ) stages and involved in energy synthesis and Krebs cycle substrates, intermediates and their derivatives (A), the seed quality marker galactose and its derivatives (B), amines (C) and growth and defense stimulants (D). Positive or negative Log_2_FC values refer to more abundant or less abundant metabolites, respectively, in sycamore seeds. Data are the means of three replicates. ^*^Statistically significant difference in metabolite abundance between Norway maple and sycamore according to *t* test results (*P* < 0.05). Meglutol, 3-hydroxy-3-methyloglutaric acid.

At the imbibed stage, the aerobic respiration pathway appeared to be more active in sycamore, given that the levels of the end products of glycolysis and intermediates of the Krebs cycle were upregulated (Figure 2A). Additionally, the derivatives of tricarboxylic acid (TCA) cycle intermediates were particularly more abundant in sycamore at the germinated stage.

Several metabolites known in the literature as seed quality markers, vigour indicators, plant defense metabolites and growth stimulants were identified in our study and quantified at both the imbibed and germinated stages. The metabolites significantly changing at the germinated stage were used to assess the involvement of metabolites in seedling performance. The levels of galactose, a biomarker negatively correlated with seed vigour, and galactose derivatives were greater in Norway maple at the germinated stage (Figure 2B). Five important amines, except serotonin, were more abundant in sycamore (Figure 2C). The level of sinigrin, a glucosinolate synthesized from Met that functions in plant defense, was greater in Norway maple at both germination stages, whereas glutathione (GSH) was more abundant in sycamore (Figure 2D). Cinnamic acid and benzoic acid (BA) derivatives, including *p*-coumaric acid, were more abundant in sycamore at the germinated stage, whereas cinnamic and pipecolic acid (Pip) were more abundant in Norway maple at the germinated stage (Figure 2D). Additionally, allantoin was greater in sycamore, whereas GABA was greater in embryonic axes with protruded radicles of Norway maple seeds.

**Figure 3:**
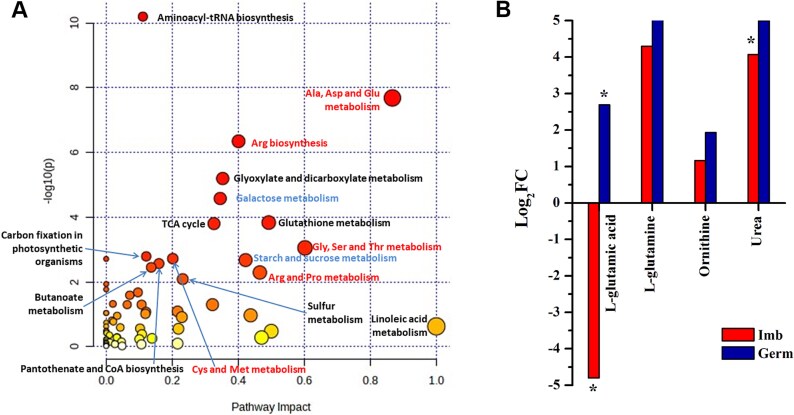
Enriched metabolic pathways involved in the regulation of the germination process at the imbibed (Imb) and germinated (Germ) stages in *Acer* species (A). The size of the circle indicates the impact of the pathway, while the circle colour represents the significance (the more intense the red colour, the lower the *P*-value). Predominant pathways related to amino acid metabolism are indicated in red font. Changes in the abundance of metabolites involved in ammonia metabolism (B) found at the imbibed and germinated stages in sycamore as compared with Norway maple. Data are the means of three replicates. ^*^Statistically significant difference in metabolite abundance between Norway maple and sycamore according to *t* test results (*P* < 0.05).

An analysis of the enriched metabolic pathways involving the identified metabolites indicated that the metabolism of 10 amino acids was the predominant pathway with the greatest impact on alanine, asparagine, glutamic acid and arginine (Figure 3A). Despite the opposite effects of glutamic acid at the imbibed and germinated stages, Gln, the product synthesized from glutamic acid and ammonia ions, was more abundant in sycamore than in Norway maple throughout the germination process. Analysis of ammonia metabolism intermediates revealed that only urea was more abundant in sycamore, particularly at the imbibed stage (Figure 3B).

### B vitamins

**Figure 4:**
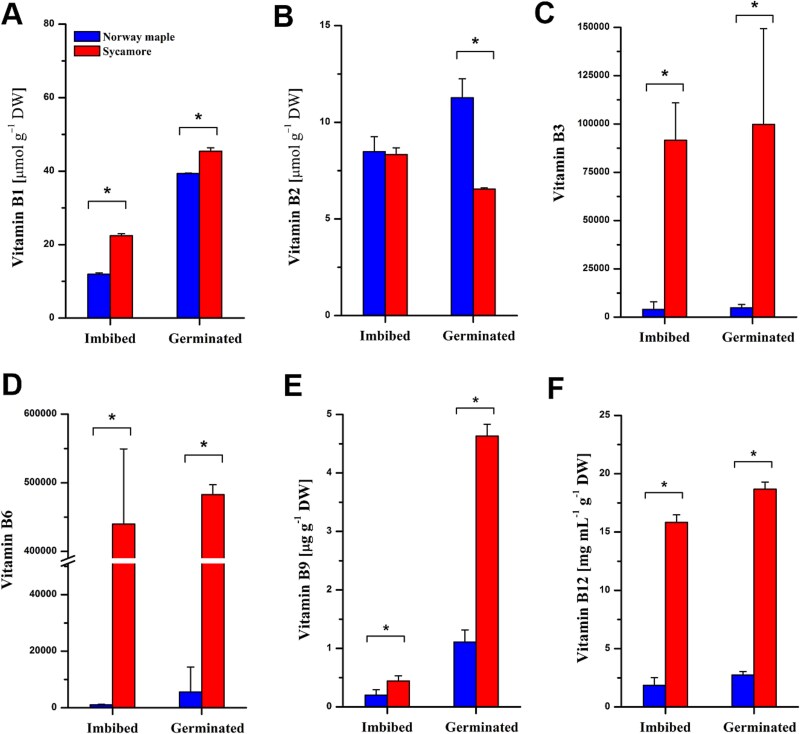
Differentially abundant B vitamins reported at the imbibed and the germinated stages in sycamore as compared with Norway maple: (A) B1, (B) B2, (C) B3, (D) B6, (E) B9 and (F) B12. The content of vitamins B1, B2, B9 and B12 is presented, whereas B3 and B6 vitamins are expressed as relative abundance from metabolite profiling. Data are the means of three replicates ± standard deviation. ^*^Statistically significant difference in metabolite abundance between Norway maple and sycamore according to t test results (*P* < 0.05).

In general, sycamore embryonic axes and embryonic axes with protruded radicles were found to have a greater vitamin B1 content (Figure 4A), which increased at the germinated stage. The vitamin B2 content was similar at the imbibed stage in both *Acer* species and increased at the germinated stage in Norway maple but decreased in sycamore (Figure 4B). In general, vitamin B3 and B6 levels were greater in sycamore; vitamin B3 levels increased by 20-fold during germination (Figure 4C), whereas vitamin B6 levels increased by over 400-fold at the imbibed stage and 80-fold at the germinated stage compared with Norway maple (Figure 4D). Vitamin B9 levels increased in both species during germination, and B9 levels were fourfold greater in sycamore seeds (Figure 4E). Vitamin B12 levels were sevenfold greater in sycamore during germination (Figure 4F).

### Proteome analyses

**Figure 5:**
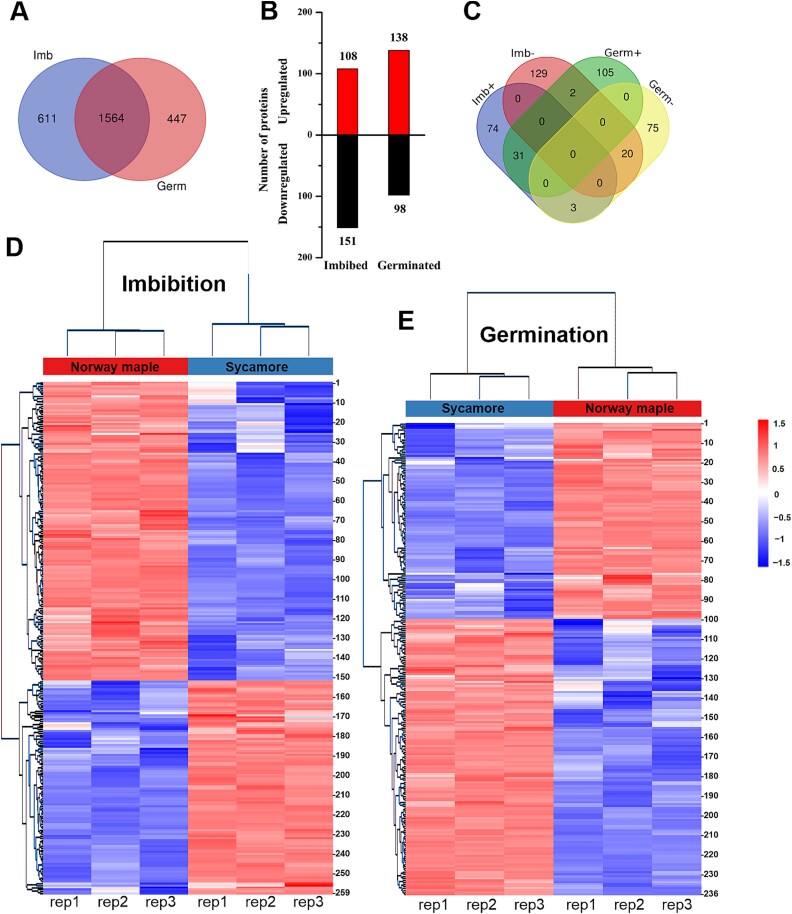
The number of common and unique proteins identified in both Norway maple and sycamore embryonic axes at imbibed (Imb) and germinated (Germ) stages (A). The number of differentially regulated proteins at the imbibed and germinated stages, where up- and downregulated protein numbers refer to sycamore as compared with Norway maple (B). (C) Venn diagram: the overlapping number stands for the mutual differentially regulated proteins between the upregulated (+) and downregulated (−) proteins at the Imb and Germ stages in sycamore as compared with Norway maple. The non-overlapping numbers specify the proteins unique to each condition. Significantly regulated proteins at the imbibed (D) and germinated (E) stages in sycamore are visualized in a heatmap after non-supervised hierarchical clustering of Z-scored log_2_ protein LFQ intensities. The numbers 1–259 (D) and 1–236 (E) refer to the individual proteins listed in Tables S3 and S6 available as Supplementary data at *Tree Physiology* Online.

To monitor protein changes during the germination of *Acer* seeds in an unbiased manner, we compared the proteomes of Norway maple and sycamore embryonic axes at the imbibed stage and embryonic axes with protruded radicles at the germinated stage using label-free quantitative mass spectrometry-based proteomics (LC–MS/MS). PC analysis using all quantified proteins as variables explained 86% of the variance in the data at the imbibed stage (Figure S1C available as Supplementary data at *Tree Physiology* Online) and the germinated stage (Figure S1D available as Supplementary data at *Tree Physiology* Online). Proteome analysis of the embryonic axes at the imbibed stage revealed 2128 quantified proteins, whereas analysis at the germinated stage revealed 2019 quantified proteins across the two species. Among these proteins, 611 and 447 were identified only in the imbibed stage or only in the germinated stage, respectively, in both species (Figure 5A). Statistical comparison of both species revealed 108 significantly (*P* < 0.05) upregulated and 151 significantly downregulated proteins at the imbibed stage in sycamore. At the germinated stage, 138 proteins were significantly upregulated and 98 proteins were downregulated in embryonic axes with protruded radicles of sycamore seeds as compared with Norway maple (Figure 5B, Figure S2C and D, Tables S3–S8 available as Supplementary data at *Tree Physiology* Online). Thirty-one proteins were upregulated in sycamore at both the imbibed and germinated stages, whereas 20 proteins were downregulated at both stages (Figure 5C, Tables S3–S5 available as Supplementary data at *Tree Physiology* Online).

### Functional analysis of proteins at the imbibed stage

**Figure 6:**
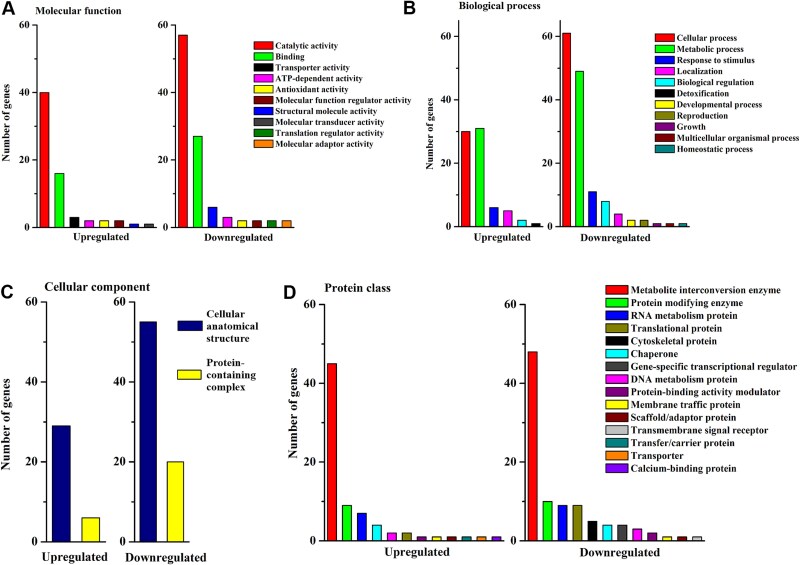
Functional analysis of significantly regulated proteins in embryonic axes of sycamore seeds at the imbibed stage as compared with Norway maple in terms of (A) molecular function, (B) biological process, (C) cellular compartment and (D) protein class based on GO annotation. For child categories, please see Table S5 available as Supplementary data at *Tree Physiology* Online.

Significantly regulated proteins at the imbibed stage were further analyzed in terms of their molecular function, biological process, cellular compartment and protein class based on their GO annotations. Proteins with catalytic activity (GO:0003824) were the largest group according to the molecular function annotation of both up- and downregulated proteins (Figure 6A). In this group, enzymes with transferase activity (GO:0016772) were the largest subclass downregulated in sycamore, whereas oxidoreductases (GO:0016491) were the largest subclass of upregulated proteins (Table S5 available as Supplementary data at *Tree Physiology* Online). The protein-binding category in the upregulated proteins in sycamore involved subclasses of proteins displaying the highest binding activity for ions (Table S5 available as Supplementary data at *Tree Physiology* Online). In contrast, the binding category in the downregulated sycamore proteins at the imbibed stage involved the carbohydrate derivative (GO:0097367) subclass (17%), which was not present in the group of upregulated proteins. Functional analysis according to biological process (Figure 6B) revealed that the metabolic processes category involved nitrogen compounds in both up- and downregulated proteins in sycamore (Table S5 available as Supplementary data at *Tree Physiology* Online). The group of downregulated proteins in sycamore imbibed embryonic axes was characterized by a larger number of subclasses than the group of upregulated proteins and included unique subclasses related to growth, cell division, reproduction, glycosylation and methylation (Table S5 available as Supplementary data at *Tree Physiology* Online).

The protein-containing complex (GO:0032991), a less numerous subclass than cellular anatomical structure (GO:0110165) subclass in cellular component (GO:0005575) terms (Figure 6C), contained mainly ribonucleoprotein and nuclear protein-containing complex (GO:0140513) subclasses among upregulated sycamore proteins and catalytic complex (GO:1902494) subclass among downregulated sycamore proteins. The metabolite interconversion enzyme (PC00262) category constituted 60% of the upregulated proteins and 48% of the downregulated proteins in the imbibed embryonic axes of sycamore seeds (Figure 6D).

Considering that (i) the majority of GO-based categories were found in up- and downregulated proteins and (ii) functional groups of proteins were oppositely regulated in sycamore and in Norway maple, we focused on specific proteins in the above-indicated GO-based categories and subclasses presented in Figure 6. Imbibed Norway maple embryonic axes displayed organelle biogenesis, as chloroplast formation was suggested by significantly upregulated levels of proteins involved in thylakoid formation (protein thylakoid formation 1), synthesis of chloroplastic Fe-S centers (Nfu/NifU), ribosome assembly (30S ribosomal protein (RP) S8 and S17, 50S RP L20) and photosynthetic activity, such as ferredoxin-nicotinamide adenine dinucleotide phosphate (NADP) reductase, proteins related to chlorophyll synthesis (porphobilinogen synthase), phytocyanin and starch synthase (Table S4 available as Supplementary data at *Tree Physiology* Online). In contrast, imbibition induced an increase in sycamore chloroplastic proteins involved in the elimination of oxidized nucleotides in chloroplasts (nudix hydrolase 26) and fatty acid export from plastids (protein fatty acid export). Similarly, different processes were upregulated by imbibition in the mitochondria of *Acer* embryonic axes. Imbibition induced the expression of Norway maple proteins associated with the reactivation of mitochondria and involved in the synthesis of mitochondrial Fe-S centers (NFU1 and BOL), the transport of cytosolic proteins into mitochondria (TIM44) and the synthesis of mitochondrial proteins (elongation factor Ts). Imbibed sycamore embryonic axes exhibited elevated levels of one protein possibly governing mitochondrial division, mitochondrial fission 1 protein.

Transcription (rRNA biogenesis protein RRP5) and rRNA processing involving the healing and sealing of RNA and splicing (THO complex subunit 4A-like, RNA ligase/cyclic nucleotide phosphodiesterase and WD repeat-containing protein 55) were upregulated in Norway maple at the imbibed stage (Table S4 available as Supplementary data at *Tree Physiology* Online). In sycamore seeds, only proteins involved in the regulation of splicing (small nuclear ribonucleoprotein E, polyadenylate-binding RBP45B-like protein, U6 snRNA-associated Sm-like protein LSm4) were upregulated. Moreover, the synthesis of RNA polymerase II (FCP1), ribosome formation (nucleolar complex protein 2), initiation of translation (eIF-2B GDP-GTP exchange factor subunit gamma) and elongation of translation (60S acidic RP P2) were induced in imbibed Norway maple seeds (Table S4 available as Supplementary data at *Tree Physiology* Online). In the sycamore seeds, the sorting of cargo proteins (BRO1) and integration of membrane proteins into the lipid bilayer (plug translocon) were upregulated.

The processes associated with growth induction were also differentially regulated at imbibed stage. Growth initiation in Norway maple seeds was manifested by an increase in proteins involved in cell division (FtsZ, cyclin-dependent kinases and DNA replication licencing factor MCM7), initiation of plant growth (GATA transcription factor, PLAT domain-containing protein 1) and leaf growth and morphogenesis (ultraviolet-B receptor, cellulose synthase). However, in sycamore, proteins associated with trichome branching (STICHEL), cell wall integrity (cell wall integrity and stress response component 4 like, pectinesterase) and loosening of the cell wall (expansin) were upregulated (Table S4 available as Supplementary data at *Tree Physiology* Online).

Importantly, seven oxygenases were upregulated in the imbibed sycamore embryonic axes (Tables S3 and S4 available as Supplementary data at *Tree Physiology* Online). Additionally, specifically in sycamore at the imbibed stage, the ABA signal transduction pathway in response to osmotic stress was activated and involved proteins related to the induction of drought tolerance (myosin-like protein (MLP)-like proteins 423, 34, 328 and 28, copper amine oxidase) and to the mitigation of osmotic stress-induced inhibition of plant growth (ABA receptor PYL1, protein detoxification). Sycamore at the imbibed stage also exhibited increased levels of proteins involved in plant innate immunity (salicylic acid-binding protein 2-like), tolerance to high-temperature stress (multiprotein-bridging factor 1c, Hikeshi), basal resistance against pathogens (neomenthol dehydrogenase), oxidative stress response (DJ-1D, thioredoxin H1, GSH S-transferase DHAR2-like), NAD(P)/NAD(P)H homeostasis (NADP-dependent alkenal double bond reductase P2), nonselective autophagy (protein NBR1 homolog) and a reduction in protein disulphide bonds (gamma-interferon-responsive lysosomal thiol protein) (Table S4 available as Supplementary data at *Tree Physiology* Online).

### Functional analysis of proteins at the germinated stage

**Figure 7:**
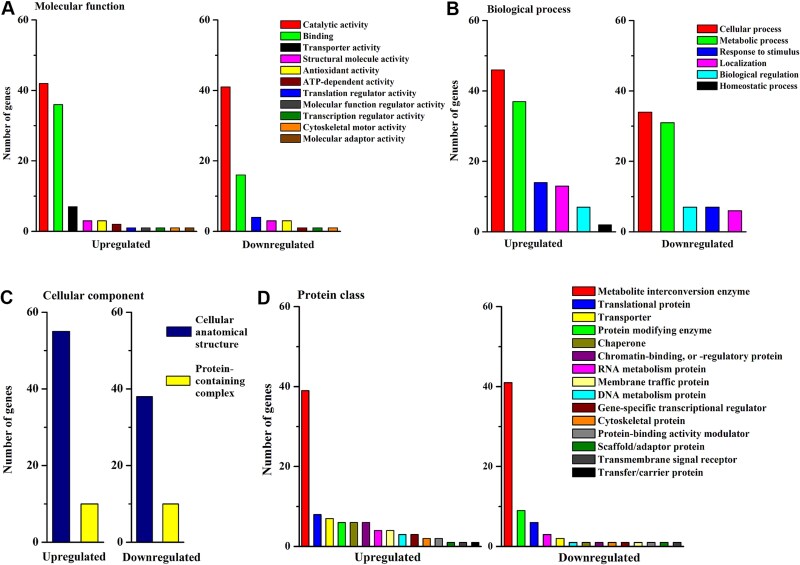
Functional analysis of proteins significantly regulated in embryonic axes with protruded radicles of sycamore seeds at the germinated stage as compared with Norway maple in terms of (A) molecular function, (B) biological process, (C) cellular compartment and (D) protein class based on GO annotation. For child categories, please see Table S8 available as Supplementary data at *Tree Physiology* Online.

The catalytic activity of oxidoreductase and hydrolase (GO:0016787) was the major subclass among both up- and downregulated proteins in sycamore at the germinated stage compared with Norway maple, respectively, in terms of molecular function (Figure 7A). The binding category was twice as common in the group of upregulated proteins in sycamore and involved proteins binding to unfolded proteins, whereas downregulated proteins included proteins binding to ions, carbohydrates and lipids (Table S8 available as Supplementary data at *Tree Physiology* Online). Analyses of biological process terms demonstrated that cellular and metabolic processes (GO:0044237) comprised 61% and 76% of the analyzed annotations in sycamore up- and downregulated proteins, respectively (Figure 7B). The metabolic process category involved four subclasses with >20 proteins each, including organic substance, primary, cellular and nitrogen compound metabolic processes in both up- and downregulated proteins. The analysis of protein assigned to play a role in cellular component (GO:0005886) (Figure 7C) revealed that complexes of membrane and mitochondrial proteins, together with proteasome components, were present among the upregulated sycamore proteins, whereas downregulated proteins involved nuclear catalytic complexes (Table S8 available as Supplementary data at *Tree Physiology* Online). Oxidoreductases constituted 42% of the upregulated proteins and 58% of the downregulated proteins in terms of protein class (Figure 7D).

Analysis of regulated proteins at the germinated stage (Tables S6–S8 available as Supplementary data at *Tree Physiology* Online) suggested that Norway maple seeds contained increased levels of several chloroplastic enzymes (i.e. starch synthase, biotin carboxylase, 5-methyltetrahydropteroyltriglutamate-homocysteine S-methyltransferase) and a protein associated with the chloroplastic redox regulon (short-chain dehydrogenase TIC 32). In contrast, sycamore embryonic axes with protruded radicles exhibited many upregulated chloroplastic proteins involved in plastid differentiation (ankyrin-3 and outer envelope pore proteins 16 and 16–3) and chloroplast biogenesis (translocase), as well as elevated levels of chlorophyll a-b binding protein and a protein suppressing chlorophyll degradation under osmotic stress (BURP domain protein RD22) (Table S7 available as Supplementary data at *Tree Physiology* Online). The upregulated sycamore chloroplastic proteins included enzymes related to amino acid biosynthesis (ornithine carbamoyltransferase, homoserine dehydrogenase), ribosome biogenesis in chloroplasts (DEAD-box ATP-dependent RNA helicase 3), metal homeostasis (protein CutA), redox homeostasis (2-Cys peroxiredoxin BAS1), and proteins related to general growth processes (protein fatty acid export 3) and leaf development (reticulata-related 3 (RER3)). No specific protein associated with mitochondrial structure or activity was upregulated in Norway maple, whereas in sycamore, structural proteins (mitochondrial ATP synthase 6 kDa subunit, 4Fe-4S ferredoxin-type domain-containing protein, electron transfer flavoprotein subunit alpha, uncoupling protein) functioning as channels (outer membrane protein porin of 36 kDa, adenosine diphosphate (ADP)/adenosine triphosphate (ATP) translocase) or stabilizers for newly synthesized mitochondrial proteins (prohibitin) were upregulated at the germinated stage.

The upregulated proteins in Norway maple at the germinated stage related to the regulation of plant development included enzymes associated with the cell cycle (cell division cycle protein 48), plant growth (phosphoethanolamine N-methyltransferase), survival in response to nutrient and hormonal signals (target of rapamycin complex subunit LST8) and one enzyme involved in ET biosynthesis (1-aminocyclopropane-1-carboxylate oxidase 2) (Table S7 available as Supplementary data at *Tree Physiology* Online). Germinated sycamore seeds contained increased levels of proteins regulating cell morphogenesis (SPIRRIG), cell expansion (wall-associated receptor kinase) and synthesis of cell wall in rapidly growing tissues (3-deoxy-manno-octulosonate cytidylyltransferase), plant growth (STICHEL) and root development (cochaperone protein p23).

Gene expression and protein biosynthesis in Norway maple at the germinated stage were possibly enhanced by the transcription factor TT8, translation initiation (2A, 4A) and elongation (1-gamma, 2) factors and intracellular trafficking of protein and RNA between the nucleus and the cytoplasm (Ran guanine nucleotide release factor) (Table S7 available as Supplementary data at *Tree Physiology* Online). Sycamore displayed high levels of a protein possibly stimulating basal transcription to a fully activated level (upstream activation factor subunit spp27) and an enzyme predicted to be involved in pre-mRNA maturation (polyadenylate-binding protein RBP47B) at the germinated stage.

Stress alleviation was an important process upregulated at the germinated stage in sycamore through the activation of proteins regulating plant defense (salicylic acid-binding protein 2-like), oxidative stress (DJ-1D), protection during the desiccation stage (Em-like protein GEA1), reduction of oxidized Met (MsrB5) and ABA/stress-induced protein (HVA22-like protein, AWPM-19-like, aspartic protease in guard cell 2). The stress-related proteins and proteins involved in the control of water stress-induced cell death (Serpin-ZX) were upregulated in Norway maple. *Acer* seeds also differed in the type of induced GSH S-transferases. Types U7, U25 and Z1 DHAR2-like were detected in sycamore, whereas the T1 type was detected in Norway maple at the germinated stage (Table S7 available as Supplementary data at *Tree Physiology* Online).

### Proteins containing MetO in *Acer* embryonic axes

**Figure 8:**
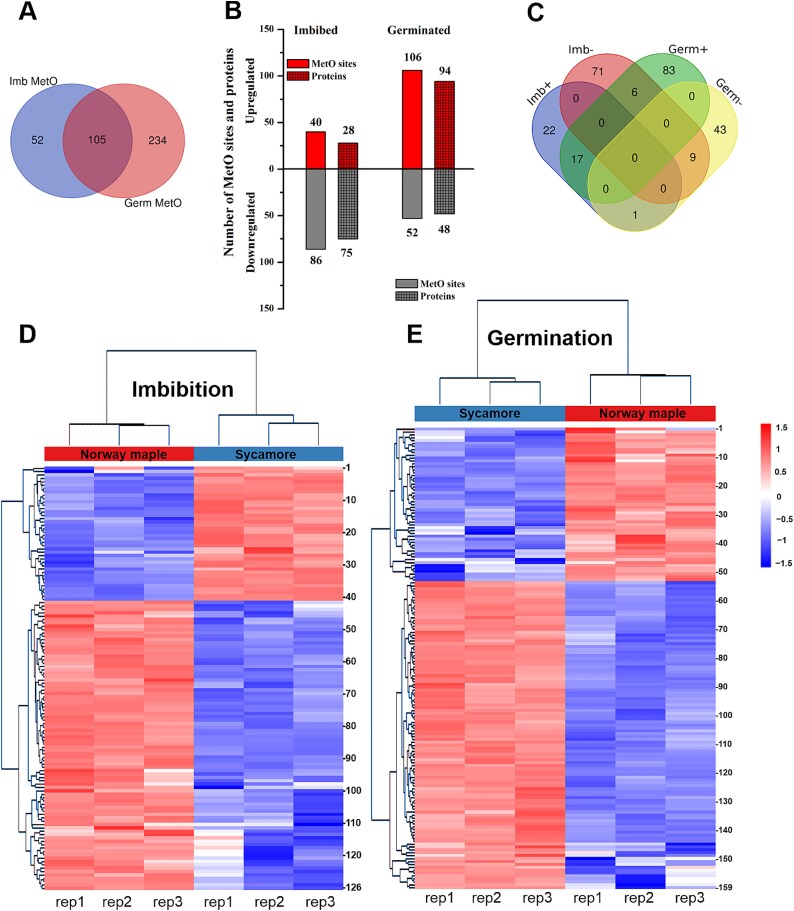
Detection and quantification of protein MetO sites in embryonic axes of imbibed (Imb) and germinated (Germ) Norway maple and sycamore seeds. (A) Common and unique proteins containing MetO sites identified in the imbibed and germinated embryonic axes. (B) Number of differentially regulated MetO sites in peptides and corresponding proteins at the imbibed and germinated stages, where up- and downregulated MetO site/protein numbers refer to sycamore as compared with Norway maple. (C) Venn diagram showing the number of unique and overlapping proteins with increased (+) and decreased (−) MetO levels at both germination stages. (D, E) the significantly regulated MetO peptides at the imbibed (D) and germinated (E) stages were visualized in a heatmap after non-supervised hierarchical clustering of Z-scored log_2_ peptide intensities. The numbers 1–126 (D) and 1–159 (E) refer to the individual proteins listed in Tables S7 and S9 available as Supplementary data at *Tree Physiology* Online.

In addition to protein-level changes, we also targeted oxidized Met residues in proteins identified at the imbibed and germinated stages. Therefore, Met oxidation was included as a variable modification during database searches, resulting in 238 and 506 quantified MetO-containing peptides in the imbibed and the germinated stages in both *Acer* species, respectively (Tables S9 and S12 available as Supplementary data at *Tree Physiology* Online). A PC analysis using all quantified proteins with regulated MetO sites as variables explained 89% of the data variance at the imbibed stage (Figure S1E available as Supplementary data at *Tree Physiology* Online) and 86% at the germinated stage (Figure S1F available as Supplementary data at *Tree Physiology* Online). Among these quantified MetO sites, 52 and 234 were detected only in the imbibed and germinated stages, respectively, in both species (Figure 8A). Comparing the abundances of these peptides between sycamore and Norway maple led to 40 significantly (*P* < 0.05) upregulated MetO sites (from 28 proteins) and 86 significantly downregulated sites (from 75 proteins) at the imbibed stage in sycamore (Table S10 available as Supplementary data at *Tree Physiology* Online). At the germinated stage, 106 MetO sites (from 94 proteins) were significantly upregulated and 52 sites (from 48 proteins) were downregulated compared with Norway maple (Figure 8B, S2E–F available as Supplementary data at *Tree Physiology* Online). Our results demonstrated that one protein might be oxidized at multiple Met sites and that different MetO sites in one protein might exhibit increased or decreased MetO levels in proteins at specific germination stages when compared between sycamore and Norway maple (Figure 8C). Ten proteins (i.e. STICHEL, DJ-1B, GASA14 and ferritin) displayed increasing MetO levels, and nine (i.e. one initiation and three elongation factors for translation, poly (ADP-ribose) polymerase (PARP) and zinc finger BED domain-containing protein RICESLEEPER) displayed lower MetO levels both at the imbibed and germinated stages in sycamore (Figure 8C).

### Proteins containing dynamically changing MetO levels at the imbibed stage

**Figure 9:**
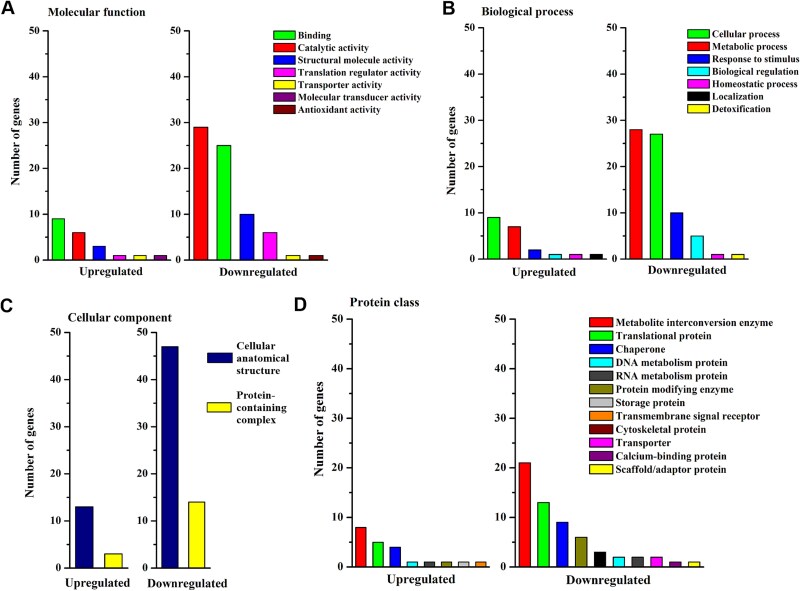
Functional analysis of proteins containing MetO significantly regulated in embryonic axes of sycamore seeds at the imbibed stage as compared with Norway maple in terms of (A) molecular function, (B) biological process, (C) cellular compartment and (D) protein class based on GO annotation. For child categories, please see Table S11 available as Supplementary data at *Tree Physiology* Online.

The enriched subclasses of molecular function class were similar in proteins containing increased and decreased MetO levels (Figure 9A). However, the ion binding (GO:0043167) subclass was enriched in proteins with upregulated MetO sites in sycamore, whereas binding to nucleic acids (GO:0003676) was the main subclass among the downregulated proteins in this species (Table S11 available as Supplementary data at *Tree Physiology* Online). Functional analyses using biological process terms (Figure 9B) revealed that proteins containing significantly changing MetO levels were involved in many metabolic processes, and the subclass nitrogen compound metabolic process (GO:0006807) was equally important in both the up- and downregulated groups of proteins. Mitochondrion and ribosome were organelles enriched in the protein-containing complex category among the sycamore proteins with decreasing and increasing MetO levels, respectively, in relation to cellular compartment terms (Figure 9C, Table S11 available as Supplementary data at *Tree Physiology* Online). The functional classification for the protein class (Figure 9D) revealed that proteins with relatively high MetO levels included RPs, whereas ribosome and proteasome terms were found among proteins with relatively low MetO levels.

### Proteins containing dynamically changing MetO levels at the germinated stage

**Figure 10:**
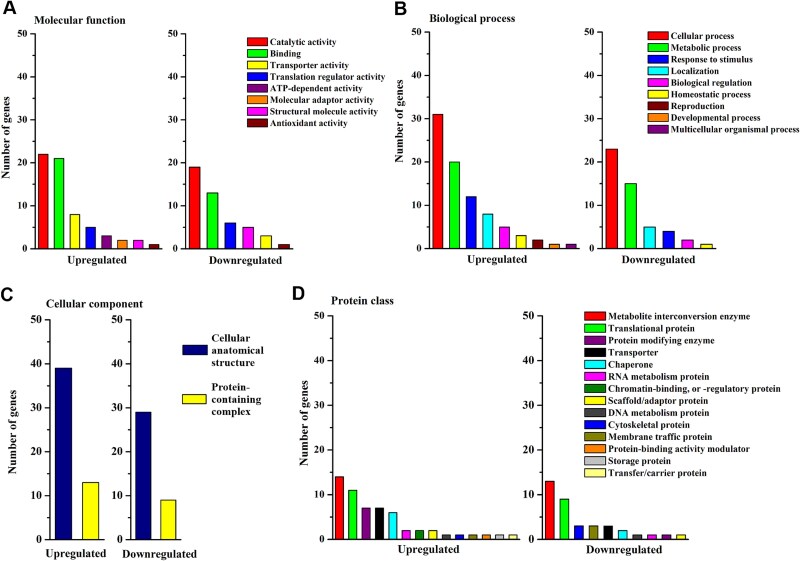
Functional analysis of proteins containing MetO significantly regulated in embryonic axes with protruded radicles of sycamore seeds at the germinated stage as compared with Norway maple terms of (A) molecular function, (B) biological process, (C) cellular compartment and (D) protein class based on GO annotation. For child categories, please see Table S14 available as Supplementary data at *Tree Physiology* Online.

Proteins with lower MetO levels included proteins with structural molecule activity (GO:0005198) related to the cytoskeleton, a unique subclass in molecular function terms, and the cell wall organization (GO:0071555) subclass in terms of biological processes (Figure 10A and B, Table S14 available as Supplementary data at *Tree Physiology* Online). Cytoskeletal proteins undergoing Met/MetO redox changes involved annexin at the imbibed stage (Tables S9–S11 available as Supplementary data at *Tree Physiology* Online) and actins and profilin at the germinated stage (Tables S12–S14 available as Supplementary data at *Tree Physiology* Online). Concerning metabolic processes, proteins containing relatively high and low MetO levels were assigned to one and three subclasses related to nitrogen compounds, respectively (Table S10 available as Supplementary data at *Tree Physiology* Online). For cellular compartments, only proteins with lower MetO levels in sycamore related to a subclass of membrane catalytic complexes implicated in ATP synthesis in the inner mitochondrial membrane (Figure 10C, Table S14 available as Supplementary data at *Tree Physiology* Online). The categories of protein class were similar and displayed different proportions in the up- and downregulated groups (Figure 10D).

MetO dynamics in proteins also differed at the imbibed and/or germinated stages in both *Acer* species in terms of seed defense proteins (late embryogenesis abundant proteins (i.e. D-29, D-34, 14-like), dehydrins, small heat shock proteins, chaperones and low-temperature-induced 65 kDa protein), including proteins affecting redox status (peroxidases, peroxiredoxins, GSH transferase and gibberellin-regulated protein 14 (GASA14)). Proteins related to the chloroplast biogenesis and organizations were revealed to be under Met/MetO redox regulation. Different chloroplastic proteins at the imbibed stage (i.e. protochlorophyllide-dependent translocon component 52) and at the germinated stage (i.e. chlorophyll a-b binding protein, outer envelope pore protein 16–2 (OEP16)) and during whole germination (DJ-1 homolog B (DJ-1B)) as well as many chloroplastic enzymes, including those responsible for fatty acid synthesis and enoyl-(acyl-carrier-protein) reductases, displayed dynamically changing MetO levels (Tables S9–S13 available as Supplementary data at *Tree Physiology* Online).

## Discussion

To explore why sycamore seeds germinate earlier and produce larger numbers of seedlings characterized by better growth and greater biomass under controlled conditions than Norway maple seeds, we used two omics methods. Proteome analyses suggested the major importance of metabolite interconversion enzymes in the regulation of seed germination (Figures 6 and 7). In addition, metabolome analyses highlighted the significance of the metabolism of amino acids in germinating *Acer* species (Figure 3A). The protein content in mature sycamore seeds is much greater than that in Norway maple seeds, both in embryonic axes and cotyledons (Stolarska et al. 2020). Higher amounts of seed storage reserves in sycamore implicate higher levels of building blocks and resources for energy synthesis. We hypothesize that a greater abundance of nitrogen sources allows sycamore seeds to invest in growth (faster germination, greater seedling biomass) and in seed defense, as reflected by greater levels of metabolites (Figure 2) and antioxidants (Alipour et al. 2021), particularly given that seedling growth results from seed storage reserves (Naegle et al. 2005) and that sycamore cotyledons contain twice the amount of protein as those of Norway maple (Stolarska et al. 2020). Functional analysis of proteins at the imbibed stage revealed that the metabolic processes associated with nitrogen compounds were significantly upregulated only in sycamore. At the germinated stage, metabolic processes of nitrogen compounds were equally well represented in both up- and downregulated proteins in both embryonic axes of Norway maple and sycamore. These results support the importance of nitrogen metabolism for *Acer* seed germination, which is particularly beneficial for the sycamore species.

### Metabolic control of seed germination

Seed germination is controlled by carbon and nitrogen availability (Osuna et al. 2015). In our study, higher nitrogen resources in sycamore contributed to higher levels of the majority of identified amines, particularly at the germinated stage (Figure 2C). The higher the polyamine content is, the greater the plant growth and metabolism are (Chen et al. 2018, González-Hernández et al. 2022, Sheng et al. 2022). Recently, the ornithine–asparagine–polyamine metabolite module was suggested to be important for seed germination (Guo et al. 2021). Although Norway maple and sycamore displayed similar levels of ornithine and asparagine, they greatly differed in their levels of amines. Polyamines contribute to faster germination (Lando et al. 2020). The diamines putrescine (Put) and cadaverine (Cad) were more abundant in sycamore at the germinated stage (Figure 2D). Put increases shoot growth (Nahed and Lobna 2009), whereas Cad contributes to root system architecture, mainly root branching (Jancewicz et al. 2016). Both Cad and Put might contribute to increased postgerminative growth in sycamore because elevated polyamine content enhances radicle protrusion (Zandoná et al. 2021). DEA, a secondary amine that scavenges H_2_S, is known to assist in seed germination under stress conditions (Ibrahim et al. 2021) and captures CO_2_, the elevated levels of which decrease the concentrations of proteins in seeds (Li et al. 2018). In this context, extremely high DEA levels might explain the higher storage protein content in sycamore seeds (Stolarska et al. 2020). Higher glutamic acid in Norway maple at the imbibed stage and sycamore at the germinated stage contrasted with comparable Gln levels, indicating that nitrogen metabolism is active in both species. However, sycamore contains more nitrogen storage reserves and possibly has greater nitrogen utilization efficiency (Figure 3B), as reflected in its amine levels and metabolism. The only amine that was more abundant in Norway maple was serotonin, which was three times more abundant at the germinated stage (Table S1 available as Supplementary data at *Tree Physiology* Online, Figure 2C). Serotonin is involved in plant morphogenesis (Ramakrishna et al. 2011) and the stimulation of germination in orthodox seeds (Erland et al. 2015). It is possible that higher serotonin levels enhanced germination rates in Norway maple seeds to levels similar to those of sycamore seeds (Alipour et al. 2021), but the beneficial effect was no longer present at the seedling stage (Alipour et al. 2023). Similarly, the level of GABA, a signalling molecule that significantly increases the levels of soluble sugars and antioxidants (i.e. ascorbic acid and GSH) in seeds and thereby improve germination (Zhou et al. 2021), was twofold increase in Norway maple at the germinated stage (Figure 2D); however, the levels of ascorbic acid and GSH were both greater in sycamore at the germinated stage (Alipour et al. 2021).

Nitrogen mobilization and antioxidant/defense responses were reported to be essential for germinating seeds and seedlings of rice (Guo et al. 2021). Seeds are equipped with various defense metabolites. The level of sinigrin, a major glucosinolate involved in plant defense, was greater (log_2_FC = 5) in sycamore at the imbibed and germinated stages (Figure 2D). Sinigrin, which exhibits antimicrobial activity (Bischoff 2021), might contribute to better performance at early seedling developmental stages. Pip, a nonprotein amino acid, regulates seed resistance against fungal infections (Sharma et al. 2022), and Norway maple at the germinated stage was better equipped with this molecule (Figure 2D). Additionally, ABA signalling controls plant development and the survival of germinating seeds and consequently seedlings under unfavourable conditions (Pri-Tal et al. 2024). Therefore, the upregulation of ABA/stress-induced proteins in sycamore at the germinated stage (Table S7 available as Supplementary data at *Tree Physiology* Online) may contribute to better endogenous protection in sycamore postgerminative growth and greater seedling performance.

Organic salts based on *p*-aminobenzoic acid are considered plant growth stimulants (Sumalan et al. 2020). 4-Aminobenzoic acid was more abundant in sycamore seeds (Table S1 available as Supplementary data at *Tree Physiology* Online, Figure 2D). Cinnamic acid, BA and their derivatives (i.e. *p*-coumaric acid and *p*-hydroxybenzoic acid) reduce canola seedling biomass without affecting germination percentage (Ng et al. 2003). Similar levels of BA but higher levels of cinnamic acid were found in Norway maple germinating seeds (Table S1 available as Supplementary data at *Tree Physiology* Online). This finding might be related to the lower biomass of Norway maple seedlings than of sycamore seedlings (Alipour et al. 2021). Interestingly, BA derivatives such as 4-hydroxybenzoic acid and *p*-coumaric acid were found at relatively high levels in both imbibed and germinated sycamore embryonic axes (Figure 2D).

Enhanced release of TCA intermediates causes mitochondrial dysfunction. The TCA metabolite 3-hydroxy-3-methylglutaric acid (HMG) induces oxidative stress and disrupts redox and energy homeostasis by decreasing the activity of TCA enzymes (da Rosa et al. 2020). Induced expression of 3-hydroxy-3-methylglutaryl-CoA lyase (HMGL) delays the germination of Arabidopsis seeds (Hemmerlin et al. 2019). In this context, lower levels of HMG, the substrate of HMGL, might explain why Norway maple seeds require more time to complete germination. Moreover, higher hydroxylamine levels in sycamore at the imbibed stage (Figure 2C) might be related to the fact that sycamore seeds accomplished germination faster than Norway maple seeds, as hydroxylamines can promote seed germination by inhibiting H_2_O_2_ decomposition (Hendricks and Taylorson 1974) and by affecting antioxidant enzyme activity (Rodrigues et al. 2024). Indeed, H_2_O_2_ levels were greater in imbibed and germinated sycamore seeds than in Norway maple seeds (Wojciechowska et al. 2020*b*). Other studies have demonstrated that more succinate and malate (gluconeogenic pathway precursors) are associated with earlier germination (Li et al. 2022). Sycamore contained relatively high levels of both metabolites at the imbibed stage (Figure 2), which could accelerate the germination process at the initial stage in sycamore because at the end of germination, the levels of malate and succinate were similar in the embryonic axes with protruded radicles of both analyzed species.

### The role of B vitamins in the regulation of *Acer* seed germination

Except for B12, plants synthesize B vitamins (Roje 2007). Elevated vitamin B6 content results in increased plant biomass and resistance to abiotic stress (Vanderschuren et al. 2013). Arabidopsis mutants with decreased vitamin B6 levels are characterized by impaired seedling establishment (Wagner et al. 2006). Higher levels of vitamin B6 (Figure 4D) in sycamore at the germinated stage might explain why the success of seedling establishment was 20% greater in this species than in Norway maple (Alipour et al. 2023). Seeds soaked in vitamins B1, B2 and B12 exhibited greater germination rates and seedling growth (Neumann et al. 1996, Xin Chi et al. 2021). Most likely, the higher content of vitamins B1 and B12 in sycamore at the germinated stage (Figure 4A and F) contribute to the greater biomass of the sycamore seedlings. Vitamin B3 is the precursor of NAD^+^ (Gakière et al. 2018). The extremely high levels of vitamin B3 in sycamore (Figure 4C) might indicate that the de novo synthesis or salvage pathway for NAD^+^ biosynthesis was not predominantly activated in sycamore, as the pool of NAD was twofold lower at the imbibed stage and 13 times lower at the germinated stage in sycamore than in Norway maple (Alipour et al. 2021). Vitamin B9 was found to increase the protein content in seeds during the germination process, and any disturbances in the metabolism of this vitamin in plants lead to significant growth inhibition (Zeboon and Baqir 2023). In this context, fourfold more vitamin B9 in sycamore than in Norway maple at the germinated stage might explain the better growth of sycamore seedlings than Norway maple seedlings (Alipour et al. 2023).

### Proteomic evidence for the biogenesis and organization of chloroplasts and mitochondria in germinating *Acer* embryonic axes

Proteomics of the germination of tree seeds demonstrated that energy synthesis is crucial for the accomplishment of seed germination (Lu et al. 2016, Qu et al. 2020, Zaynab et al. 2021). Here, we focused on mitochondrial and chloroplastic proteins in our study.

Twenty-six percent of proteins upregulated in Norway maple at the imbibed stage were chloroplastic and included chloroplast structural proteins (Table S4 available as Supplementary data at *Tree Physiology* Online). Long-lived orthodox-type seeds are assumed to contain dismantled thylakoids of chloroplasts (Ballesteros et al. 2020). In contrast, short-lived chlorophyllous seeds maintain well-developed photosystems in the dry state (Ballesteros et al. 2019, Simkin et al. 2020). In this context, dry Norway maple seeds appeared to have dismantled chloroplasts, whereas recalcitrant sycamore seeds contain well-defined plastids with atypical granal structures and possess the potential to fix CO_2_ (Pinfield et al. 1973). Additionally, at the germinated stage, Norway maple and sycamore differed in the regulation of chloroplastic proteins (Table S7 available as Supplementary data at *Tree Physiology* Online), suggesting that chloroplasts are more continuously active in sycamore.

Mitochondrial biogenesis is manifested in germinating orthodox seeds as the transition from static, low-density and quiescent promitochondria to well-structured and fully active and motile organelles (Czarna et al. 2016). Upregulation of mitochondrial Fe-S centers constituting the electron transport chain confirmed mitochondrial biogenesis in Norway maple at the imbibed stage (Law et al. 2014). In contrast, the sycamore mitochondria were preparing for division at the imbibed stage as suggested by the increased levels of mitochondrial fission protein 1 in sycamore (Table S4 available as Supplementary data at *Tree Physiology* Online) and the fact that fission of mature mitochondria occurs synergistically with mitotic cell division (Law et al. 2012). Following the stages of mitochondrial biogenesis (Paszkiewicz et al. 2017), imbibed Norway maple embryonic axes display a bioenergetic reactivation, which occurs immediately upon seed rehydration, whereas sycamore at the imbibed stage is one step further in the advancement of mitochondrial reactivation because given the enlarged pool of active mitochondria providing energy for germinative metabolism. Gibberellins promote the reactivation of mitochondrial dynamics during seed germination (Paszkiewicz et al. 2017), and upregulated GASA14 levels in sycamore might be involved in modulation of this process. Complexes of membrane and mitochondrial proteins, particularly membrane channels, were upregulated only in sycamore at the germinated stage. Thus, our -omic data support that the mitochondrial import pathway, a phenomenon of aerobically germinating embryos (Czarna et al. 2016), was significantly upregulated in sycamore.

### Proteins controlling the germination process under MetO redox control

PTMs are involved in the regulation of seed germination. For example, proteins involved in the ABA signalling pathway are phosphorylated and ubiquitinated (Yu et al. 2021). ROS are considered signalling molecules in germination (Bailly 2019), and oxidative modification of certain proteins might participate in modulating their activity (Cai and Yan 2013). Among oxidative PTMs, protein carbonylation and nitrosylation play a role in seed germination (Job et al. 2005, Tola et al. 2021, Zhang et al. 2023). Moreover, earlier studies indicated that seeds that fail to germinate often exhibit altered metabolism of sulfur amino acids (Gu et al. 2016). Therefore, we investigated whether Met oxidation is a mechanism for posttranslational control of seed germination. In particular, the role of proteins containing MetO in the regulation of seed germination is unknown in *Acer* seeds because imbibed and germinated seeds of Norway maple and sycamore differ in protein-bound MetO levels and the abundance of MetO reductase type B1 (MsrB1) and type B2 (MsrB2) (Wojciechowska et al. 2020*b*).

MetO is a PTM in proteins related to the regulation of protein activity (Rosenfeld et al. 2023). The literature contains evidence that cytoskeleton remodeling, seed protection (Dalling et al. 2020), fatty acid biosynthesis (de Boer et al. 1999), mobilization of stored lipids (Dolui et al. 2020), protein storage reserves (Baud et al. 2009) and metabolism reactivation (Carrera-Castaño et al. 2020, El-Maarouf-Bouteau 2022) are important for seed germination. Our study suggests that proteins involved in the above processes are under Met/MetO redox control. Dynamic changes in MetO levels in cytoskeletal proteins (Tables S9–S14 available as Supplementary data at *Tree Physiology* Online) indicate that cellular reorganization in germinating seeds might depend on Met/MetO redox switches. Functional analyses revealed that the cytoskeleton remodeling proteins were downregulated at MetO sites in sycamore seeds, which germinate earlier than Norway maple seeds (Alipour et al. 2021) and exhibit better seedling performance (Alipour et al. 2023). This feature might be related to cytoskeleton-dependent cell membrane recovery during seed germination (Tichá et al. 2020) and the fact that at the imbibed and germinated stages, sycamore seeds contain lower global MetO levels in proteins than Norway maple seeds (Alipour et al. 2021), as reflected by higher levels of MsrB2 (Wojciechowska et al. 2020*a*) and the significantly upregulated peptide MetO reductase B5 (A0A5C7IR33) reported in this study in sycamore at the germinated stage (Table S7 available as Supplementary data at *Tree Physiology* Online).

During seed germination, specific processes, including plant growth, seem to be differentially regulated by Met/MetO redox changes. For example, *SLEEPER* genes are essential for general plant development (Knip et al. 2012), whereas the STICHEL protein regulates branching in developing plants in a dose-dependent manner (Ilgenfritz et al. 2003). We propose that redox regulation at Met residues of these proteins in *Acer* germinating seeds oppositely affects the number of branches in the root system in seedlings, which are more branched in sycamore seedlings (Alipour et al. 2023). Peptidylprolyl isomerase (PPI) displayed similarity (86% identity) to the PPI subclass of cyclophilins, exhibiting a positive effect on germination and shoot and root growth (Kumari et al. 2015). In this context, decreasing MetO levels in PPI in sycamore might also contribute to improved seedling performance. DJ-1B is necessary for chloroplast development (Lin et al. 2011), and DJ-1B simultaneously functions as a holdase chaperone during oxidative stress (Lewandowska et al. 2019). Similarly, GASA14, which is involved in the regulation of leaf expansion, also modulates ROS accumulation (Pérez-Pérez et al. 2013, Sun et al. 2013). The above data suggest that growth-regulating proteins are associated with ROS control and are under Met/MetO redox control, confirming that oxidative processes in germinated seeds affect successful seedling establishment (Wawrzyniak et al. 2020). Studies of Arabidopsis *gasa14* null mutants revealed retarded germination and seedling establishment (Sun et al. 2013). Therefore, seedling performance might be under the Met/MetO redox control given that GASA14 contains dynamically regulated MetO site (|Log_2_FC| = 4.08) in sycamore and Norway maple at the germinated stage (Table S13 available as Supplementary data at *Tree Physiology* Online).

As suggested by the GO-based functional analyses, Met/MetO redox switches might also contribute to the metabolism of nitrogen compounds and possibly further the growth. In detail, the protein NRT1/PTR family 2.6-like, which displays decreased MetO levels in sycamore at the germinated stage, functions as a transporter involved in passive nitrate efflux (Aluko et al. 2023). Additionally, OEP16 regulates metabolic fluxes of amino acids and amines during the early stages of seed germination (Pudelski et al. 2012). Both the NRT1/PTR family 2.6-like and OEP16 contained increased MetO levels in Norway maple at the germinated stage; however, in sycamore at the germinated stage, MetO levels were significantly decreased in these proteins (Table S13 available as Supplementary data at *Tree Physiology* Online).

Among RPs, RPL10 and RPS26 are preferentially oxidized and then released by a chaperone and degraded (Yang et al. 2023). Aside from RPL10, eight RPs were reported to be dynamically regulated at Met residues at the germinated stage, whereas 13 RPs displayed Met redox changes at the imbibed stage (Tables S10 and S13 available as Supplementary data at *Tree Physiology* Online). To date, only one experimental study has confirmed that Met oxidation prevents the dimer formation and interaction of RPs L7/L12 with translation elongation factors (Shcherbik and Pestov 2019). The protein biosynthetic machinery involves translational initiation, elongation and release factors, and each class of members was identified as containing dynamically changing MetO levels (Tables S9–S14 available as Supplementary data at *Tree Physiology* Online), implicating that translation might be under Met/MetO redox control during seed germination.

## Conclusion

Proteome and metabolome analyses revealed contrasting regulation at the start and end of the germination process in Norway maple and sycamore embryonic axes. Both omics approaches revealed corresponding results underlying the action of metabolite interconversion enzymes and amino acid metabolism in the regulation of the imbibed and germinated stages in *Acer* species. We hypothesized that higher levels of the storage proteins considered as nitrogen sources would allow the faster completion of sycamore germination through the synthesis of metabolites related to growth and defense. The highly abundant amines in sycamore at the germinated stage confirmed this hypothesis and suggested that nitrogen utilization is also more efficient in sycamore. In particular, Put and Cad might contribute to greater postgerminative growth of sycamore. We suggest that the different dynamics of seed germination between Norway maple and sycamore might be modulated by certain metabolites and their abundance. For example, higher levels of HMG and hydroxylamine led to longer and faster germination processes in Norway maple and sycamore, respectively. This study suggests that different plant defense metabolites ensure endogenous protection in *Acer* species during germination. Sinigrin and Pip are postulated to enhance the quality of Norway maple seeds. In sycamore at the germinated stage, ABA/stress-induced proteins are assumed to contribute to better postgerminative growth. Additionally, organic plant growth stimulants (i.e. 4-aminobenzoic acid) were found to be significantly more abundant in sycamore, as were B1, B3, B6, B9 and B12 vitamins, which are known to regulate seed germination and seedling growth.

We provided evidence that germination-related proteins, particularly cytoskeletal proteins and proteins controlling plant growth, are under Met redox control. As suggested by the GO-based functional analyses, Met/MetO redox switches might also contribute to the activity of enzymes related to the metabolism of nitrogen compounds that were heavily oxidized at Met residues in Norway maple. The whole protein biosynthetic machinery appeared to be modulated by the Met redox state because many RPs, together with translation initiation and elongation factors, were oppositely regulated at MetO sites in Norway maple and sycamore. In addition, we presented proteomic evidence for chloroplast and mitochondrial biogenesis in Norway maple, suggesting that dry Norway maple seeds probably contained dismantled chloroplasts and promitochondia, whereas reactivation of these organelles was distinct in sycamore. Importantly, chloroplast biogenesis is postulated to be under Met/MetO redox control.

Climate change could preclude, delay or enhance seed germination but seems to be more detrimental to seedlings. The abundance of metabolites related to growth and defense is hypothesized to facilitate the survival of sycamore seedlings during winter drought. However, severe soil moisture deficits may cause desiccation and mortality of sycamore germinating seeds, whereas desiccation-tolerant Norway maple germinating seeds have a higher chance of survival. Additionally, efficient nitrogen utilization and better postgerminative growth might be beneficial for sycamore seedlings in competing with spreading invasive species.

## Supplementary Material

Figure_S1_tpaf003

Figure_S2_tpaf003

Table_S1_tpaf003

Table_S2_tpaf003

Table_S3_tpaf003

Table_S4_tpaf003

Table_S5_tpaf003

Table_S6_tpaf003

Table_S7_tpaf003

Table_S8_tpaf003

Table_S9_tpaf003

Table_S10_tpaf003

Table_S11_tpaf003

Table_S12_tpaf003

Table_S13_tpaf003

Table_S14_tpaf003

## Data Availability

The mass spectrometry proteomics data have been deposited to the ProteomeXchange Consortium via the PRIDE (Perez-Riverol et al. 2022) partner repository with the dataset identifier PXD058130 and https://doi.org/10.6019/PXD058130. Other data that support the findings of this study will be made available upon request.

## References

[ref1] Ahmed S, Lutz D, Rapp J, Huish R, Dufour B, Brunelle A, Morelli TL, Stinson K, Warne T (2023) Climate change and maple syrup: producer observations, perceptions, knowledge, and adaptation strategies. Front For Glob Chang 6:1092218. 10.3389/ffgc.2023.1092218.

[ref2] Alipour S, Bilska K, Stolarska E, Wojciechowska N, Kalemba EM (2021) Nicotinamide adenine dinucleotides are associated with distinct redox control of germination in *Acer* seeds with contrasting physiology. PloS One 16:e0245635. 10.1371/journal.pone.0245635. 33503034 PMC7840005

[ref3] Alipour S, Wojciechowska N, Bujarska-Borkowska B, Kalemba EM (2023) Distinct redox state regulation in the seedling performance of Norway maple and sycamore. J Plant Res 136:83–96. 10.1007/s10265-022-01419-3. 36385674 PMC9831958

[ref4] Aluko OO, Kant S, Adedire OM, Li C, Yuan G, Liu H, Wang Q (2023) Unlocking the potentials of nitrate transporters at improving plant nitrogen use efficiency. Front Plant Sci 14:1074839. 10.3389/fpls.2023.1074839. 36895876 PMC9989036

[ref5] Badano EI, de Oca EJS-M d (2022) Seed fate, seedling establishment and the role of propagule size in forest regeneration under climate change conditions. For Ecol Manage 503:119776. 10.1016/j.foreco.2021.119776.

[ref6] Bailly C (2019) The signalling role of ROS in the regulation of seed germination and dormancy. Biochem J 476:3019–3032. 10.1042/BCJ20190159. 31657442

[ref7] Ballesteros D, Hill LM, Lynch RT, Pritchard HW, Walters C (2019) Longevity of preserved germplasm: the temperature dependency of aging reactions in glassy matrices of dried fern spores. Plant Cell Physiol 60:376–392. 10.1093/pcp/pcy217. 30398653

[ref8] Ballesteros D, Pritchard HW, Walters C (2020) Dry architecture: towards the understanding of the variation of longevity in desiccation-tolerant germplasm. Seed Sci Res 30:142–155. 10.1017/S0960258520000239.

[ref9] Bartzatt R, Wol T (2014) Detection and assay of vitamin B-2 (riboflavin) in alkaline borate buffer with UV/visible spectrophotometry. Int Sch Res Notices 2014:453085. 10.1155/2014/453085. 27379273 PMC4897266

[ref10] Basu S, Groot SPC (2023) Seed vigour and invigoration. In: Dadlani M, Yadava DK (eds) Seed science and technology: biology, production, quality. Springer Nature, Singapore, pp 67–89.

[ref11] Baud S, Dichow NR, Kelemen Z et al. (2009) Regulation of HSD1 in seeds of *Arabidopsis thaliana*. Plant Cell Physiol 50:1463–1478. 10.1093/pcp/pcp092. 19542545

[ref12] Bewley JD, Black M (2013) Seeds: physiology of development and germination. Springer Science & Business Media, Springer New York.

[ref13] Bhagwat M, Aravind L (2007) PSI-BLAST tutorial. In: Comparative genomics: volumes 1 and 2. Humana Press, vol. 395, pp 177–186. 10.1007/978-1-59745-514-5_10. PMC4781153 17993673

[ref14] Bischoff KL (2021) Chapter 53 - Glucosinolates. In: Gupta RC, Lall R, Srivastava A (eds) Nutraceuticals (second edition). Academic Press, Cambridge, Massachusetts, pp 903–909.

[ref16] Boucelha L, Abrous-Belbachir O, Djebbar R (2021) Is protein carbonylation a biomarker of seed priming and ageing? Funct Plant Biol 48:611–623. 10.1071/FP21001. 33617758

[ref17] Cai Z, Yan L-J (2013) Protein oxidative modifications: beneficial roles in disease and health. J Biochem Pharmacol Res 1:15–26. 23662248 PMC3646577

[ref18] Carrera-Castaño G, Calleja-Cabrera J, Pernas M, Gómez L, Oñate-Sánchez L (2020) An updated overview on the regulation of seed germination. Plants (Basel) 9:703. 10.3390/plants9060703. 32492790 PMC7356954

[ref19] Castillejo MA, Pascual J, Jorrín-Novo JV, Balbuena TS (2023) Proteomics research in forest trees: a 2012-2022 update. Front Plant Sci 14:1130665. 10.3389/fpls.2023.1130665. 37089649 PMC10114611

[ref20] Caudullo G, de Rigo D (2016) *Acer platanoides* in Europe: distribution, habitat, usage and threats. In: San-Miguel-Ayanz J, de, Rigo D, Caudullo G, Houston Durrant T, Mauri A (eds) European atlas of forest tree species. Publ. Off. EU, Luxembourg, p e019159.

[ref21] Chakrabarty A, Banik N, Bhattacharjee S (2019) Redox-regulation of germination during imbibitional oxidative and chilling stress in an indica rice cultivar (*Oryza sativa* L., cultivar Ratna). Physiol Mol Biol Plants 25:649–665. 10.1007/s12298-019-00656-6. 31168230 PMC6522599

[ref22] Chen B-X, Fu H, Gao J-D, Zhang Y-X, Huang W-J, Chen Z-J, Qi-Zhang YS-J, Liu J (2022) Identification of Metabolomic biomarkers of seed vigor and aging in hybrid rice. Rice 15:7. 10.1186/s12284-022-00552-w. 35084595 PMC8795261

[ref23] Chen D, Shao Q, Yin L, Younis A, Zheng B (2018) Polyamine function in plants: metabolism, regulation on development, and roles in abiotic stress responses. Front Plant Sci 9:1945. 10.3389/fpls.2018.01945. 30687350 PMC6335389

[ref24] Chiva C, Olivella R, Borràs E, Espadas G, Pastor O, Solé A, Sabidó E (2018) QCloud: a cloud-based quality control system for mass spectrometry-based proteomics laboratories. PloS One 13:e0189209. 10.1371/journal.pone.0189209. 29324744 PMC5764250

[ref25] Czarna M, Kolodziejczak M, Janska H (2016) Mitochondrial proteome studies in seeds during germination. Proteomes 4:19. 10.3390/proteomes4020019. 28248229 PMC5217346

[ref26] Dalling JW, Davis AS, Arnold AE, Sarmiento C, Zalamea P-C (2020) Extending plant defense theory to seeds. Annu Rev Ecol Evol Syst 51:123–141. 10.1146/annurev-ecolsys-012120-115156.

[ref86] da Rosa MS, da Rosa-Junior NT, Parmeggiani B, Glänzel NM, de Moura AL, Ribeiro RT, Grings M, Wajner M, Leipnitz G (2020) 3-Hydroxy-3-methylglutaric acid impairs redox and energy homeostasis, mitochondrial dynamics, and endoplasmic reticulum-mitochondria crosstalk in rat brain. Neurotox Res 37:314–325. 10.1007/s12640-019-00122-x. 31721046

[ref15] de Boer G-J, Testerink C, Pielage G, Nijkamp HJJ, Stuitje AR (1999) Sequences surrounding the transcription initiation site of the Arabidopsis enoyl-acyl carrier protein reductase gene control seed expression in transgenic tobacco. Plant Mol Biol 39:1197–1207. 10.1023/A:1006129924683. 10380806

[ref27] Dickie JB, May K, Morris SVA, Titley SE (1991) The effects of desiccation on seed survival in *Acer platanoides* L. and *Acer pseudoplatanus* L. Seed Sci Res 3:149–162. 10.1017/S0960258500000829.

[ref28] Dolui AK, Latha M, Vijayaraj P (2020) *OsPLB* gene expressed during seed germination encodes a phospholipase in rice. 3 Biotech 10:30. 10.1007/s13205-019-2016-x. PMC6944725 32015947

[ref29] El-Maarouf-Bouteau H (2022) The seed and the metabolism regulation. Biology 11:168. 10.3390/biology11020168. 35205035 PMC8869448

[ref30] Erland LAE, Murch SJ, Reiter RJ, Saxena PK (2015) A new balancing act: the many roles of melatonin and serotonin in plant growth and development. Plant Signal Behav 10:e1096469. 10.1080/15592324.2015.1096469. 26418957 PMC4883872

[ref31] Gakière B, Hao J, de BL, Pétriacq P, Nunes-Nesi A, Fernie AR (2018) NAD^+^ biosynthesis and signaling in plants. Crit Rev Plant Sci 37:259–307. 10.1080/07352689.2018.1505591.

[ref32] Gallardo K, Job C, Groot SPC, Puype M, Demol H, Vandekerckhove J, Job D (2002) Importance of methionine biosynthesis for Arabidopsis seed germination and seedling growth. Physiol Plant 116:238–247. 10.1034/j.1399-3054.2002.1160214.x. 12354201

[ref33] González-Hernández AI, Scalschi L, Vicedo B, Marcos-Barbero EL, Morcuende R, Camañes G (2022) Putrescine: a key metabolite involved in plant development, tolerance and resistance responses to stress. Int J Mol Sci 23:2971. 10.3390/ijms23062971. 35328394 PMC8955586

[ref34] Gu J, Chao H, Gan L, Guo L, Zhang K, Li Y, Wang H, Raboanatahiry N, Li M (2016) Proteomic dissection of seed germination and seedling establishment in *Brassica napus*. Front Plant Sci 7:1482. 10.3389/fpls.2016.01482. 27822216 PMC5075573

[ref35] Guo H, Lyv Y, Zheng W et al. (2021) Comparative metabolomics reveals two metabolic modules affecting seed germination in rice (*Oryza sativa*). Metabolites 11:880. 10.3390/metabo11120880. 34940638 PMC8707830

[ref36] He D, Yang P (2013) Proteomics of rice seed germination. Front Plant Sci 4:246. 10.3389/fpls.2013.00246. 23847647 PMC3705172

[ref37] Hemmerlin A, Huchelmann A, Tritsch D, Schaller H, Bach TJ (2019) The specific molecular architecture of plant 3-hydroxy-3-methylglutaryl-CoA lyase. J Biol Chem 294:16186–16197. 10.1074/jbc.RA119.008839. 31515272 PMC6827278

[ref38] Hendricks SB, Taylorson RB (1974) Promotion of seed germination by nitrate, nitrite, hydroxylamine, and ammonium salts. Plant Physiol 54:304–309. 10.1104/pp.54.3.304. 16658878 PMC367401

[ref39] Hong TD, Ellis RH (1990) A comparison of maturation drying, germination, and desiccation tolerance between developing seeds of *Acer pseudoplatanus* L. and *Acer platanoides* L. New Phytol 116:589–596. 10.1111/j.1469-8137.1990.tb00543.x.

[ref40] Ibrahim AY, Ashour FH, Gadalla MA (2021) Exergy study of amine regeneration unit using diethanolamine in a refinery plant: a real start-up plant. Heliyon 7:e06241. 10.1016/j.heliyon.2021.e06241. 33665423 PMC7900684

[ref41] Ilgenfritz H, Bouyer D, Schnittger A, Mathur J, Kirik V, Schwab B, Chua N-H, Jürgens G, Hülskamp M (2003) The Arabidopsis *STICHEL* gene is a regulator of trichome branch number and encodes a novel protein. Plant Physiol 131:643–655. 10.1104/pp.014209. 12586888 PMC166840

[ref42] Jacques S, Ghesquière B, De Bock P-J, Demol H, Wahni K, Willems P, Messens J, Van Breusegem F, Gevaert K (2015) Protein methionine sulfoxide dynamics in *Arabidopsis thaliana* under oxidative stress. Mol Cell Proteomics 14:1217–1229. 10.1074/mcp.M114.043729. 25693801 PMC4424394

[ref43] Jancewicz AL, Gibbs NM, Masson PH (2016) Cadaverine’s functional role in plant development and environmental response. Front Plant Sci 7:870. 10.3389/fpls.2016.00870. 27446107 PMC4914950

[ref44] Job C, Rajjou L, Lovigny Y, Belghazi M, Job D (2005) Patterns of protein oxidation in Arabidopsis seeds and during germination. Plant Physiol 138:790–802. 10.1104/pp.105.062778. 15908592 PMC1150397

[ref46] Kalemba EM, Valot B, Job D, Bailly C, Meimoun P (2022) Are methionine sulfoxide-containing proteins related to seed longevity? A case study of *Arabidopsis thaliana* dry mature seeds using cyanogen bromide attack and two-dimensional-diagonal electrophoresis. Plants (Basel) 11:569. 10.3390/plants11040569. 35214905 PMC8875303

[ref45] Kalemba EM, Gevaert K, Impens F, Dufour S, Czerwoniec A (2024) The association of protein-bound methionine sulfoxide with proteomic basis for aging in beech seeds. BMC Plant Biol 24:377. 10.1186/s12870-024-05085-6. 38714916 PMC11077735

[ref47] Kim G, Weiss SJ, Levine RL (2014) Methionine oxidation and reduction in proteins. Biochim Biophys Acta 1840:901–905. 10.1016/j.bbagen.2013.04.038. 23648414 PMC3766491

[ref48] Knip M, de Pater S, Hooykaas PJJ (2012) The SLEEPER genes: a transposase-derived angiosperm-specific gene family. BMC Plant Biol 12:192. 10.1186/1471-2229-12-192. 23067104 PMC3499209

[ref49] Kumari S, Joshi R, Singh K, Roy S, Tripathi AK, Singh P, Singla-Pareek SL, Pareek A (2015) Expression of a cyclophilin OsCyp2-P isolated from a salt-tolerant landrace of rice in tobacco alleviates stress via ion homeostasis and limiting ROS accumulation. Funct Integr Genomics 15:395–412. 10.1007/s10142-014-0429-5. 25523384

[ref50] Lando AP, Viana WG, da Silva RA, Costa CDD, Fraga HPF, Santos M, Mioto PT, Guerra MP, Steiner N (2020) The physiological relationship between abscisic acid and gibberellin during seed germination of *Trichocline catharinensis* (Asteraceae) is associated with polyamine and antioxidant enzymes. J Plant Growth Regul 39:395–410. 10.1007/s00344-019-09990-1.

[ref51] Law SR, Narsai R, Taylor NL, Delannoy E, Carrie C, Giraud E, Millar AH, Small I, Whelan J (2012) Nucleotide and RNA metabolism prime translational initiation in the earliest events of mitochondrial biogenesis during Arabidopsis germination. Plant Physiol 158:1610–1627. 10.1104/pp.111.192351. 22345507 PMC3320173

[ref52] Law SR, Narsai R, Whelan J (2014) Mitochondrial biogenesis in plants during seed germination. Mitochondrion 19:214–221. 10.1016/j.mito.2014.04.002. 24727594

[ref53] Lewandowska A, Vo TN, Nguyen T-DH, Wahni K, Vertommen D, Van Breusegem F, Young D, Messens J (2019) Bifunctional chloroplastic DJ-1B from *Arabidopsis thaliana* is an oxidation-robust holdase and a glyoxalase sensitive to H₂O₂. Antioxidants (Basel) 8:8. 10.3390/antiox8010008. 30609642 PMC6356872

[ref54] Li HB, Chen F (2000) Determination of vitamin B12 in pharmaceutical preparations by a highly sensitive fluorimetric method. Fresenius J Anal Chem 368:836–838. 10.1007/s002160000595. 11227572

[ref55] Li J, Peng Z, Liu Y et al. (2022) Overexpression of peroxisome-localized GmABCA7 promotes seed germination in *Arabidopsis thaliana*. Int J Mol Sci 23:2389. 10.3390/ijms23042389. 35216505 PMC8879324

[ref56] Li Y, Yu Z, Jin J, Zhang Q, Wang G, Liu C, Wu J, Wang C, Liu X (2018) Impact of elevated CO_2_ on seed quality of soybean at the fresh edible and mature stages. Front Plant Sci 9:1413. 10.3389/fpls.2018.01413. 30386351 PMC6199416

[ref57] Lin J, Nazarenus TJ, Frey JL, Liang X, Wilson MA, Stone JM (2011) A plant DJ-1 homolog is essential for *Arabidopsis thaliana* chloroplast development. PloS One 6:e23731. 10.1371/journal.pone.0023731. 21886817 PMC3160306

[ref58] Lu X-J, Zhang X-L, Mei M, Liu G-L, Ma B-B (2016) Proteomic analysis of *Magnolia sieboldii* K. Koch seed germination. J Proteomics 133:76–85. 10.1016/j.jprot.2015.12.005. 26688106

[ref59] Mi H, Muruganujan A, Casagrande JT, Thomas PD (2013) Large-scale gene function analysis with the PANTHER classification system. Nat Protoc 8:1551–1566. 10.1038/nprot.2013.092. 23868073 PMC6519453

[ref60] Naegle ER, Burton JW, Carter TE, Rufty TW (2005) Influence of seed nitrogen content on seedling growth and recovery from nitrogen stress. Plant Soil 271:329–340. 10.1007/s11104-004-3242-4.

[ref61] Nahed G, Lobna S (2009) Some studies on the effect of putrescine, ascorbic acid and thiamine on growth, flowering and some chemical constituents of gladiolus plants at Nubaria. Ozean J Appl Sci 2:169–179. 10.3389/fpls.2018.01413.

[ref62] Naidoo S, Slippers B, Plett JM, Coles D, Oates CN (2019) The road to resistance in forest trees. Front Plant Sci 10:273. 10.3389/fpls.2019.00273. 31001287 PMC6455082

[ref63] Narula K, Sinha A, Haider T, Chakraborty N, Chakraborty S (2016) Seed proteomics: an overview. In: Salekdeh GH (ed) Agricultural proteomics Volume 1: Crops, horticulture, farm animals, food, insect and microorganisms. Springer International Publishing, Cham, pp 31–52.

[ref64] Neumann G, Azaizeh HA, Marschner H (1996) Thiamine (vitamin B1) seed treatment enhances germination and seedling growth of bean (*Phaseolus vulgaris* L.) exposed to soaking injury. J Plant Nutr Soil Sci 159:491–498. 10.1002/jpln.1996.3581590512.

[ref65] Ng PLL, Ferrarese MLL, Huber DA, Ravagnani ALS, Ferrarese-Filho O (2003) Canola (*Brassica napus* L.) seed germination influenced by cinnamic and benzoic acids and derivatives: effects on peroxidase. Seed Sci Tech 31:39–46. 10.15258/sst.2003.31.1.05.

[ref66] Nishimune T, Ito S, Abe M, Kimoto M, Hayashi R (1988) Conditions for thiamin assay by cyanogen bromide oxidation. J Nutr Sci Vitaminol (Tokyo) 34:543–552. 10.3177/jnsv.34.543. 3244042

[ref67] Osama SK, Kerr ED, Yousif AM, Phung TK, Kelly AM, Fox GP, Schulz BL (2021) Proteomics reveals commitment to germination in barley seeds is marked by loss of stress response proteins and mobilisation of nutrient reservoirs. J Proteomics 242:104221. 10.1016/j.jprot.2021.104221. 33866056

[ref68] Osuna D, Prieto P, Aguilar M (2015) Control of seed germination and plant development by carbon and nitrogen availability. Front Plant Sci 6:1023. 10.3389/fpls.2015.01023. 26635847 PMC4649081

[ref69] Pasta S, de Rigo D, Caudullo G (2016) *Acer pseudoplatanus* in Europe: distribution, habitat, usage and threats. In: San-Miguel Ayanz J, de, Rigo D, Caudullo G, Houston Durrant T, Mauri A (eds) European atlas of forest tree species. Publ. Off. EU, Luxembourg, p e01665a.

[ref70] Paszkiewicz G, Gualberto JM, Benamar A, Macherel D, Logan DC (2017) Arabidopsis seed mitochondria are bioenergetically active immediately upon imbibition and specialize via biogenesis in preparation for autotrophic growth. Plant Cell 29:109–128. 10.1105/tpc.16.00700. 28062752 PMC5304351

[ref71] Pawłowski TA (2009) Proteome analysis of Norway maple (*Acer platanoides* L.) seeds dormancy breaking and germination: influence of abscisic and gibberellic acids. BMC Plant Biol 9:48. 10.1186/1471-2229-9-48. 19413897 PMC2688491

[ref72] Pawłowski TA, Staszak AM (2016) Analysis of the embryo proteome of sycamore (*Acer pseudoplatanus* L.) seeds reveals a distinct class of proteins regulating dormancy release. J Plant Physiol 195:9–22. 10.1016/j.jplph.2016.02.017. 26970688

[ref73] Pérez-Pérez JM, Esteve-Bruna D, González-Bayón R, Kangasjärvi S, Caldana C, Hannah MA, Willmitzer L, Ponce MR, Micol JL (2013) Functional redundancy and divergence within the *Arabidopsis reticulata*-related gene family. Plant Physiol 162:589–603. 10.1104/pp.113.217323. 23596191 PMC3668055

[ref74] Perez-Riverol Y, Bai J, Bandla C et al. (2022) The PRIDE database resources in 2022: a hub for mass spectrometry-based proteomics evidences. Nucleic Acids Res 50:D543–D552. 10.1093/nar/gkab1038. 34723319 PMC8728295

[ref75] Pinfield NJ, Stobart AK, Crawford RM, Beckett A (1973) Carbon assimilation by sycamore cotyledons during early seedling development. J Exp Bot 24:1203–1205. 10.1093/jxb/24.6.1203.

[ref75a] Pudelski B, Schock A, Hoth S, Radchuk R, Weber H, Hofmann J, Sonnewald U, Soll J, Philippar K (2012) The plastid outer envelope protein OEP16 affects metabolic fluxes during ABA-controlled seed development and germination. J Exp Bot 63:1919–1936. 10.1093/jxb/err375. 22155670 PMC3295387

[ref76] Pri-Tal O, Sun Y, Dadras A et al. (2024) Constitutive activation of ABA receptors in Arabidopsis reveals unique regulatory circuitries. New Phytol 241:703–714. 10.1111/nph.19363. 37915144

[ref77] Qu C, Zhang S, Zhao H, Chen J, Zuo Z, Sun X, Cheng Y, Xu Z, Liu G (2020) Analysis of the energy source at the early stage of poplar seed germination: verification of Perl’s pathway. 3 Biotech 10:418. 10.1007/s13205-020-02413-z. PMC7474724 32953380

[ref78] Ramakrishna A, Giridhar P, Ravishankar GA (2011) Phytoserotonin. Plant Signal Behav 6:800–809. 10.4161/psb.6.6.15242. 21617371 PMC3218476

[ref79] Ramazi S, Zahiri J (2021) Post-translational modifications in proteins: resources, tools and prediction methods. Database (Oxford) 2021:baab012. 10.1093/database/baab012. 33826699 PMC8040245

[ref80] Reed RC, Bradford KJ, Khanday I (2022) Seed germination and vigor: ensuring crop sustainability in a changing climate. Heredity 128:450–459. 10.1038/s41437-022-00497-2. 35013549 PMC9177656

[ref81] Ritchie ME, Phipson B, Wu D, Hu Y, Law CW, Shi W, Smyth GK (2015) Limma powers differential expression analyses for RNA-sequencing and microarray studies. Nucleic Acids Res 43:e47. 10.1093/nar/gkv007. 25605792 PMC4402510

[ref82] Rodrigues AM, Miguel C, Chaves I, António C (2021) Mass spectrometry-based forest tree metabolomics. Mass Spectrom Rev 40:126–157. 10.1002/mas.21603. 31498921

[ref83] Rodrigues GAG, Mauve C, Gakiere B, Bailly C, Steiner N (2024) The metabolic profiles of *Eugenia astringens* and *E*. *uniflora* (Myrtaceae) sensitive seeds affect desiccation. Physiol Plant 176:e14220. 10.1111/ppl.14220. 38356368

[ref84] Roje S (2007) Vitamin B biosynthesis in plants. Phytochemistry 68:1904–1921. 10.1016/j.phytochem.2007.03.038. 17512961

[ref87] Rosenfeld MA, Yurina LV, Vasilyeva AD (2023) Antioxidant role of methionine-containing intra- and extracellular proteins. Biophys Rev 15:367–383. 10.1007/s12551-023-01056-7. 37396452 PMC10310685

[ref88] Schindeldecker M, Moosmann B (2015) Protein-borne methionine residues as structural antioxidants in mitochondria. Amino Acids 47:1421–1432. 10.1007/s00726-015-1955-8. 25859649

[ref89] Sharma P, Meyyazhagan A, Easwaran M et al. (2022) Hydrogen sulfide: a new warrior in assisting seed germination during adverse environmental conditions. Plant Growth Regul 98:401–420. 10.1007/s10725-022-00887-w.

[ref90] Shcherbik N, Pestov DG (2019) The impact of oxidative stress on ribosomes: from injury to regulation. Cells 8:1379. 10.3390/cells8111379. 31684095 PMC6912279

[ref91] Sheng S, Wu C, Xiang Y et al. (2022) Polyamine: a potent ameliorator for plant growth response and adaption to abiotic stresses particularly the ammonium stress antagonized by urea. Front Plant Sci 13:783597. 10.3389/fpls.2022.783597. 35401587 PMC8988247

[ref92] Simkin AJ, Faralli M, Ramamoorthy S, Lawson T (2020) Photosynthesis in non-foliar tissues: implications for yield. Plant J 101:1001–1015. 10.1111/tpj.14633. 31802560 PMC7064926

[ref93] Slaten ML, Yobi A, Bagaza C et al. (2020) mGWAS uncovers Gln-Glucosinolate seed-specific interaction and its role in metabolic homeostasis. Plant Physiol 183:483–500. 10.1104/pp.20.00039. 32317360 PMC7271782

[ref94] Staszak A, Pawłowski T (2012) Forest tree research in post genomic era. Introduction to systems biology of broadleaves. Dendrobiology 68:113–123. 10.3389/fpls.2022.783597.

[ref95] Staszak AM, Pawłowski TA (2014) Proteomic analysis of embryogenesis and the acquisition of seed dormancy in Norway maple (*Acer platanoides* L.). Int J Mol Sci 15:10868–10891. 10.3390/ijms150610868. 24941250 PMC4100186

[ref96] Stolarska E, Bilska K, Wojciechowska N, Bagniewska-Zadworna A, Rey P, Kalemba EM (2020) Integration of MsrB1 and MsrB2 in the redox network during the development of orthodox and recalcitrant *Acer* seeds. Antioxidants (Basel) 9:E1250. 10.3390/antiox9121250. PMC7763665 33316974

[ref97] Sumalan R-L, Croitor L, Petric M, Radulov I, Bourosh P, Sumalan R-M, Crisan M (2020) p-Aminobenzoate organic salts as potential plant growth regulators for tomatoes. Molecules 25:1635. 10.3390/molecules25071635. 32252303 PMC7180871

[ref98] Sun S, Wang H, Yu H, Zhong C, Zhang X, Peng J, Wang X (2013) GASA14 regulates leaf expansion and abiotic stress resistance by modulating reactive oxygen species accumulation. J Exp Bot 64:1637–1647. 10.1093/jxb/ert021. 23378382

[ref99] Suszka B, Muller C, Bonnet-Masimbert M, Gordon A (1996) Seeds of Forest broadleaves: from harvest to sowing. Institute National de la Recherche Agronomique, Paris.

[ref100] Tichá M, Richter H, Ovečka M, Maghelli N, Hrbáčková M, Dvořák P, Šamaj J, Šamajová O (2020) Advanced microscopy reveals complex developmental and subcellular localization patterns of ANNEXIN 1 in Arabidopsis. Front Plant Sci 11:1153. 10.3389/fpls.2020.01153. 32849711 PMC7419693

[ref101] Tola AJ, Jaballi A, Missihoun TD (2021) Protein carbonylation: emerging roles in plant redox biology and future prospects. Plan Theory 10:1451. 10.3390/plants10071451. PMC8309296 34371653

[ref102] Tyanova S, Temu T, Sinitcyn P, Carlson A, Hein MY, Geiger T, Mann M, Cox J (2016) The Perseus computational platform for comprehensive analysis of (prote)omics data. Nat Methods 13:731–740. 10.1038/nmeth.3901. 27348712

[ref103] UniProt Consortium (2021) UniProt: the universal protein knowledgebase in 2021. Nucleic Acids Res 49:D480–D489. 10.1093/nar/gkaa1100. 33237286 PMC7778908

[ref104] Vanderschuren H, Boycheva S, Li K-T, Szydlowski N, Gruissem W, Fitzpatrick TB (2013) Strategies for vitamin B6 biofortification of plants: a dual role as a micronutrient and a stress protectant. Front Plant Sci 4:143. 10.3389/fpls.2013.00143. 23734155 PMC3659326

[ref105] Wagner S, Bernhardt A, Leuendorf JE et al. (2006) Analysis of the Arabidopsis *rsr4-1/pdx1-3* mutant reveals the critical function of the PDX1 protein family in metabolism, development, and vitamin B6 biosynthesis. Plant Cell 18:1722–1735. 10.1105/tpc.105.036269. 16766694 PMC1488916

[ref106] Walters C (2015) Orthodoxy, recalcitrance and in-between: describing variation in seed storage characteristics using threshold responses to water loss. Planta 242:397–406. 10.1007/s00425-015-2312-6. 25985842

[ref107] Wang W-Q, Liu S-J, Song S-Q, Møller IM (2015) Proteomics of seed development, desiccation tolerance, germination and vigor. Plant Physiol Biochem 86:1–15. 10.1016/j.plaphy.2014.11.003. 25461695

[ref108] Waterworth WM, Latham R, Wang D, Alsharif M, West CE (2022) Seed DNA damage responses promote germination and growth in *Arabidopsis thaliana*. Proc Natl Acad Sci USA 119:e2202172119. 10.1073/pnas.2202172119. 35858436 PMC9335332

[ref109] Wawrzyniak MK, Kalemba EM, Ratajczak E, Chmielarz P (2020) Oxidation processes related to seed storage and seedling growth of *Malus sylvestris*, *Prunus avium* and *Prunus padus*. PloS One 15:e0234510. 10.1371/journal.pone.0234510. 32555619 PMC7302524

[ref110] Wojciechowska N, Alipour S, Stolarska E, Bilska K, Rey P, Kalemba EM (2020 *a*) Peptide-bound methionine sulfoxide (MetO) levels and MsrB2 abundance are differentially regulated during the desiccation phase in contrasted *Acer* seeds. Antioxidants (Basel) 9:E391. 10.3390/antiox9050391. PMC7278694 32392756

[ref111] Wojciechowska N, Alipour S, Stolarska E, Bilska K, Rey P, Kalemba EM (2020 *b*) Involvement of the MetO/Msr system in two *Acer* species that display contrasting characteristics during germination. Int J Mol Sci 21:9197. 10.3390/ijms21239197. 33276642 PMC7730483

[ref112] Xin Chi Y, Yang L, Jiang Zhao C, Muhammad I, Bo Zhou X, De Zhu H (2021) Effects of soaking seeds in exogenous vitamins on active oxygen metabolism and seedling growth under low-temperature stress. Saudi J Biol Sci 28:3254–3261. 10.1016/j.sjbs.2021.02.065. 34121863 PMC8176085

[ref113] Xue H, Zhang Q, Wang P et al. (2022) qPTMplants: an integrative database of quantitative post-translational modifications in plants. Nucleic Acids Res 50:D1491–D1499. 10.1093/nar/gkab945. 34718741 PMC8728288

[ref114] Yang M, Yang J, Su L, Sun K, Li D, Liu Y, Wang H, Chen Z, Guo T (2019) Metabolic profile analysis and identification of key metabolites during rice seed germination under low-temperature stress. Plant Sci 289:110282. 10.1016/j.plantsci.2019.110282. 31623771

[ref115] Yang Y-M, Jung Y, Abegg D, Adibekian A, Carroll KS, Karbstein K (2023) Chaperone-directed ribosome repair after oxidative damage. Mol Cell 83:1527–1537.e5. 10.1016/j.molcel.2023.03.030. 37086725 PMC10164075

[ref116] Yu F, Li M, He D, Yang P (2021) Advances on post-translational modifications involved in seed germination. Front Plant Sci 12:642979. 10.3389/fpls.2021.642979. 33828574 PMC8020409

[ref117] Zandoná LO, Lando AP, Goeten D, Steiner N (2021) The control over physiological dormancy break by gibberellins in *Calibrachoa sellowiana* (Sendtn.) Wijsman seeds are associated with polyamines. Acta Physiol Plant 43:160. 10.1007/s11738-021-03306-1.

[ref118] Zaynab M, Pan D, Fatima M, Sharif Y, Chen S, Chen W (2021) Proteomics analysis of *Cyclobalanopsis gilva* provides new insights of low seed germination. Biochimie 180:68–78. 10.1016/j.biochi.2020.10.008. 33250447

[ref119] Zeboon NH, Baqir HAA-R (2023) The effect of vitamin B9 and E on the yield and its components of the wheat crop. IOP Conf Ser Earth Environ Sci 1158:062033. 10.1088/1755-1315/1158/6/062033.

[ref120] Zhang H, He D, Yu J, Li M, Damaris RN, Gupta R, Kim ST, Yang P (2016) Analysis of dynamic protein carbonylation in rice embryo during germination through AP-SWATH. Proteomics 16:989–1000. 10.1002/pmic.201500248. 26801057

[ref121] Zhang T, Ayed C, Fisk ID, Pan T, Wang J, Yang N, Sun Q (2022) Evaluation of volatile metabolites as potential markers to predict naturally-aged seed vigour by coupling rapid analytical profiling techniques with chemometrics. Food Chem 367:130760. 10.1016/j.foodchem.2021.130760. 34390911

[ref122] Zhang X, Smits AH, van Tilburg GB, Ovaa H, Huber W, Vermeulen M (2018) Proteome-wide identification of ubiquitin interactions using UbIA-MS. Nat Protoc 13:530–550. 10.1038/nprot.2017.147. 29446774

[ref123] Zhang Y, Wang R, Wang X, Zhao C, Shen H, Yang L (2023) Nitric oxide regulates seed germination by integrating multiple signalling pathways. Int J Mol Sci 24:9052. 10.3390/ijms24109052. 37240398 PMC10219172

[ref124] Zhou M, Hassan MJ, Peng Y, Liu L, Liu W, Zhang Y, Li Z (2021) γ-Aminobutyric acid (GABA) priming improves seed germination and seedling stress tolerance associated with enhanced antioxidant metabolism, DREB expression, and dehydrin accumulation in white clover under water stress. Front Plant Sci 12:776939. 10.3389/fpls.2021.776939. 34925419 PMC8678086

