# Supplementary material for: Proteomics- and metabolomics-based analysis of the regulation of germination in Norway maple and sycamore embryonic axes
Source: Tree Physiol. 2025 Jan 6;45(2):tpaf003. doi: 10.1093/treephys/tpaf003 (PMC11791354; doi:10.1093/treephys/tpaf003)
Supplement: Figure_S1_tpaf003 [file figure_s1_tpaf003.pdf]

Figure S1. A principal component analysis was performed on the replicate samples using all quantified metabolites at imbibed (A) and germinated (B) stages and using all quantified proteins at imbibed (C) and germinated (D) stages, and using all quantified methionine oxidized peptides at imbibed (E) and germinated (F) stages as variables. The scatter plots show sample mappings along the two principal components (PCs). Percentages of explained data variance for each PC are shown on the x and y axis. APL – Norway maple, APS – sycamore, I – imbibed, G – germinated.

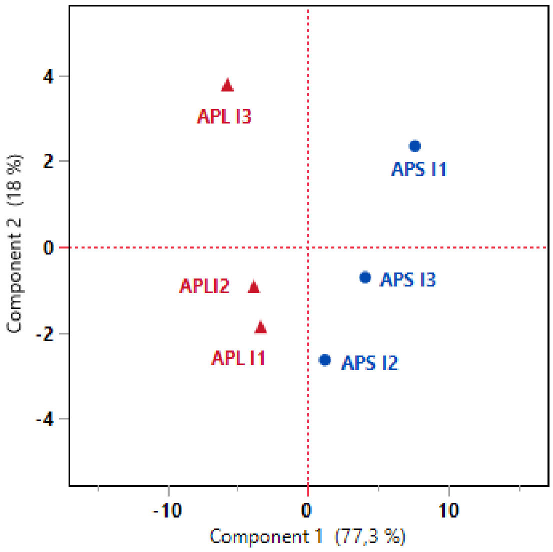

A)

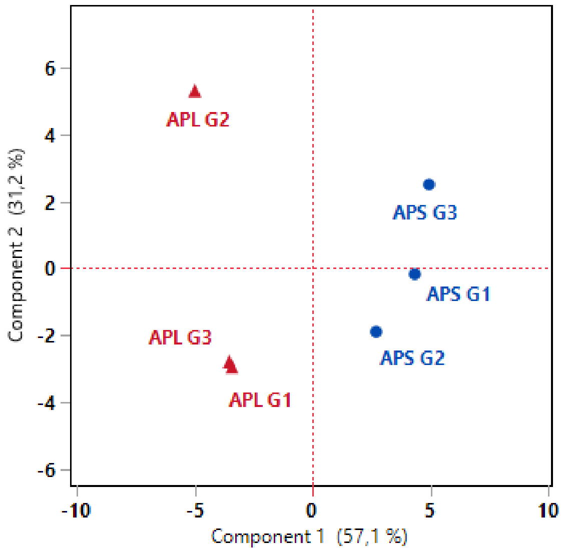

B)

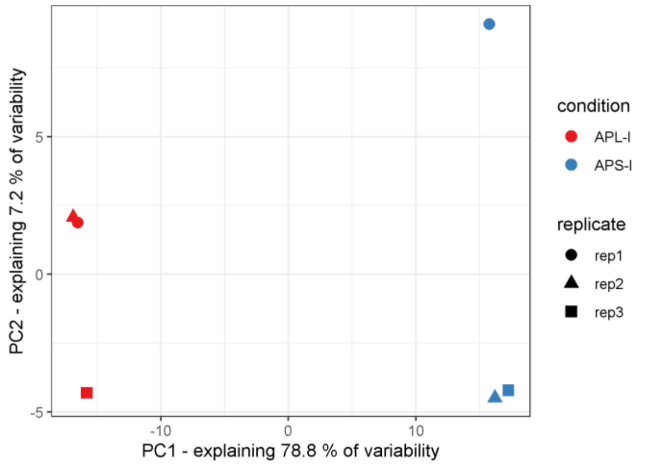

C)

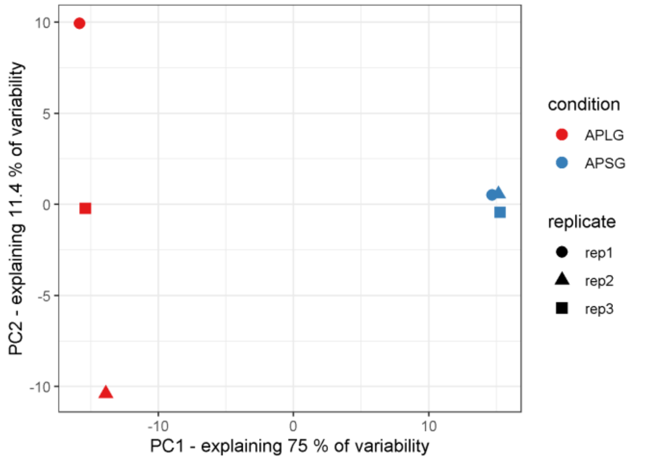

D)

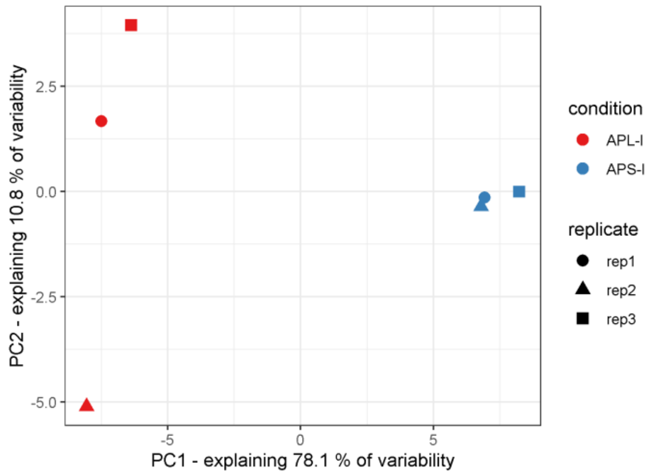

E)

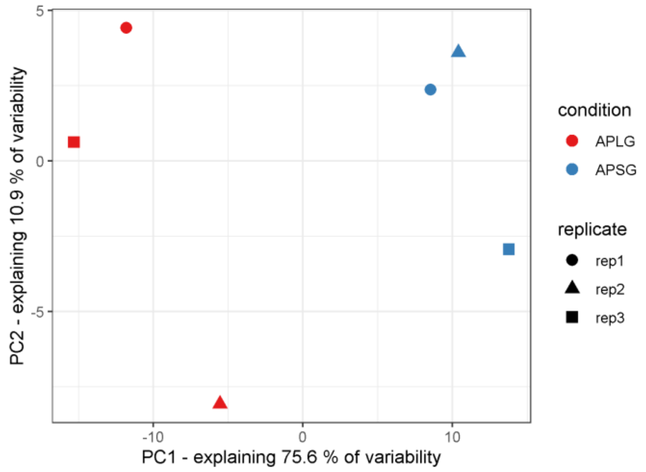

F)
