# Supplementary material for: Proteomics- and metabolomics-based analysis of the regulation of germination in Norway maple and sycamore embryonic axes
Source: Tree Physiol. 2025 Jan 6;45(2):tpaf003. doi: 10.1093/treephys/tpaf003 (PMC11791354; doi:10.1093/treephys/tpaf003)
Supplement: Table_S2_tpaf003 [file table_s2_tpaf003.docx]

**Table S2.** List of differentially abundant metabolites identified in embryonic axes of imbibed and germinated seeds of Norway maple and sycamore. Metabolites quantified as upregulated in sycamore are downregulated in Norway maple, and oppositely. The intensity value of each metabolite and statistical testing are found in Table S1.

| **IMBIBED STAGE** | |
| --- | --- |
| **Upregulated in sycamore** | **Downregulated in sycamore** |
| Diethanolamine | D-3-Phenyllactic acid |
| Histamine | 3-phenyl-2-propenoic acid |
| 4-Hydroxybenzoic acid | trans-Cinnamic acid |
| Pyridoxamine | Lyxose |
| 4-Aminobenzoic acid | L-Glutamic acid |
| 3-hydroxy-3-(4'-hydroxy-3'-methoxyphenyl)propionic acid | 1-Methylgalactose |
| 2-Methylpentanoic acid | Serotonin |
| N-Acetyl-D-glucosamine | Benzylalcohol |
| butyrolactam | 2,3-dihydroxybutanoic acid |
| p-Coummaric acid | Glycine |
| Putrescine | Hexitol |
| Niacinamide | Guanosine |
| L-Threonine |  |
| N-Methyl-DL-Alanine |  |
| L-Histidine |  |
| Succinic acid |  |
| Cadaverine |  |
| Pyruvic acid |  |
| Linolenic acid |  |
| DL-Threo-beta-Hydroxyaspartic acid |  |
| L-Prolinamide |  |
| DL-Isocitric acid |  |
| Inositol-4-monophosphate |  |
| 3-methyl-3-oxyglutaric acid |  |
| Citric acid |  |
| Sinigrin |  |
| L-(+)-Lactic acid |  |
| 16-Hydroxyhexadecanoic acid |  |
| Hydroxylamine |  |
| 2-tert-Mutylcyclohexanone |  |
| Urea |  |
| L-(-)-Malic acid |  |

| **GERMINATED STAGE** | |
| --- | --- |
| **Upregulated in sycamore** | **Downregulated in sycamore** |
| Diethanolamine | D-3-Phenyllactic acid |
| Histamine | DL-Pipecolic acid |
| 4-Hydroxybenzoic acid | 3-phenyl-2-propenoic acid |
| Pyridoxamine | trans-Cinnamic acid |
| 4-Aminobenzoic acid | Thymine |
| 3-hydroxy-3-(4'-hydroxy-3'-methoxyphenyl)propionic acid | Uracil |
| butyrolactam | Benzylmalonic acid methyl ester |
| p-Coummaric acid | L-Threonine |
| Niacinamide | GABA |
| L-Glutamic acid | Glycerol |
| erythronic acid lactone | Serotonin |
| L-Histidine | Xanthine |
| Tartaric acid | D-(+)-Galactose |
| Mannonic acid | Palmitic acid |
| 1-Methylgalactose | 3,6-Anhydro-D-galactose |
| Cadaverine | L-Valine |
| D-Panose | D-(+)-Fucose |
| 2-Deoxyerythritol | Argininosuccinate |
| 6-Deoxyglucitol | L-(+)-Lysine |
| L-Prolinamide | Hexitol |
| Malonic acid | Ribitol |
| Allantoin | Ethylmalonic acid |
| Maltotriose | 3,6-Anhydro-d-hexose |
| Glutathione | Fructose-1-phosphate |
| DL-Isocitric acid | D-Xylulose |
| 3-methyl-3-oxyglutaric acid | Glycerol-3-galactoside |
| Citramalic acid |  |
| Sinigrin |  |
| 16-Hydroxyhexadecanoic acid |  |
| 6-Deoxygalactofuranose |  |
| 3-Hydroxy-3-methylglutaric acid |  |
