# Supplementary material for: Proteomics- and metabolomics-based analysis of the regulation of germination in Norway maple and sycamore embryonic axes
Source: Tree Physiol. 2025 Jan 6;45(2):tpaf003. doi: 10.1093/treephys/tpaf003 (PMC11791354; doi:10.1093/treephys/tpaf003)
Supplement: Table_S4_tpaf003 [file table_s4_tpaf003.docx]

**Table S4.** List of proteins identified in our study with calculated changes in abundance assigned as upregulated (log_2_FC>2) and downregulated (log_2_FC<–2) in imbibed Norway maple and seeds as compared to imbibed sycamore seeds. Protein name was derived from the UniProt database (UniProt Consortium 2021) accessed in February 2024. * Gene abbreviation refers to homological *Arabidopsis thaliana* gene recognized by protein-coding gene classification information knowledgebases. **Amino acid sequences of proteins assigned as uncharacterized, containing a specific domain or identified only to class were extracted from UniProt database and were explicated using PSI-BLAST search method (Bhagwat and Aravind 2007). Green color font of a gene refers to the chloroplastic protein.

| **adj P Val** | **Log_2_FC** | **Majority protein IDs** | **Protein name** | **Explicated protein name**** | | **Gene*** | **Gene name** |
| --- | --- | --- | --- | --- | --- | --- | --- |
| 0.000064 | 8.28 | A0A3Q9D3H9 | Cytosolic glyceraldehyde-3-phosphate dehydrogenase |  | | At3g04120 |  |
| 0.00016 | 7.75 | A0A5C7IWE5 | Poly [ADP-ribose] polymerase |  | | At2g31320 | EZV62_002209 |
| 0.000064 | 7.42 | A0A5C7HS14 | Endoglucanase |  | | At1g71380 | EZV62_014353 |
| 0.000078 | 6.67 | A0A5C7GUD1 | GLTP domain-containing protein |  | | At3g27120 | EZV62_027383 |
| 0.00015 | 6.61 | A0A5C7IN74 | Oleosin |  | | At4g25140 | EZV62_005454 |
| 0.00076 | 6.44 | A0A5C7HEL7 | Uncharacterized protein | Cellulose synthase-like protein B1-related | | At2g32610 | EZV62_020653 |
| 0.000077 | 6.41 | A0A5C7I4X0 | Peptidase A1 domain-containing protein |  | | At5g22320 | EZV62_010610 |
| 0.00013 | 6.25 | A0A5C7H3Y3 | CCHC-type domain-containing protein | PLAT domain-containing protein 1 | | At4g39730 | EZV62_024151 |
| 0.000083 | 5.94 | A0A5C7H156 | Tubulin beta chain |  | | At5g23860 | EZV62_023019 |
| 0.000083 | 5.85 | A0A5C7IRP9 | SMP domain-containing protein |  | | At1g73200 | EZV62_000642 |
| 0.00015 | 5.81 | A0A5C7IVT0 | Rho-GAP domain-containing protein | Rho GTPase-activating protein 1 | | At5g22400 | EZV62_001989 |
| 0.00025 | 5.33 | A0A5C7HEY0 | Isocitrate lyase |  | | At3g21720 | EZV62_020604 |
| 0.00028 | 4.82 | A0A5C7HRM4 | Acetohydroxy-acid synthase small subunit |  | | At5g16290 | EZV62_014038 |
| 0.00018 | 4.79 | A0A5C7GQL9 | AB hydrolase-1 domain-containing protein | Methylesterase 17 | | At3g10870 | EZV62_026139 |
| 0.00018 | 4.66 | A0A5C7H403 | Bifunctional inhibitor/plant lipid transfer protein/seed storage helical domain-containing protein | Transcription factor TT8 | | At4g09820 | EZV62_023949 |
| 0.000077 | 4.52 | A0A5C7I1S9 | Beta-glucosidase |  | | At1g52400 | EZV62_010427 |
| 0.000083 | 4.51 | A0A5C7HEA8 | Aminotransferase class I/classII domain-containing protein |  | | At4g28410 | EZV62_020364 |
| 0.00015 | 4.5 | A0A5C7HTD2 | Enoyl reductase (ER) domain-containing protein | Cinnamyl alcohol dehydrogenase 2 | | At2g21730 | EZV62_014627 |
| 0.00054 | 4.48 | A0A5C7H4W7 | Acetolactate synthase |  | | At3g48560 | EZV62_024303 |
| 0.00019 | 4.4 | A0A5C7HU77 | Leucine-rich repeat-containing N-terminal plant-type domain-containing protein |  | | At1g35710 | EZV62_014325 |
| 0.00018 | 4.35 | A0A5C7ILI7 | SHSP domain-containing protein | 26.5 kDa heat shock protein, mitochondrial-like | | At1g52560 | EZV62_004271 |
| 0.000064 | 4.33 | A0A5C7GTD5; A0A5C7GT33; A0A5C7GTH8 | DNA helicase | DNA replication licensing factor MCM7 | | At4g02060 | EZV62_027177; EZV62_027182; EZV62_027179 |
| 0.000077 | 4.19 | A0A5C7GSH9 | Uncharacterized protein | THO complex subunit 4A-like | | At5g59950 | EZV62_026721 |
| 0.000078 | 4.17 | A0A5C7H4Y6 | Enoyl reductase (ER) domain-containing protein | Cinnamyl alcohol dehydrogenase 9 | | At4g39330 | EZV62_021180 |
| 0.002 | 3.87 | A0A5C7GZ98 | Uncharacterized protein | 3-oxoacyl-[acyl-carrier-protein] reductase | | At1g24360 | EZV62_025700 |
| 0.022 | 3.8 | A0A5C7HPQ0 | Pyridoxamine 5'-phosphate oxidase putative domain-containing protein |  | | At5g49970 | EZV62_016810 |
| 0.025 | 3.64 | A0A5C7IGD3; A0A5C7IGQ1; A0A5C7HUB9 | SGNH hydrolase-type esterase domain-containing protein | GDSL esterase/lipase | | At1g28580 | EZV62_003136; EZV62_003134; EZV62_014912 |
| 0.00019 | 3.63 | A0A5C7IXJ8 | 4-hydroxy-3-methylbut-2-en-1-yl diphosphate synthase |  | | At5g60600 | EZV62_002437 |
| 0.0005 | 3.56 | A0A5C7IUH1 | CBM20 domain-containing protein | Chloroplast alpha-glucan water dikinase | | At1g10760 | EZV62_001500 |
| 0.0011 | 3.56 | A0A5C7HK80 | Probable 6-phosphogluconolactonase |  | | At5g24410 | EZV62_017959 |
| 0.022 | 3.55 | A0A5C7IB73 | DM2 domain-containing protein | Upstream activation factor subunit spp27 | | At2g39240 | EZV62_007760 |
| 0.0046 | 3.5 | A0A5C7H5Q3; A0A5C7HIF4 | RNA helicase |  | | At1g20920 | EZV62_021208; EZV62_017947 |
| 0.00016 | 3.49 | A0A5C7HYA8 | Prephenate/arogenate dehydrogenase domain-containing protein |  | | At5g34930 | EZV62_013263 |
| 0.00027 | 3.48 | A0A5C7HSG4 | Ferredoxin--NADP reductase, chloroplastic |  | | At5g66190 | EZV62_014368 |
| 0.00017 | 3.34 | A0A5C7IR41 | Prefoldin subunit 3 |  | | At5g49510 | EZV62_000147 |
| 0.0035 | 3.32 | A0A5C7IT36; A0A5C7IV68 | 3-isopropylmalate dehydratase | Aconitase/3-isopropylmalate dehydratase large subunit alpha/beta/alpha domain-containing protein | | At2g43090 | EZV62_001010; EZV62_001011 |
| 0.0015 | 3.29 | A0A5C7H3X0 | Heparanase-like protein 1 |  | | At5g07830 | EZV62_024208 |
| 0.0043 | 3.17 | A0A5C7HPG8 | Non-specific lipid-transfer protein |  | | At1g27950 | EZV62_016792; EZV62_016793 |
| 0.00018 | 3.15 | A0A5C7IKP0 | 30S ribosomal protein S17, chloroplastic |  | | At1g79850 | EZV62_003951 |
| 0.001 | 3.15 | A0A5C7HBX7 | Uncharacterized protein | Regulator of nonsense transcripts 1 | | At5g47010 | EZV62_019625 |
| 0.00013 | 3.08 | A0A5C7H8B7 | Peroxidase | Peroxidase 12 | | At1g71695 | EZV62_022427 |
| 0.002 | 3.05 | A0A5C7HYD3 | DNA/RNA-binding protein Alba-like domain-containing protein |  | | At1g70450 | EZV62_013504; EZV62_007264 |
| 0.00043 | 3.04 | A0A5C7HYL5 | Uncharacterized protein | Alpha-aminoadipic semialdehyde synthase | | At4g33150 | EZV62_013112 |
| 0.00087 | 3.03 | A0A5C7H341 | Enoyl-CoA hydratase |  | | At4g16800 | EZV62_023723 |
| 0.000077 | 3.02 | A0A5C7IWK4 | 5-methyltetrahydropteroyltriglutamate--homocysteine S-methyltransferase |  | | At5g20980 | EZV62_002057 |
| 0.019 | 3.02 | A0A5C7I3S5 | Starch synthase, chloroplastic/amyloplastic |  | | At5g24300 | EZV62_010096; EZV62_023297 |
| 0.00016 | 3 | A0A5C7HT74 | Enoyl reductase (ER) domain-containing protein | Cinnamyl alcohol dehydrogenase 6 | | At4g37970 | EZV62_014631 |
| 0.0015 | 3 | A0A6B9M3X4; A0A7G7XUM8; A0A650G4C8 | ATP-dependent Clp protease proteolytic subunit |  | | At5g23140 | clpP; clpP ;clpP |
| 0.00023 | 2.99 | A0A5C7IAZ8; A0A5C7IBD0 | Polynucleotide phosphorylase 1 |  | | At3g03710 | EZV62_007690; EZV62_007686 |
| 0.0025 | 2.99 | A0A5C7IAM4 | DUF639 domain-containing protein | Argininosuccinate lyase, chloroplastic | | At5g10920 | EZV62_007665 |
| 0.00043 | 2.97 | A0A5C7GZT3 | Mannosyl-glycoprotein endo-beta-N-acetylglucosaminidase |  | | At5g05460 | EZV62_025926 |
| 0.04 | 2.97 | A0A5C7INT9 | Tubulin beta chain |  | | At1g64740 | EZV62_005845 |
| 0.00011 | 2.95 | A0A5C7HX63 | Elongation factor Ts, mitochondrial |  | | At4g11120 | EFTS |
| 0.00098 | 2.95 | A0A5C7HM11 | S1 motif domain-containing protein | rRNA biogenesis protein RRP5 | | At3g11964 | EZV62_015860 |
| 0.015 | 2.95 | A0A5C7HQB5 | Condensin complex subunit 1 C-terminal domain-containing protein |  | | At2g33540 | EZV62_016241 |
| 0.0021 | 2.94 | A0A5C7I6L5 | S-methyl-5-thioribose kinase |  | | At1g49820 | EZV62_011196 |
| 0.017 | 2.94 | A0A5C7J0K4; A0A5C7IYR8 | Glycosyltransferase | UDP-glycosyltransferase 71D1 | | At2g29730 | EZV62_002941; EZV62_002942 |
| 0.0028 | 2.9 | A0A5C7HYK7 | SHSP domain-containing protein | 17.6 kDa class II heat shock protein | | At5g12020 | EZV62_013211 |
| 0.00015 | 2.87 | A0A5C7IW76 | beta-glucosidase |  | | At5g20950 | EZV62_002062 |
| 0.0013 | 2.87 | A0A5C7I8F9; A0A5C7I9N2 | Cysteine synthase |  | | At2g43750 | EZV62_007128 |
| 0.012 | 2.84 | A0A5C7HZZ2 | Uncharacterized protein | Glucose/ribitol dehydrogenase | | At3g05260 | EZV62_009817 |
| 0.017 | 2.84 | A0A5C7HZL9 | RecA family profile 2 domain-containing protein |  | | At5g49220 | EZV62_012945 |
| 0.00069 | 2.83 | A0A5C7II35 | GDSL esterase/lipase APG |  | | At3g16370 | EZV62_003726 |
| 0.00028 | 2.8 | A0A5C7HCV0 | SHSP domain-containing protein | Small heat shock protein, chloroplastic-like | | At4g27670 | EZV62_020037 |
| 0.00059 | 2.8 | A0A5C7HJW9 | H15 domain-containing protein | Zinc finger CCCH domain-containing protein 15 | | At1g68200 | EZV62_018481 |
| 0.0015 | 2.79 | A0A5C7IGB3 | Adenylyltransferase and sulfurtransferase MOCS3 |  | | At5g55130 | MOCS3 |
| 0.0045 | 2.79 | A0A5C7HXS6 | Stress-response A/B barrel domain-containing protein | Stress-response A/B barrel domain-containing protein HS1 | | At3g17210 | EZV62_012997 |
| 0.00034 | 2.75 | A0A5C7HEE8 | ACB domain-containing protein | Acyl-CoA-binding protein | | At4g27780 | EZV62_019832 |
| 0.00087 | 2.75 | A0A5C7HMK9 | Enoyl-ACP reductase | Enoyl-[acyl-carrier-protein] reductase [NADH], chloroplastic | | At2g05990 | EZV62_015784 |
| 0.00013 | 2.74 | A0A5C7IKI1 | Microsomal glutathione S-transferase 3 |  | | At1g65820 | EZV62_004842 |
| 0.00011 | 2.73 | A0A5C7H8Z0 | Tubulin alpha chain |  | | At1g64740 | EZV62_021765; EZV62_022389 |
| 0.00028 | 2.72 | A0A5C7ITQ3 | Hydroxyethylthiazole kinase |  | | At3g25940 | EZV62_001078 |
| 0.00062 | 2.72 | A0A5C7IUY0 | Acetohydroxy-acid synthase small subunit | Acetolactate synthase small subunit 2, chloroplastic | | At5g16290 | EZV62_001574 |
| 0.00053 | 2.71 | A0A5C7H522 | Phosphotransferase | Hexokinase 1 | | At4g29130 | EZV62_021236 |
| 0.00013 | 2.69 | A0A5C7HP99 | Uncharacterized protein | Embryogenesis-like protein | | At1g71730 | EZV62_016660 |
| 0.00034 | 2.69 | A0A5C7H0A2 | Uncharacterized protein | RNA ligase/cyclic nucleotide phosphodiesterase | | At1g07910 | EZV62_022988 |
| 0.00056 | 2.69 | A0A5C7H427 | BolA protein | Protein BOLA1, chloroplastic | | At1g55805 | EZV62_024278 |
| 0.00015 | 2.68 | A0A5C7I2N2 | Uncharacterized protein | GATA transcription factor-like protein | | At3g24050 | EZV62_010339 |
| 0.00033 | 2.68 | A0A5C7IND2 | 3-oxoacyl-[acyl-carrier-protein] reductase |  | | At1g24360 | EZV62_005640 |
| 0.00021 | 2.66 | A0A5C7GUG1 | Ferredoxin--NADP reductase, chloroplastic |  | | At1g20020 | EZV62_027413 |
| 0.00016 | 2.63 | A0A5C7HPL5 | TatD related DNase |  | | At3g52390 | EZV62_015951 |
| 0.012 | 2.62 | A0A5C7HXI3;A0A5C7HZM3 | SHSP domain-containing protein | 17.7 kDa class II heat shock protein | | At5g12030 | EZV62_013204; EZV62_013209; EZV62_013194 |
| 0.00057 | 2.6 | A0A5C7IU61 | Protein farnesyltransferase/ geranylgeranyltransferase type-1 subunit alpha |  | | At3g59380 | EZV62_001430 |
| 0.001 | 2.6 | A0A5C7I875 | Amidase domain-containing protein | Amidase 1 | | At1g08980 | EZV62_006825 |
| 0.0013 | 2.6 | A0A5C7HE42 | NFU1 iron-sulfur cluster protein |  | | At4g01940 | EZV62_019732 |
| 0.0021 | 2.6 | A0A5C7IP03 | Tubulin beta chain |  | | At5g62690 | EZV62_005141 |
| 0.0023 | 2.58 | A0A5C7I1J7 | tRNA-binding domain-containing protein | aminoacyl tRNA synthase complex-interacting multifunctional protein 1 | | F24G16.250 | EZV62_010155 |
| 0.00014 | 2.57 | A0A5C7GRU6 | Uncharacterized protein | 3'-N-debenzoyl-2'-deoxytaxol N-benzoyltransferase | | At3g50270 | EZV62_026818 |
| 0.00053 | 2.57 | A0A5C7HLB6 | Ultraviolet-B receptor UVR8-like |  | | At4g34280 | EZV62_015646 |
| 0.0033 | 2.55 | A0A5C7IDK7 | Bet_v_1 domain-containing protein | Bet v I/Major latex protein domain-containing protein | | At5g28000 | EZV62_007801 |
| 0.00039 | 2.54 | A0A5C7HP12 | Inorganic diphosphatase |  | | At2g44570 | EZV62_016398 |
| 0.00058 | 2.53 | A0A5C7HML3 | U-box domain-containing protein | Probable ubiquitin conjugation factor E4 | | At5g15400 | EZV62_015441 |
| 0.00036 | 2.52 | A0A5C7H9F7 | Peptidase A1 domain-containing protein |  | | At3g46910 | EZV62_022351 |
| 0.01 | 2.52 | A0A5C7HWV1 | Aminotransferase class I/classII domain-containing protein | Bifunctional aspartate aminotransferase and glutamate/aspartate-prephenate aminotransferase | | At2g22250 | EZV62_015205 |
| 0.0011 | 2.51 | A0A5C7GVC3 | Pentacotripeptide-repeat region of PRORP domain-containing protein |  | | At1g60070 | EZV62_024528 |
| 0.011 | 2.51 | A0A5C7IEK3 | Uncharacterized protein | Cell division protein FtsZ | | At5g55280 | EZV62_008733 |
| 0.0048 | 2.49 | A0A5C7HCR7 | Uncharacterized protein | GDSL esterase/lipase | | At5g40990 | EZV62_019997 |
| 0.04 | 2.48 | A0A5C7HU22 | SAP domain-containing protein |  | | At4g39680 | EZV62_015186 |
| 0.0032 | 2.47 | A0A5C7IRW2 | Pyruvate kinase |  | | At3g06483 | EZV62_000726; EZV62_010731 |
| 0.0068 | 2.46 | A0A5C7IFB1 | beta-glucosidase | RNA-directed DNA polymerase | | At1g29940 | EZV62_008937; EZV62_002671 |
| 0.00016 | 2.45 | A0A5C7HVL3 | Glycosyltransferase |  | | At3g25140 | EZV62_014805 |
| 0.00023 | 2.45 | A0A5C7HIK9 | NADP-dependent oxidoreductase domain-containing protein |  | | At1g53050 | EZV62_017525; EZV62_018398; EZV62_017690 |
| 0.031 | 2.45 | A0A5C7GT35 | Uncharacterized protein | GDSL esterase/lipase At5g45670-like | | At5g45670- | EZV62_027039 |
| 0.049 | 2.43 | A0A5C7IJ74 | CobW C-terminal domain-containing protein |  | | At1g26520 | EZV62_004362 |
| 0.00031 | 2.42 | A0A5C7H802 | Phytocyanin domain-containing protein |  | | At5g07160 | EZV62_022253 |
| 0.00061 | 2.4 | A0A5C7IQL7 | glutamate--tRNA ligase |  | | At5g26710 | EZV62_006029; EZV62_006022 |
| 0.00011 | 2.39 | A0A5C7H0E7 | 1-deoxy-D-xylulose-5-phosphate reductoisomerase |  | | At5g62790 | EZV62_022801 |
| 0.00053 | 2.39 | A0A5C7I5P6 | WD_REPEATS_REGION domain-containing protein | WD repeat-containing protein 55 | | At2g34260 | EZV62_010846 |
| 0.0026 | 2.39 | A0A5C7I2F9 | ATP-grasp domain-containing protein |  | | At3g08840 | EZV62_010687 |
| 0.00089 | 2.38 | A0A5C7ID20 | Nucleolar complex protein 2 homolog |  | | At2g18220 | EZV62_008054 |
| 0.00027 | 2.34 | A0A5C7IL34; A0A5C7HBG4 | Ribose-phosphate diphosphokinase |  | | At2g35390 | EZV62_004795; EZV62_019600 |
| 0.016 | 2.32 | A0A650G7B1 | 50S ribosomal protein L20, chloroplastic |  | | AtCg00660 | rpl20 |
| 0.00088 | 2.31 | A0A5C7GWY3 | RING-type domain-containing protein | Protein NCA1 | | At3g54360 | EZV62_025139 |
| 0.0015 | 2.3 | A0A5C7HQZ6 | 2-isopropylmalate synthase |  | | At1g18500 | EZV62_014036 |
| 0.0072 | 2.3 | A0A5C7HYL2 | C3H1-type domain-containing protein |  | | At1g21570 | EZV62_013318 |
| 0.00042 | 2.28 | A0A5C7HWI4 | Methyltransferase domain-containing protein | |  | At4g29510 | EZV62_012673 |
| 0.0028 | 2.28 | A0A5C7I3L6 | Tim44 domain-containing protein |  | | At4g26370 | EZV62_010814 |
| 0.0039 | 2.28 | A0A5C7HWG4 | Uncharacterized protein | Protein LIKE COV 2 | | At1g43130 | EZV62_015055 |
| 0.0012 | 2.24 | A0A5C7IWT9 | Cyclin-dependent kinases regulatory subunit |  | | At2g27960 | EZV62_002339; EZV62_001198 |
| 0.03 | 2.24 | A0A5C7HPN3 | Tubulin/FtsZ GTPase domain-containing protein |  | | At2g36250 | EZV62_016105 |
| 0.00016 | 2.23 | A0A5C7IW86 | Structural maintenance of chromosomes protein |  | | At3g54670 | EZV62_002064 |
| 0.0013 | 2.23 | A0A5C7HNK6 | 1,4-alpha-glucan branching enzyme |  | | At2g36390 | EZV62_016154 |
| 0.0022 | 2.22 | A0A5C7H9N7 | WD_REPEATS_REGION domain-containing protein | Guanine nucleotide-binding protein, beta subunit | | At4g34460 | EZV62_022344 |
| 0.017 | 2.21 | A0A5C7HCB8 | NADP-dependent oxidoreductase domain-containing protein |  | | At1g53050 | EZV62_019775 |
| 0.0013 | 2.2 | A0A5C7GX23 | Porphobilinogen synthase |  | | At5g08280 | EZV62_024944; EZV62_024936 |
| 0.0057 | 2.19 | A0A5C7I1J2 | Peptidase C1A papain C-terminal domain-containing protein |  | | At1g13825 | EZV62_010151; EZV62_010152 |
| 0.021 | 2.19 | A0A5C7H2G0 | Trehalase |  | | At4g24040 | EZV62_023216 |
| 0.00019 | 2.18 | A0A5C7I1X7 | Pyruvate dehydrogenase E1 component subunit alpha |  | | At1g59900 | EZV62_010098; EZV62_023309 |
| 0.0017 | 2.16 | A0A5C7IY12 | SHSP domain-containing protein | HSP 22.0 kDa | | At4g10250 | EZV62_002734 |
| 0.0018 | 2.16 | A0A5C7I757 | Chitinase |  | | At3g12500 | EZV62_011979 |
| 0.0024 | 2.15 | A0A5C7HGB6 | Protein THYLAKOID FORMATION1, chloroplastic |  | | At2g20890 | EZV62_020512 |
| 0.0067 | 2.15 | A0A5C7GTI3 | CULLIN_2 domain-containing protein |  | | At4g03220 | EZV62_027227 |
| 0.037 | 2.15 | A0A067YPL1 | 30S ribosomal protein S8, chloroplastic |  | | AtCg00770 | rps8 |
| 0.0014 | 2.14 | A0A5C7IX65 | C3H1-type domain-containing protein | Zinc finger CCCH domain-containing protein 24-like | | At2g28450 | EZV62_002440 |
| 0.0067 | 2.12 | A0A5C7HA21 | Spindle assembly checkpoint component MAD1 |  | | At5g49880 | EZV62_018875 |
| 0.0015 | 2.11 | A0A5C7HMJ2 | leucine--tRNA ligase |  | | At4g04350 | EZV62_016070 |
| 0.0013 | 2.1 | A0A5C7GXM5 | Sulfotransferase |  | | At1g74090 | EZV62_025154 |
| 0.0025 | 2.1 | A0A5C7ILL4 | Pyridoxamine 5'-phosphate oxidase putative domain-containing protein |  | | At5g49970 | EZV62_004447 |
| 0.00018 | 2.08 | A0A5C7I1I4 | 3-hydroxyisobutyryl-CoA hydrolase |  | | At5g65940 | EZV62_013645 |
| 0.00054 | 2.08 | A0A5C7I8K1 | Mitochondrial Rho GTPase |  | | At5g27540 | EZV62_006800 |
| 0.021 | 2.07 | A0A5C7IVV1 | eIF-2B GDP-GTP exchange factor subunit gamma |  | | At5g19485 | EZV62_001962 |
| 0.0037 | 2.06 | A0A5C7IBN6; A0A5C7IBL1 | Cytochrome P450 |  | | At3g03470 | EZV62_007902; EZV62_007900 |
| 0.032 | 2.06 | A0A5C7HCD4 | Protease Do-like PDZ domain-containing protein |  | | At3g27925 | EZV62_019880; EZV62_022841 |
| 0.0097 | 2.05 | A0A5C7HXL5 | Serine aminopeptidase S33 domain-containing protein |  | | At3g29190 | EZV62_012941 |
| 0.003 | 2.04 | A0A5C7HY36 | SEC7 domain-containing protein |  | | At3g55230 | EZV62_013118 |
| 0.022 | 2.04 | A0A5C7H742 | Phospho-2-dehydro-3-deoxyheptonate aldolase |  | | At4g39980 | EZV62_021926 |
| 0.00069 | 2.03 | A0A5C7I2J3; A0A5C7IYJ1; A0A5C7IYK8 | Nucleolar protein 16; Protein kinase domain-containing protein |  | | At1g02060 | EZV62_010435; EZV62_002914; EZV62_002899 |
| 0.0016 | 2.03 | A0A5C7GWA7 | Glutamine--fructose-6-phosphate transaminase (isomerizing) |  | | At3g24090 | EZV62_024694 |
| 0.00033 | 2.02 | A0A5C7I2P3 | FCP1 homology domain-containing protein |  | | At2g36540 | EZV62_009890 |
| 0.0094 | 2.02 | A0A5C7IEP9 | Phytocyanin domain-containing protein |  | | At5g07160 | EZV62_008171 |
| 0.045 | 2.02 | A0A5C7GXW3 | Nfu/NifU N-terminal domain-containing protein |  | | At4g01940 | EZV62_025212 |

| **adj P Val** | **Log_2_FC** | **Majority protein IDs** | **Protein name** | **Explicated protein name**** | **Gene*** | **Gene name*** |
| --- | --- | --- | --- | --- | --- | --- |
| 0.045 | -2.02 | A0A5C7H8I5 | Variable large protein | D-3-phosphoglycerate dehydrogenase | At4g34200 | EZV62_021635 |
| 0.19 | -2.03 | A0A5C7IPK8 | Matrin-type domain-containing protein |  | At2g32600 | EZV62_006156 |
| 0.00033 | -2.04 | A0A5C7IME8 | Mitochondrial fission 1 protein |  | At3g57090 | EZV62_005193 |
| 0.000078 | -2.05 | A0A5C7IMI0 | EF-hand domain-containing protein | Probable calcium-binding protein CML48 | At2g27480 | EZV62_005233 |
| 0.0004 | -2.06 | A0A5C7IXY0; A0A5C7IY42 | SHSP domain-containing protein | HSP20-like chaperones | At5g20970 | EZV62_002622; EZV62_002627 |
| 0.012 | -2.07 | A0A5C7IL82 | Hikeshi-like domain-containing protein |  | At1g66080 | EZV62_004845 |
| 0.11 | -2.07 | A0A5C7HKB5 | BRO1 domain-containing protein |  |  | EZV62_018572 |
| 0.00023 | -2.09 | A0A5C7HHL6 | Bet v I/Major latex protein domain-containing protein | MLP-like protein 423 | At1g24020 | EZV62_017662 |
| 0.0032 | -2.09 | A0A5C7H1Z7 | Enoyl reductase (ER) domain-containing protein | Cinnamyl-alcohol dehydrogenase isoform 1 | At4g37980 | EZV62_023333 |
| 0.013 | -2.1 | A0A5C7HJX4 | Glycosyltransferase | UDP-glycosyltransferase 73C7 | At3g53160 | EZV62_018412 |
| 0.0025 | -2.12 | A0A5C7I8D2; A0A5C7HNV2 | Aquaporin |  | At4g35100 | EZV62_011846; EZV62_016338 |
| 0.057 | -2.15 | A0A5C7IUK7 | Uncharacterized protein |  |  | EZV62_000715 |
| 0.00027 | -2.16 | A0A5C7IHX7; A0A5C7IHD4 | Peptidase M20 dimerisation domain-containing protein |  | At4g20960 | EZV62_003620; EZV62_003635 |
| 0.0024 | -2.17 | A0A5C7GRW5 | Trans-cinnamate 4-monooxygenase |  | At2g30490 | EZV62_026210 |
| 0.00033 | -2.18 | A0A5C7HNY1 | Glutathione transferase |  | At3g09270 | EZV62_016540 |
| 0.038 | -2.19 | A0A5C7IC74 | EF-hand domain-containing protein | Caleosin | At5g29560 | EZV62_007331 |
| 0.003 | -2.21 | A0A5C7GQG9; A0A5C7GQ15; A0A5C7GRH4 | Cytochrome P450 | 71B11 type | At5g25120 | EZV62_026192; EZV62_026195; EZV62_026206 |
| 0.005 | -2.23 | A0A5C7HFZ9 | Xanthoxin dehydrogenase |  | At1g52340 | EZV62_017237 |
| 0.017 | -2.25 | A0A5C7IRW0 | Peptidase A1 domain-containing protein | Basic 7S globulin | At4g36700 | EZV62_000534 |
| 0.00039 | -2.27 | A0A5C7I8C4; A0A5C7I8D7 | Flavin-containing monooxygenase |  | At1g65860 | EZV62_012003; EZV62_012013 |
| 0.0013 | -2.27 | A0A5C7IWG6 | Homoserine dehydrogenase |  | At5g21060 | EZV62_002008 |
| 0.00057 | -2.31 | A0A5C7H4P3 | Hyaluronan/mRNA-binding protein domain-containing protein |  | At4g19830 | EZV62_023730 |
| 0.0092 | -2.32 | A0A5C7HU10 | Serine carboxypeptidase-like 18 |  | At1g33540 | EZV62_014898 |
| 0.0097 | -2.33 | A0A5C7GSR5 | Uncharacterized protein | Cell wall integrity and stress response component 4 like | At4g39840 | EZV62_027095 |
| 0.095 | -2.33 | A0A5C7GSB4 | Plug_translocon domain-containing protein |  |  | EZV62_026955 |
| 0.0044 | -2.34 | A0A5C7HZD5 | Pectinesterase |  | At1g53840 | EZV62_013109 |
| 0.0023 | -2.36 | A0A5C7I7M6 | ATP-dependent Clp protease proteolytic subunit |  | At5g23140 | EZV62_006615 |
| 0.037 | -2.36 | A0A5C7HBK7 | Uncharacterized protein | Abscisic acid receptor PYL1 | At5g46790 | EZV62_019563 |
| 0.0013 | -2.4 | A0A5C7IAS2; A0A5C7IB28 | NADH-cytochrome b5 reductase |  | At5g17770 | EZV62_007715; EZV62_007720 |
| 0.0013 | -2.42 | A0A5C7IXA6 | Lipoxygenase | LOX2, chloroplastic | At3g45140 | EZV62_001761; EZV62_001758 |
| 0.00029 | -2.45 | A0A5C7H661 | Cysteine protease |  | At4g35350 | EZV62_021629 |
| 0.0024 | -2.45 | A0A5C7IGH6 | Peptidase A1 domain-containing protein | Aspartyl protease family protein At5g10770-like | At5g10770 | EZV62_008831 |
| 0.001 | -2.47 | A0A5C7GUZ5 | Caffeic acid O-methyltransferase |  | At4g34050 | EZV62_024373 |
| 0.00039 | -2.48 | A0A5C7IV69 | Enoyl reductase (ER) domain-containing protein | 2-alkenal reductase | At5g16970 | EZV62_001568 |
| 0.000077 | -2.49 | A0A5C7HFB4 | Bet v I/Major latex protein domain-containing protein | MLP-like protein 34 | At1g70850 | EZV62_020408 |
| 0.019 | -2.49 | A0A5C7HW66 | HTH cro/C1-type domain-containing protein | Multiprotein-bridging factor 1c | At3g24500 | EZV62_012734 |
| 0.0012 | -2.5 | A0A5C7J092 | Uncharacterized protein | Zinc finger CCCH domain-containing protein 49-like | At4g29190 | EZV62_002831 |
| 0.00013 | -2.55 | A0A5C7IXZ3 | Uncharacterized protein | 22.0 kDa class IV heat shock protein | At4g10250 | EZV62_002730 |
| 0.0009 | -2.55 | A0A5C7IS67 | Amine oxidase | Copper amine oxidase | At1g62810 | EZV62_000624 |
| 0.24 | -2.56 | A0A5C7H9C8 | Dimer_Tnp_hAT domain-containing protein |  |  | EZV62_022254 |
| 0.077 | -2.57 | A0A5C7I961; A0A5C7I390; A0A5C7I220 | Histone H2A |  |  | EZV62_006959; EZV62_010548; EZV62_010547 |
| 0.00064 | -2.59 | A0A5C7I8B4 | Protein DETOXIFICATION |  | At5g52050 | EZV62_011993 |
| 0.00037 | -2.6 | A0A5C7IUZ0 | RNA helicase |  | At1g59760 | EZV62_001662; EZV62_012659; EZV62_015847 |
| 0.00016 | -2.61 | A0A5C7GRE1 | Allantoinase |  | At4g04955 | EZV62_026668 |
| 0.00061 | -2.61 | A0A5C7H548 | NAD-dependent epimerase/dehydratase domain-containing protein |  | At4g30470 | EZV62_021028 |
| 0.033 | -2.61 | A0A5C7HXA2 | Uncharacterized protein | Protein FATTY ACID EXPORT 2, chloroplastic | At3g43520 | EZV62_013124 |
| 0.0027 | -2.64 | A0A5C7HBQ2 | 4-hydroxyphenylpyruvate dioxygenase |  | At1g06570 | EZV62_019789 |
| 0.028 | -2.64 | A0A5C7GXL1 | Small nuclear ribonucleoprotein E |  | At2g18740 | EZV62_025353 |
| 0.002 | -2.67 | A0A5C7H4B5 | Enoyl reductase (ER) domain-containing protein |  | At5g43940 | EZV62_024301 |
| 0.00018 | -2.74 | A0A5C7GXN6 | Nudix hydrolase domain-containing protein | Nudix hydrolase 26, chloroplastic | At3g10620 | EZV62_025164; EZV62_015975 |
| 0.00044 | -2.74 | A0A5C7HYA5; A0A5C7I008 | YqgFc domain-containing protein |  | At3g52905 | EZV62_013486; EZV62_013677; EZV62_003176; EZV62_003097 |
| 0.013 | -2.77 | A0A5C7IVU9; A0A5C7ISU3; A0A5C7IU02 | RRM domain-containing protein | Glycine-rich RNA-binding protein 2, mitochondrial | At4g13850 | EZV62_001221; EZV62_000483; EZV62_000481 |
| 0.000077 | -2.79 | A0A5C7H0R5 | SHSP domain-containing protein | 18.1 kDa class I heat shock protein | At5g59720 | EZV62_025751 |
| 0.0019 | -2.92 | A0A5C7I519 | acetyl-CoA C-acyltransferase |  | At5g48230 | EZV62_010963 |
| 0.00078 | -2.93 | A0A5C7H725 | Expansin |  | At2g03090 | EZV62_021175 |
| 0.046 | -2.93 | A0A5C7HH16 | Glycosyltransferase |  | At1g05680 | EZV62_020918 |
| 0.00019 | -2.94 | A0A5C7IV29 | Lipoxygenase |  | At1g17420 | EZV62_001766 |
| 0.00014 | -2.95 | A0A5C7HD20 | Glutamate dehydrogenase |  | At5g18170 | EZV62_019751; EZV62_023753 |
| 0.00048 | -2.96 | A0A5C7GZW1 | ZZ-type domain-containing protein | Protein NBR1 homolog | At4g24690 | EZV62_022727 |
| 0.0052 | -2.97 | A0A5C7HP98 | RRM domain-containing protein | THO complex subunit 4A | At5g59950 | EZV62_016414 |
| 0.00054 | -2.98 | A0A5C7H369 | Annexin |  | At1g68090 | EZV62_023446 |
| 0.021 | -3 | A0A5C7HEU4 | DUF1421 domain-containing protein |  | At4g28300 | EZV62_020411 |
| 0.0028 | -3.01 | A0A5C7IWV4 | Enoyl reductase (ER) domain-containing protein | NADP-dependent alkenal double bond reductase P2 | At5g16990 | EZV62_001565 |
| 0.00059 | -3.07 | A0A5C7GZE5 | Chalcone-flavonone isomerase family protein |  | At3g55120 | EZV62_025846 |
| 0.012 | -3.11 | A0A5C7H0N1 | 4-coumarate--CoA ligase |  | At1g62940 | EZV62_023017 |
| 0.025 | -3.16 | A0A5C7GR32 | RRM domain-containing protein | Polyadenylate-binding RBP45B-like protein | At1g11650 | EZV62_026347 |
| 0.00044 | -3.17 | A0A5C7IEZ8; A0A5C7IEZ2 | Cytochrome P450 | Cytochrome P450 71D9 | At2g30770 | EZV62_008966; EZV62_009192 |
| 0.000076 | -3.26 | A0A5C7IL80; A0A5C7ILD2 | (+)-neomenthol dehydrogenase |  | At3g61220 | EZV62_004880; EZV62_004946 |
| 0.00077 | -3.28 | A0A5C7IHC0; A0A5C7IGK9; A0A5C7IGJ9; A0A5C7IH31 | Aminopeptidase | Aminopeptidase M1 | At4g33090 | EZV62_003390; EZV62_003392; EZV62_003382; EZV62_003386 |
| 0.00056 | -3.3 | A0A5C7I5G4 | Alpha/beta hydrolase fold-3 domain-containing protein | Probable carboxylesterase 12 | At3g48690 | EZV62_011338 |
| 0.00092 | -3.31 | A0A5C7HZL1 | Peptidase A1 domain-containing protein |  | At3g48090 | EZV62_013668 |
| 0.00085 | -3.34 | A0A5C7I284 | U6 snRNA-associated Sm-like protein LSm4 |  | At5g27720 | LSM4 |
| 0.00019 | -3.4 | A0A5C7HAH8; A0A5C7H9C2 | YqgFc domain-containing protein |  | At1g63210 | EZV62_018818; EZV62_018823 |
| 0.00028 | -3.46 | A0A5C7ISW7; A0A5C7IUY5 | Fe2OG dioxygenase domain-containing protein |  | At1g22950 | EZV62_000854; EZV62_000855 |
| 0.00034 | -3.47 | A0A5C7ISX0 | Inosine triphosphate pyrophosphatase |  | At4g13720 | EZV62_001092 |
| 0.00077 | -3.48 | A0A5C7HK19 | Bet_v_1 domain-containing protein | MLP-like protein 328 | At2g01520 | EZV62_018448 |
| 0.00021 | -3.52 | A0A5C7IGK8; A0A5C7IEQ2; A0A5C7IEX6; A0A5C7IE63 | Germin-like protein |  | At1g72610 | EZV62_008861; EZV62_008866; EZV62_008863; EZV62_008865 |
| 0.00031 | -3.66 | A0A5C7HSX1 | Chitinase |  | At4g19810 | EZV62_014458 |
| 0.00018 | -3.67 | A0A5C7HJG2 | Bet_v_1 domain-containing protein | MLP-like protein 28 | At1g70830 | EZV62_018464 |
| 0.00018 | -3.77 | A0A5C7H1W7 | 4-coumarate--CoA ligase |  | At5g63380 | EZV62_023016; EZV62_005846 |
| 0.0039 | -3.77 | A0A5C7IXN2 | RRM domain-containing protein |  | At5g59390 | EZV62_002594 |
| 0.00027 | -3.81 | A0A5C7HMU8 | Uncharacterized protein | Hyaluronan/mRNA-binding protein domain-containing protein | At4g19830 | EZV62_016182 |
| 0.00019 | -3.82 | A0A5C7HR47 | AB hydrolase-1 domain-containing protein | Salicylic acid-binding protein 2-like | AT2G23550 | EZV62_014090 |
| 0.000044 | -3.92 | A0A5C7ILD7 | Glutamate decarboxylase |  | At1g65960 | EZV62_004834 |
| 0.00048 | -3.94 | A0A5C7ITV1 | Acyl carrier protein |  | At1g54630 | EZV62_000385 |
| 0.00016 | -3.96 | A0A5C7IT28; A0A5C7IUQ9; A0A5C7ISG7 | Fe2OG dioxygenase domain-containing protein | 1-aminocyclopropane-1-carboxylate oxidase homolog | At5g43450 | EZV62_000857; EZV62_000851; EZV62_000849 |
| 0.00029 | -4.07 | A0A5C7J0Z2 | Saposin A-type domain-containing protein | Gamma-interferon-inducible lysosomal thiol reductase | At4g12960 | EZV62_003011 |
| 0.0025 | -4.15 | A0A5C7IBE6 | UDP-glycosyltransferases domain-containing protein | UDP-glycosyltransferase 87A1 | At2g30150 | EZV62_007739 |
| 0.00068 | -4.17 | A0A5C7HCH4 | Glycoside hydrolase family 31 N-terminal domain-containing protein |  | At5g11720 | EZV62_019448 |
| 0.00027 | -4.2 | A0A5C7I3X3 | Uncharacterized protein | Chaperonin CPN60-2, mitochondrial | At3g13470 | EZV62_010935; EZV62_015633 |
| 0.00024 | -4.27 | A0A5C7IQN5 | AB hydrolase-1 domain-containing protein | Epoxide hydrolase 1 | At5g13520 | EZV62_000272 |
| 0.00016 | -4.31 | A0A5C7H9W3 | DJ-1/PfpI domain-containing protein | protein DJ-1 homolog D | At3g02720 | EZV62_019249 |
| 0.0032 | -4.41 | A0A5C7IPQ6 | Reticulon-like protein |  | At4g23630 | EZV62_005974 |
| 0.00021 | -4.51 | A0A5C7IHG2 | Acid phosphatase |  | At4g26080 | EZV62_003668 |
| 0.000078 | -4.76 | A0A5C7IAE2 | Glycosyltransferase | UDP-glycosyltransferase 87A2 | At2g30140 | EZV62_007738 |
| 0.000044 | -4.78 | A0A5C7IV03 | DUF1264 domain-containing protein | Oil body-associated protein 1A | At1g05510 | EZV62_001498 |
| 0.0011 | -4.92 | A0A5C7IWC6 | Lipoxygenase | Linoleate 13S-lipoxygenase 2-1, chloroplastic | At1g67560 | EZV62_001763 |
| 0.000076 | -4.93 | A0A5C7I6Y6 | Abhydrolase_3 domain-containing protein | Probable carboxylesterase 7 | At2g03550 | EZV62_011346 |
| 0.00017 | -5.27 | A0A5C7GUE5 | DUF1264 domain-containing protein | Oil body-associated protein 2A | At5g45690 | EZV62_027030 |
| 0.0013 | -5.27 | A0A5C7IXI5 | Cupin type-1 domain-containing protein | 12S seed storage globulin 1 | At5g44120 | EZV62_002569 |
| 0.00016 | -5.44 | A0A5C7H8W5 | Uncharacterized protein | 60S acidic ribosomal protein P2 | At2g27710 | EZV62_022358 |
| 0.000064 | -5.55 | A0A5C7I014 | Peptidase A1 domain-containing protein | Protein ASPARTIC PROTEASE IN GUARD CELL 1 | At3g18490 | EZV62_013671 |
| 0.00006 | -5.75 | A0A5C7IWN4 | MD-2-related lipid-recognition domain-containing protein |  | At5g23820 | EZV62_002087 |
| 0.00027 | -5.83 | A0A5C7HEQ9 | Large ribosomal subunit protein uL30 N-terminal eukaryotes domain-containing protein | 60S ribosomal protein L7-4 | At2g01250 | EZV62_020524 |
| 0.00016 | -5.89 | A0A5C7HYY5 | Uncharacterized protein | ASPARTIC PROTEASE IN GUARD CELL 2 | At3g20015 | EZV62_013674 |
| 0.000095 | -6.71 | A0A5C7H5B5 | Thioredoxin domain-containing protein | Thioredoxin H1 | At3g51030 | EZV62_021330 |
| 0.00016 | -6.72 | A0A5C7GZL1 | Oleosin |  | At4g25140 | EZV62_022744 |
| 0.000044 | -6.76 | A0A5C7H3W8 | Glutathione dehydrogenase (ascorbate) | Glutathione S-transferase DHAR2-like | At1g19570 | EZV62_024131 |
| 0.00016 | -6.78 | A0A5C7HWJ0 | PLAT domain-containing protein | PLAT domain-containing protein 3 | At5g07190 | EZV62_015269 |
| 0.00011 | -7.17 | A0A5C7HD29 | Leucine-rich repeat-containing N-terminal plant-type domain-containing protein |  | At4g15770 | EZV62_019761 |
| 0.000077 | -8.1 | A0A5C7HBV8; A0A5C7HAR1 | Uncharacterized protein | Kunitz trypsin inhibitor 5-like | At1g17860 | EZV62_019228; EZV62_019223 |
| 0.0036 | -8.96 | A0A5C7H9X2 | AAA+ ATPase domain-containing protein | Protein STICHEL | At2g02480 | EZV62_018825 |
| 0.0017 | -9.09 | A0A5C7GTU6 | Non-specific lipid-transfer protein | non-specific lipid-transfer protein D, cotyledon-specific isoform | At1g27950 | EZV62_027508 |
| 0.00016 | -9.76 | A0A5C7GUU2 | Non-specific lipid-transfer protein | non-specific lipid-transfer protein C, cotyledon-specific isoform | At3g43720 | EZV62_027511 |
