# Supplementary material for: Proteomics- and metabolomics-based analysis of the regulation of germination in Norway maple and sycamore embryonic axes
Source: Tree Physiol. 2025 Jan 6;45(2):tpaf003. doi: 10.1093/treephys/tpaf003 (PMC11791354; doi:10.1093/treephys/tpaf003)
Supplement: Table_S5_tpaf003 [file table_s5_tpaf003.docx]

**Table S5.** Functional analysis of significantly regulated proteins in embryonic axes of sycamore seeds at the imbibed stage compared to Norway maple and in terms of molecular function, biological process, cellular compartment and protein class based on Gene Ontology annotation (PANTHER). Child categories are presented for each category. GO terms and numbers (given in brackets) according to PANTHER classification system are followed the number of genes in each category.

|  | **Upregulated in sycamore** | **Downregulated in sycamore** |
| --- | --- | --- |
| **MOLECULAR FUNCTION** | **catalytic activity (GO:0003824) 38**  oxidoreductase activity (GO:0016491) 16  hydrolase activity (GO:0016787) 12  transferase activity (GO:0016740) 7  catalytic activity, acting on a protein (GO:0140096) 5  ligase activity (GO:0016874) 2  lyase activity (GO:0016829) 1  catalytic activity, acting on a nucleic acid (GO:0140640) 1  **binding (GO:0005488) 16**  small molecule binding (GO:0036094) 5  ion binding (GO:0043167) 5  organic cyclic compound binding (GO:0097159) 5  protein binding (GO:0005515) 4  lipid binding (GO:0008289) 3  protein-containing complex binding (GO:0044877) 1  amide binding (GO:0033218) 1  hormone binding (GO:0042562) 1  **transporter activity (GO:0005215) 3**  transmembrane transporter activity (GO:0022857) 3  lipid transporter activity (GO:0005319) 1  **ATP-dependent activity (GO:0140657) 3**  ATP-dependent activity, acting on DNA (GO:0008094) 1  long-chain fatty acid-CoA ligase activity (GO:0004467) 1  **molecular function regulator activity (GO:0098772) 2**  enzyme regulator activity (GO:0030234) 2  molecular function inhibitor activity (GO:0140678) 2  **antioxidant activity (GO:0016209) 1**  No hit for lower level categories.  **molecular transducer activity (GO:0060089) 1**  signaling receptor activity (GO:0038023) 1  **structural molecule activity (GO:0005198) 1**  structural constituent of ribosome (GO:0003735) 1 | **catalytic activity (GO:0003824) 59**  transferase activity (GO:0016740) 25  hydrolase activity (GO:0016787) 16  oxidoreductase activity (GO:0016491) 12  catalytic activity, acting on a protein (GO:0140096) 9  catalytic activity, acting on a nucleic acid (GO:0140640) 7  ligase activity (GO:0016874) 3  lyase activity (GO:0016829) 2  cyclase activity (GO:0009975) 1  **binding (GO:0005488) 27**  organic cyclic compound binding (GO:0097159) 19  small molecule binding (GO:0036094) 10  carbohydrate derivative binding (GO:0097367) 8  protein binding (GO:0005515) 7  lipid binding (GO:0008289) 2  amide binding (GO:0033218) 1  **structural molecule activity (GO:0005198) 7**  structural constituent of cytoskeleton (GO:0005200) 4  structural constituent of ribosome (GO:0003735) 3  **ATP-dependent activity (GO:0140657) 4**  helicase activity (GO:0004386) 2  ATP hydrolysis activity (GO:0016887) 1  ATP-dependent activity, acting on DNA (GO:0008094) 1  **antioxidant activity (GO:0016209) 2**  no hit for lower level categories  **molecular adaptor activity (GO:0060090) 2**  protein-macromolecule adaptor activity (GO:0030674) 2  **molecular function regulator activity (GO:0098772) 2**  enzyme regulator activity (GO:0030234) 2  molecular function activator activity (GO:0140677) 1  **translation regulator activity (GO:0045182) 2**  translation regulator activity, nucleic acid binding (GO:0090079) 2 |
| **BIOLOGICAL PROCESS** | **metabolic process (GO:0008152) 35**  organic substance metabolic process (GO:0071704) 33  cellular metabolic process (GO:0044237) 29  primary metabolic process (GO:0044238) 27  nitrogen compound metabolic process (GO:0006807) 25  biosynthetic process (GO:0009058) 13  catabolic process (GO:0009056) 12  small molecule metabolic process (GO:0044281) 10  secondary metabolic process (GO:0019748) 2  **cellular process (GO:0009987) 33**  cellular metabolic process (GO:0044237) 29  cellular component organization or biogenesis (GO:0071840) 11  cellular response to stimulus (GO:0051716) 4  protein folding (GO:0006457) 3  cellular localization (GO:0051641) 2  cell communication (GO:0007154) 1  process utilizing autophagic mechanism (GO:0061919) 1  signal transduction (GO:0007165) 1  **response to stimulus (GO:0050896) 9**  response to stress (GO:0006950) 7  response to chemical (GO:0042221) 5  cellular response to stimulus (GO:0051716) 4  response to abiotic stimulus (GO:0009628) 4  response to endogenous stimulus (GO:0009719) 1  **localization (GO:0051179) 5**  macromolecule localization (GO:0033036) 5  establishment of localization (GO:0051234) 4  cellular localization (GO:0051641) 2  maintenance of location (GO:0051235) 1  **biological regulation (GO:0065007) 2**  regulation of biological process (GO:0050789)  **detoxification (GO:0098754) 1**  No hit | **cellular process (GO:0009987) 64**  cellular metabolic process (GO:0044237) 46  cellular component organization or biogenesis (GO:0071840) 18  cell cycle (GO:0007049) 7  cellular response to stimulus (GO:0051716) 5  microtubule-based process (GO:0007017) 5  cellular localization (GO:0051641) 4  protein folding (GO:0006457) 3  cell cycle process (GO:0022402) 3  cell division (GO:0051301) 3  cellular developmental process (GO:0048869) 2  signal transduction (GO:0007165) 2  cell communication (GO:0007154) 2  transmembrane transport (GO:0055085) 1  vesicle-mediated transport (GO:0016192) 1  cell growth (GO:0016049) 1  cell wall organization or biogenesis (GO:0071554) 1  **metabolic process (GO:0008152) 53**  organic substance metabolic process (GO:0071704) 51  cellular metabolic process (GO:0044237) 46  primary metabolic process (GO:0044238) 45  nitrogen compound metabolic process (GO:0006807) 35  biosynthetic process (GO:0009058) 29  small molecule metabolic process (GO:0044281) 18  catabolic process (GO:0009056) 11  secondary metabolic process (GO:0019748) 4  glycosylation (GO:0070085) 1  methylation (GO:0032259) 1  pigment metabolic process (GO:0042440) 1  **response to stimulus (GO:0050896) 11**  response to stress (GO:0006950) 9  cellular response to stimulus (GO:0051716) 5  response to abiotic stimulus (GO:0009628) 4  response to chemical (GO:0042221) 4  response to biotic stimulus (GO:0009607) 1  response to external stimulus (GO:0009605) 1  **biological regulation (GO:0065007) 7**  regulation of biological process (GO:0050789) 7  **localization (GO:0051179) 5**  cellular localization (GO:0051641) 4  establishment of localization (GO:0051234) 4  macromolecule localization (GO:0033036) 3  maintenance of location (GO:0051235) 1  organelle localization (GO:0051640) 1  **developmental process (GO:0032502) 2**  anatomical structure development (GO:0048856) 1  anatomical structure morphogenesis (GO:0009653) 1  cellular developmental process (GO:0048869) 2  developmental growth (GO:0048589) 1  developmental process involved in reproduction (GO:0003006) 1  **biological process involved in interspecies interaction between organisms (GO:0044419) 1**  response to other organism (GO:0051707) 1  **growth (GO:0040007) 1**  cell growth (GO:0016049) 1  developmental growth (GO:0048589) 1  **multicellular organismal process (GO:0032501) 1**  no hit  **reproduction (GO:0000003) 1**  reproductive process (GO:0022414) 1  sexual reproduction (GO:0019953) 1  **reproductive process (GO:0022414) 1**  developmental process involved in reproduction (GO:0003006) 1  fertilization (GO:0009566) 1 |
| **CELLULAR COMPONENT** | **cellular anatomical entity (GO:0110165) 30**  intracellular anatomical structure (GO:0005622) 25  cytoplasm (GO:0005737) 19  organelle (GO:0043226) 18  membrane (GO:0016020) 7  cytosol (GO:0005829) 5  cell periphery (GO:0071944) 3  extracellular region (GO:0005576) 3  extracellular space (GO:0005615) 2  membrane-enclosed lumen (GO:0031974) 2  nucleoplasm (GO:0005654) 1  chloroplast stroma (GO:0009570) 1  replication fork (GO:0005657) 1  supramolecular complex (GO:0099080) 1  external encapsulating structure (GO:0030312) 1  **protein-containing complex (GO:0032991) 8**  ribonucleoprotein complex (GO:1990904) 5  nuclear protein-containing complex (GO:0140513) 4  Sm-like protein family complex (GO:0120114) 3  catalytic complex (GO:1902494) 2 | **cellular anatomical entity (GO:0110165) 58**  external encapsulating structure (GO:0030312) 2  extracellular region (GO:0005576) 1  extrinsic component of membrane (GO:0019898) 1  intracellular anatomical structure (GO:0005622) 55  cytoplasm (GO:0005737) 40  organelle (GO:0043226) 35  cytosol (GO:0005829) 9  membrane (GO:0016020) 7  membrane-enclosed lumen (GO:0031974) 5  endomembrane system (GO:0012505) 5  envelope (GO:0031975) 5  supramolecular complex (GO:0099080)5  cell periphery (GO:0071944) 4  cell division site (GO:0032153) 2  cell junction (GO:0030054) 1  nucleoplasm (GO:0005654) 1  organelle subcompartment (GO:0031984) 1  chromosomal region (GO:0098687) 1  side of membrane (GO:0098552) 1  **protein-containing complex (GO:0032991) 20**  catalytic complex (GO:1902494) 9  intracellular protein-containing complex (GO:0140535) 5  membrane protein complex (GO:0098796) 4  ribonucleoprotein complex (GO:1990904) 4  nuclear protein-containing complex (GO:0140513) 3  mitochondrial protein-containing complex (GO:0098798) 1  MCM complex (GO:0042555) 1  cohesin complex (GO:0008278) 1 |
| **PROTEIN CLASS** | **metabolite interconversion enzyme (PC00262) 45**  oxidoreductase (PC00176) 22  hydrolase (PC00121) 10  transferase (PC00220) 8  ligase (PC00142) 2  lyase (PC00144) 2  isomerase (PC00135) 1  **protein modifying enzyme (PC00260) 9**  protease (PC00190) 8  protein phosphatase (PC00195) 1  **RNA metabolism protein (PC00031) 8**  RNA processing factor (PC00147) 4  endoribonuclease (PC00094) 1  general transcription factor (PC00259) 1  **chaperone (PC00072) 4**  No hit for lower level categories.  **DNA metabolism protein (PC00009) 2**  DNA-directed DNA polymerase (PC00018) 1  **translational protein (PC00263) 2**  ribosomal protein (PC00202) 2  **transporter (PC00227) 1**  No hit for lower level categories  **protein-binding activity modulator (PC00095) 1**  protease inhibitor (PC00191) 1  **scaffold/adaptor protein (PC00226) 1**  No hit for lower level categories  **transfer/carrier protein (PC00219) 1**  No hit for lower level categories  **calcium-binding protein (PC00060) 1**  No hit for lower level categories  **membrane traffic protein (PC00150) 1**  No hit for lower level categories | **metabolite interconversion enzyme (PC00262) 48**  transferase (PC00220) 16  oxidoreductase (PC00176) 14  hydrolase (PC00121) 10  lyase (PC00144) 6  ligase (PC00142) 2  **protein modifying enzyme (PC00260) 10**  ubiquitin-protein ligase (PC00234) 3  non-receptor serine/threonine protein kinase (PC00167) 2  protease (PC00190) 2  protein phosphatase (PC00195) 2  **translational protein (PC00263) 9**  aminoacyl-tRNA synthetase (PC00047) 3  ribosomal protein (PC00202) 3  translation factor (PC00223) 2  **RNA metabolism protein (PC00031) 9**  DNA-directed RNA polymerase (PC00019) 2  RNA helicase (PC00032) 2  RNA methyltransferase (PC00033) 1  general transcription factor (PC00259) 1  **cytoskeletal protein (PC00085) 6**  microtubule or microtubule-binding cytoskeletal protein (PC00157) 5  **chaperone (PC00072) 4**  no hit  **gene-specific transcriptional regulator (PC00264) 4**  DNA-binding transcription factor (PC00218) 4  **DNA metabolism protein (PC00009) 3**  endodeoxyribonuclease (PC00093) 1  **protein-binding activity modulator (PC00095) 2**  G-protein (PC00020) 2  **scaffold/adaptor protein (PC00226) 1**  no hit  **membrane traffic protein (PC00150) 1**  no hit  **transmembrane signal receptor (PC00197) 1**  no hit |
