# Supplementary material for: Proteomics- and metabolomics-based analysis of the regulation of germination in Norway maple and sycamore embryonic axes
Source: Tree Physiol. 2025 Jan 6;45(2):tpaf003. doi: 10.1093/treephys/tpaf003 (PMC11791354; doi:10.1093/treephys/tpaf003)
Supplement: Table_S7_tpaf003 [file table_s7_tpaf003.docx]

**Table S7.** The list of proteins identified in our study with calculated changes in abundance assigned as upregulated (log_2_FC>2) and downregulated (log_2_FC<–2) in germinated Norway maple and seeds as compared to imbibed sycamore seeds. Protein name was derived from UniProt database (UniProt Consortium 2021) accessed on February 2024. * Gene abbreviation refers to homological *Arabidopsis thaliana* gene recognized by protein-coding gene classification information knowledgebases. **Amino acid sequences of proteins assigned as uncharacterized, containing a specific domain or identified only to class were extracted from UniProt database and were explicated using PSI-BLAST search method (Bhagwat and Aravind 2007). Green color font of a gene refers to the chloroplastic protein.

| **adj P Val** | **Log_2_FC** | **Majority protein IDs** | **Protein name** | **Explicated protein name *** | **Gene*** | **Gene name** |
| --- | --- | --- | --- | --- | --- | --- |
| 0.0082 | 6.97 | A0A5C7HPG7 | Non-specific lipid-transfer protein | Lipid transfer protein 3 | At5g59320 | EZV62_016799 |
| 0.00052 | 6.65 | A0A5C7HS14 | Endoglucanase | Endo-(1,4)-beta-D-glucanase | At1g68290 | EZV62_014353 |
| 0.000043 | 6.23 | A0A3Q9D3H9 | Cytosolic glyceraldehyde-3-phosphate dehydrogenase |  | At3g04120 |  |
| 0.00013 | 5.83 | A0A5C7GSH9 | Uncharacterized protein | Late embryogenesis abundant protein 2 | At1g02820 | EZV62_026721 |
| 0.000089 | 5.8 | A0A2N9FU94 | Cell division cycle protein 48 |  | At3g09840 | FSB_LOCUS18411 |
| 0.00035 | 5.58 | A0A5C7IN74 | Oleosin |  | At4g25140 | EZV62_005454 |
| 0.00018 | 5.32 | A0A5C7IRX9 | Tr-type G domain-containing protein; Tr-type G domain-containing protein | Elongation factor 2 | At1g56070 | EZV62_006151; EZV62_003912 |
| 0.000053 | 5.27 | A0A5C7IWK4 | 5-methyltetrahydropteroyltriglutamate--homocysteine S-methyltransferase |  | At5g20980 |  |
| 0.00014 | 4.46 | A0A5C7IJQ6 | Glycerol kinase |  | At1g80460 | EZV62_004343 |
| 0.000043 | 4.42 | A0A5C7IGD3; A0A5C7IGQ1; A0A5C7HUB9; A0A5C7IGS6 | SGNH hydrolase-type esterase domain-containing protein | GDSL esterase/lipase 1-like | At5g40990 | EZV62_003136; EZV62_003134; EZV62_014912; EZV62_003139 |
| 0.00044 | 4.4 | A0A5C7H6U8 | SERPIN domain-containing protein | Serpin-ZX | At1g47710 | EZV62_021504 |
| 0.00053 | 4.26 | A0A2N9G734 | GB1/RHD3-type G domain-containing protein | Guanylate-binding protein | At5g44510 | FSB_LOCUS23230 |
| 0.0016 | 4.22 | A0A5C7H3Y3 | CCHC-type domain-containing protein | PLAT domain-containing protein 1 | At4g39730 | EZV62_024151 |
| 0.000071 | 4.16 | A0A5C7IMZ0 | Target of rapamycin complex subunit LST8 |  | At3g18140 |  |
| 0.00013 | 4.01 | A0A2N9GPU4 | Aldehyde dehydrogenase domain-containing protein | Aldehyde dehydrogenase family 2 member B7, mitochondrial | At1g23800 | FSB_LOCUS29321; adh |
| 0.00056 | 3.92 | A0A2N9J5Q8 | Biotin carboxylase |  | At5g35360 | FSB_LOCUS59816 |
| 0.0012 | 3.91 | A0A5C7HN29 | Uncharacterized protein | Glycosyltransferase bc10 | At5g22070 | EZV62_015954 |
| 0.000053 | 3.83 | A0A5C7IWE5 | Poly [ADP-ribose] polymerase; Poly [ADP-ribose] polymerase |  | At2g31320 |  |
| 0.0044 | 3.82 | A0A2N9I9Y5 | 5-methyltetrahydropteroyltriglutamate--homocysteine S-methyltransferase |  | At3g03780 |  |
| 0.000052 | 3.8 | A0A5C7HZZ2 | Uncharacterized protein | Glucose/ribitol dehydrogenase | At3g05260 | EZV62_009817 |
| 0.000043 | 3.69 | A0A5C7GWN1 | GDSL esterase/lipase At1g09390-like |  | At1g09390 | EZV62_024770 |
| 0.014 | 3.69 | A0A5C7GQL9 | AB hydrolase-1 domain-containing protein | Methylesterase 17 | At3g10870 | EZV62_026139; EZV62_026138 |
| 0.00012 | 3.66 | A0A2N9F361 | Transketolase |  | At3g60750 | FSB_LOCUS9126 |
| 0.00029 | 3.64 | A0A5C7HEY0 | Isocitrate lyase |  | At3g21720 | EZV62_020604 |
| 0.00008 | 3.6 | A0A5C7I4X0 | Peptidase A1 domain-containing protein |  | At5g22320 | EZV62_010610 |
| 0.000086 | 3.57 | A0A2N9ITX0 | Eukaryotic translation initiation factor 3 subunit A |  | At4g11420 |  |
| 0.000085 | 3.51 | A0A5C7HWG0 | Starch synthase, chloroplastic/amyloplastic |  | At5g24300 |  |
| 0.000038 | 3.5 | A0A5C7H1Y6 | Tubulin beta chain |  | At2g29550 | EZV62_023036 |
| 0.00011 | 3.48 | A0A5C7H5J2; A0A2N9F3H9 | Ribulose-phosphate 3-epimerase |  | At5g61410 | EZV62_024236; FSB_LOCUS9432 |
| 0.0067 | 3.46 | A0A5C7IYY0; A0A2N9IGU6 | Pyruvate decarboxylase |  | At4g33070 | EZV62_003002; FSB_LOCUS51141 |
| 0.0024 | 3.42 | A0A5C7IJ29; A0A2N9IR40; A0A2N9HI05; A0A2N9EG95 | ATP-dependent RNA helicase | Eukaryotic initiation factor 4A | At1g54270 | EZV62_003451; FSB_LOCUS54717; FSB_LOCUS39156; FSB_LOCUS5869 |
| 0.00046 | 3.38 | A0A5C7ILE7 | Uncharacterized protein | Voltage-dependent L-type calcium channel subunit | At5g16550 |  |
| 0.0007 | 3.38 | A0A5C7H0Y4 | Glutathione transferase | Glutathione S-transferase T1 | At5g41210 | EZV62_023011; FSB_LOCUS58764 |
| 0.00026 | 3.37 | A0A5C7HBC3 | ATP-dependent Clp protease proteolytic subunit |  | At5g45390 |  |
| 0.00067 | 3.31 | A0A5C7H305 | Phosphoethanolamine N-methyltransferase |  | At3g18000 |  |
| 0.00035 | 3.29 | A0A5C7H4Y6 | Enoyl reductase (ER) domain-containing protein | Cinnamyl alcohol dehydrogenase 9 | At4g39330 | EZV62_021180 |
| 0.000043 | 3.18 | A0A5C7IY06 | Glucan endo-1,3-beta-D-glucosidase |  | At3g57260 | EZV62_002652 |
| 0.00018 | 3.18 | A0A5C7HJA0 | Protein-disulfide reductase | Nucleoredoxin 1 | At1g60420 | EZV62_017599; EZV62_017600 |
| 0.00067 | 3.07 | A0A5C7HIK9 | NADP-dependent oxidoreductase domain-containing protein |  | At1g53050 |  |
| 0.000098 | 2.98 | A0A5C7HWJ0 | PLAT domain-containing protein | PLAT domain-containing protein 3 | At5g07190 | EZV62_015269; FSB_LOCUS28422 |
| 0.000075 | 2.97 | A0A5C7I4Y1 | Enoyl reductase (ER) domain-containing protein | Prostaglandin reductase-3 | At2g06050 | EZV62_011324; FSB_LOCUS45461 |
| 0.00012 | 2.94 | A0A5C7I5C4 | Methylcrotonoyl-CoA carboxylase |  | At1g03090 | EZV62_011288 |
| 0.00091 | 2.93 | A0A2N9FRC4 | Peptidase A1 domain-containing protein | Aspartic proteinase A1 | At1g11910 | FSB_LOCUS21149 |
| 0.00081 | 2.89 | A0A5C7I757 | Chitinase |  | At3g12500 | EZV62_011979 |
| 0.00025 | 2.84 | A0A5C7I851; A0A2N9GC06 | Acyl-[acyl-carrier-protein] desaturase |  | At5g16240 | EZV62_006629; FSB_LOCUS24975 |
| 0.047 | 2.84 | A0A5C7HYV7 | Fe2OG dioxygenase domain-containing protein | 1-aminocyclopropane-1-carboxylate oxidase 1 | At2g19590 | EZV62_012909 |
| 0.001 | 2.81 | A0A5C7GUW7 | ENTH domain-containing protein | Putative clathrin assembly protein At1g33340 | At1g33340 | EZV62_024343 |
| 0.000052 | 2.72 | A0A5C7GQQ3 | Fe2OG dioxygenase domain-containing protein | 1-aminocyclopropane-1-carboxylate oxidase | At1g62380 | EZV62_026169 |
| 0.001 | 2.72 | A0A5C7I4S9 | Proline iminopeptidase |  | At2g14260 | EZV62_010992; FSB_LOCUS61256 |
| 0.012 | 2.71 | A0A2N9FGT0 | 14_3_3 domain-containing protein | 14-3-3-like protein B | At5g10450 | FSB_LOCUS14294 |
| 0.003 | 2.67 | A0A5C7HP98 | RRM domain-containing protein | THO complex subunit 4A | At5g59950 | EZV62_016414 |
| 0.015 | 2.66 | A0A5C7HGX0 | ATP citrate synthase |  | At1g10670 | EZV62_017570 |
| 0.0039 | 2.64 | A0A2N9G1L8 | Ubiquitin carboxyl-terminal hydrolase | Ubiquitin carboxyl-terminal hydrolase 12 | At5g06600 | FSB_LOCUS24559 |
| 0.0065 | 2.64 | A0A5C7H403 | Bifunctional inhibitor/plant lipid transfer protein/seed storage helical domain-containing protein | Transcription factor TT8 | At4g09820 | EZV62_023949; FSB_LOCUS45354 |
| 0.00098 | 2.61 | A0A2N9I6S2; A0A5C7IRJ5; A0A5C7IR49; A0A2N9FVX6 | Short-chain dehydrogenase TIC 32, chloroplastic |  | At4g23430 |  |
| 0.0011 | 2.59 | A0A5C7IJH2 | PdxS/SNZ N-terminal domain-containing protein |  | At2g38230 |  |
| 0.0063 | 2.59 | A0A2N9HXU7; A0A2N9HWM6 | Beta-glucosidase |  | At5g36890 | FSB_LOCUS44381; FSB_LOCUS44380 |
| 0.00019 | 2.58 | A0A5C7GU24; A0A2N9EQ15 | 4-hydroxy-tetrahydrodipicolinate reductase |  | At2g44040 |  |
| 0.00017 | 2.57 | A0A5C7HU46 | Inositol-3-phosphate synthase |  | At4g39800 | EZV62_015210; FSB_LOCUS40460 |
| 0.0002 | 2.57 | A0A5C7IBN6; A0A5C7IBL1 | Cytochrome P450 |  | At3g03470 | EZV62_007902; EZV62_007900 |
| 0.01 | 2.57 | A0A5C7HE70 | Large ribosomal subunit protein uL2 C-terminal domain-containing protein |  | At2g44065 |  |
| 0.0014 | 2.56 | A0A5C7HAE2 | Leucine-rich repeat-containing N-terminal plant-type domain-containing protein | LRR receptor-like serine/threonine-protein Kinase | At4g36180 | EZV62_022644 |
| 0.021 | 2.53 | A0A2N9J361; A0A2N9IZY3 | Glutathione peroxidase | Probable glutathione peroxidase 8 | At1g63460 | FSB_LOCUS58771; FSB_LOCUS58768 |
| 0.02 | 2.52 | A0A5C7II35 | GDSL esterase/lipase APG |  | At3g16370 | EZV62_003726 |
| 0.00083 | 2.51 | A0A5C7H5F6 | 40S ribosomal protein S19 |  | At5g47320 | EZV62_024196 |
| 0.00013 | 2.47 | A0A2N9HWR0; A0A2N9H988 | Beta-galactosidase | Beta-galactosidase 8 | At2g28470 | FSB_LOCUS44021; FSB_LOCUS36185 |
| 0.03 | 2.47 | A0A2N9HCL4 | Uncharacterized protein | Elongation factor 1-gamma | At1g57720 | FSB_LOCUS37517 |
| 0.01 | 2.46 | A0A5C7IYR7 | UBC core domain-containing protein | Ubiquitin-conjugating enzyme E2 7 | At5g59300 | EZV62_002603 |
| 0.0012 | 2.44 | A0A5C7IU56 | Eukaryotic translation initiation factor 2A |  | At5g05470 |  |
| 0.00019 | 2.43 | A0A5C7IYT0; A0A5C7IYX9; A0A5C7J0X5; A0A5C7IZI7 | U6 snRNA-associated Sm-like protein LSm8 |  | At1g65700 |  |
| 0.00023 | 2.43 | A0A5C7HJB6 | Glutamine synthetase |  | At5g16570 | EZV62_018212 |
| 0.0006 | 2.42 | A0A5C7I217; A0A2N9HSQ3 | UMP-CMP kinase |  | At3g60180 | EZV62_009446; FSB_LOCUS43204 |
| 0.00026 | 2.4 | A0A5C7I0Y2; A0A5C7I2X2 | Pectate lyase superfamily protein domain-containing protein |  | At1g04680 |  |
| 0.00042 | 2.38 | A0A5C7HIC4 | Purple acid phosphatase |  | At3g17790 | EZV62_017912; EZV62_017911 |
| 0.00081 | 2.37 | A0A5C7IZ87; A0A2N9IPA8 | Malonyl-CoA: ACP transacylase (MAT) domain-containing protein |  | At2g30200 |  |
| 0.0013 | 2.35 | A0A5C7GW72 | FRIGIDA-like protein |  | At5g16320 | EZV62_024848 |
| 0.0013 | 2.33 | A0A5C7HMD8 | 6,7-dimethyl-8-ribityllumazine synthase |  | At2g44050 | EZV62_015857; FSB_LOCUS48726; FSB_LOCUS4537 |
| 0.0018 | 2.3 | A0A5C7IP77 | Pyruvate kinase |  | At5g52920 | EZV62_006006 |
| 0.00049 | 2.26 | A0A5C7HHE0 | Ran guanine nucleotide release factor |  | At1g69680 | EZV62_017796 |
| 0.0036 | 2.26 | A0A2N9G8R3 | NAD(P)-binding domain-containing protein | Trifunctional UDP-glucose 4,6-dehydratase/UDP-4-keto-6-deoxy-D-glucose 3,5-epimerase | At3g14790 | FSB_LOCUS23780; EZV62_007923 |
| 0.0014 | 2.25 | A0A5C7J1B7 | Glycosyltransferase | UDP-glycosyltransferase 87A2 | At2g30140 | EZV62_003035; EZV62_003030 |
| 0.00034 | 2.24 | A0A5C7H9F7 | Peptidase A1 domain-containing protein |  | At3g46910 | EZV62_022351 |
| 0.0052 | 2.22 | A0A5C7IMI0 | EF-hand domain-containing protein | Probable calcium-binding protein CML48 | At2g27480 | EZV62_005233 |
| 0.00013 | 2.19 | A0A2N9HPR7 | Peptidyl-prolyl cis-trans isomerase | Peptidyl-prolyl cis-trans isomerase CYP19-4 | At2g29960 | FSB_LOCUS41501 |
| 0.015 | 2.19 | A0A2N9H3H6 | Haloacid dehalogenase-like hydrolase domain-containing protein 3 |  | At2g38740 |  |
| 0.00035 | 2.16 | A0A5C7I6C0 | NADP-dependent oxidoreductase domain-containing protein | NADP-dependent D-sorbitol-6-phosphate dehydrogenase | At5g51970 | EZV62_011668 |
| 0.0048 | 2.15 | A0A5C7HF07 | Uncharacterized protein | MLP3.9 protein | At3g07610 | EZV62_020840 |
| 0.0082 | 2.15 | A0A5C7I2K1 | Stress-related protein |  | At2g47780 | EZV62_010445; EZV62_010446; FSB_LOCUS218 |
| 0.0025 | 2.13 | A0A5C7HT74 | Enoyl reductase (ER) domain-containing protein | Cinnamyl alcohol dehydrogenase 6 | At4g37970 | EZV62_014631 |
| 0.0026 | 2.12 | A0A5C7I1U3 | Protein kinase domain-containing protein | Mitogen-activated protein kinase 2 | At1g59580 | EZV62_009922 |
| 0.05 | 2.11 | A0A5C7HZH1; A0A5C7I1L5 | Peptidase A1 domain-containing protein | Aspartyl protease family protein At5g10770-like | At5g10770 | EZV62_013673; EZV62_013675 |
| 0.00059 | 2.1 | A0A5C7H721 | Fibronectin type III-like domain-containing protein |  | At3g24440 |  |
| 0.0051 | 2.09 | A0A5C7H2V6 | L-ascorbate oxidase |  | At5g21100 | EZV62_023623; FSB_LOCUS4632 |
| 0.0043 | 2.08 | A0A5C7H415 | Clp R domain-containing protein | Chaperone protein ClpB1 | At1g74310 | EZV62_024191 |
| 0.0061 | 2.06 | A0A5C7IU37 | DUF3700 domain-containing protein |  | At5g43830 | EZV62_000541 |
| 0.0014 | 2.03 | A0A2N9FJ08 | Ribosomal protein | 60S ribosomal protein L10a | At5g22440 | FSB_LOCUS14783 |
| 0.00026 | 2.02 | A0A5C7I877 | DUF538 domain-containing protein |  | At5g01610 | EZV62_006660 |

| **adj P Val** | **Log_2_FC** | **Majority protein IDs** | **Protein name** | **Explicated protein name *** | **Gene*** | **Gene name** |
| --- | --- | --- | --- | --- | --- | --- |
| 0.02 | -2.03 | A0A2N9GXT7 | Mitochondrial ATP synthase 6 kDa subunit |  | At3g46430 | FSB_LOCUS32210 |
| 0.0012 | -2.04 | A0A5C7I5K9 | 4Fe-4S ferredoxin-type domain-containing protein |  | At2g40150 | EZV62_011535; FSB_LOCUS14776; FSB_LOCUS41923 |
| 0.016 | -2.08 | A0A5C7IR33 | Peptide-methionine (R)-S-oxide reductase | Peptide methionine sulfoxide reductase B5 | At4g04830 | EZV62_000422; FSB_LOCUS56879 |
| 0.0048 | -2.09 | A0A5C7IQP3 | Dynein light chain |  | At2g34680 | EZV62_000282 |
| 0.0045 | -2.11 | A0A5C7GQB6 | Glutathione transferase | Putative glutathione S-transferase U25 | At1g17180 | EZV62_026142 |
| 0.00013 | -2.13 | A0A5C7I7S7 | Eukaryotic translation initiation factor 4B3-like |  | At4g38710 | EZV62_011823 |
| 0.0024 | -2.13 | A0A5C7HAG4 | Chlorophyll a-b binding protein, chloroplastic |  | At1g29930 | EZV62_022674;FSB_LOCUS61307 |
| 0.0099 | -2.14 | A0A2N9IHG3 | Ornithine carbamoyltransferase |  | At1g75330 | FSB_LOCUS51422 |
| 0.00013 | -2.15 | A0A5C7GV52 | Uncharacterized protein | Ankyrin-3 | At5g66055 | EZV62_027719 |
| 0.00023 | -2.15 | A0A5C7HHL6 | Bet v I/Major latex protein domain-containing protein | MLP-like protein 423 | At1g24020 | EZV62_017662; FSB_LOCUS56069 |
| 0.0011 | -2.15 | A0A5C7GWV9 | RNA helicase | DEAD-box ATP-dependent RNA helicase 3, chloroplastic | At5g26742 | EZV62_024840 |
| 0.0082 | -2.15 | A0A5C7HYK7 | SHSP domain-containing protein | 17.6 kDa class II heat shock protein | At5g12020 | EZV62_013211 |
| 0.00035 | -2.16 | A0A5C7I9N5 | ER membrane protein complex subunit 2 |  | At3g60600 | EZV62_007169; FSB_LOCUS18633 |
| 0.00079 | -2.16 | A0A5C7INS9 | Uncharacterized protein | Low-temperature-induced 65 kDa protein | At5g52310 | EZV62_005835 |
| 0.039 | -2.16 | A0A5C7IY77 | Uncharacterized protein | Outer envelope pore protein 16, chloroplastic | At2g28900 | EZV62_002648 |
| 0.00011 | -2.17 | A0A5C7INQ6 | Uncharacterized protein | Low-temperature-induced 65 kDa protein-like | At5g52300 | EZV62_005836 |
| 0.00059 | -2.17 | A0A5C7IEW6; A0A5C7IE05; A0A5C7IEY7; A0A5C7IEZ6 | Peptidase A1 domain-containing protein | Aspartyl protease family protein At5g10770-like | At5g10770 | EZV62_008820; EZV62_008818; EZV62_008824; EZV62_008817 |
| 0.00099 | -2.19 | A0A5C7HKC9 | Uncharacterized protein | protein RETICULATA-RELATED 3, chloroplastic | At3g08640 | EZV62_015316; FSB_LOCUS18324 |
| 0.0016 | -2.19 | A0A5C7GXQ0; A0A5C7IV28 | Uncharacterized protein | Mitochondrial dicarboxylate/tricarboxylate transporter DTC | At5g19760 | EZV62_024721; EZV62_001634 |
| 0.0061 | -2.19 | A0A5C7GRH6 | Enoyl reductase (ER) domain-containing protein | Quinone oxidoreductase PIG3-like | At5g61510 | EZV62_026602 |
| 0.00017 | -2.2 | A0A5C7IWC6 | Lipoxygenase | Linoleate 13S-lipoxygenase 2-1, chloroplastic | At1g67560 | EZV62_001763; FSB_LOCUS48027 |
| 0.001 | -2.21 | A0A5C7HQV7 | Aldose 1-epimerase |  | At3g17940 | EZV62_016994 |
| 0.001 | -2.21 | A0A5C7HZ73 | Methylthioribose-1-phosphate isomerase |  | At2g05830 | EZV62_013039;FSB_LOCUS37661;FSB_LOCUS47509 |
| 0.0033 | -2.21 | A0A5C7HYU8; A0A5C7HXI4 | SHSP domain-containing protein | 17.3 kDa class II heat shock protein-like | At4g14830 | EZV62_013212; EZV62_013206 |
| 0.0017 | -2.22 | A0A5C7IAR3 | Peroxidase | Peroxidase 44 | At4g26010 | EZV62_007705; EZV62_007706 |
| 0.0047 | -2.24 | A0A5C7HPI8 | Gibberellin-regulated protein 14 |  | At5g14920 | EZV62_016819 |
| 0.003 | -2.25 | A0A2N9I280 | Uncharacterized protein | Em-like protein GEA1 | At3g51810 | FSB_LOCUS45985 |
| 0.0015 | -2.26 | A0A5C7HKG3 | Uncharacterized protein | Protein fatty acid export 3 | At2g38550 | EZV62_015346 |
| 0.0014 | -2.3 | A0A5C7IMA8 | Pentacotripeptide-repeat region of PRORP domain-containing protein |  | At1g60070 | EZV62_005114 |
| 0.0047 | -2.3 | A0A5C7IW27 | Glycosyltransferase | Flavonol 7-O-rhamnosyltransferase-like | At1g06000 | EZV62_001305 |
| 0.00039 | -2.31 | A0A5C7IY98 | Bacterial surface antigen (D15) domain-containing protein |  | At3g46740 | EZV62_002677 |
| 0.033 | -2.31 | A0A5C7I525 | RRM domain-containing protein | Polyadenylate-binding protein RBP47B | At3g19130 | EZV62_011361 |
| 0.0012 | -2.32 | A0A5C7HLH3 | NAD(P)-bd_dom domain-containing protein | Uncharacterized protein At1g32220, chloroplastic | At1g32220 | EZV62_015670 |
| 0.000094 | -2.33 | A0A5C7HE89 | Prohibitin |  | At4g28510 | EZV62_020344; FSB_LOCUS23228; FSB_LOCUS51610; FSB_LOCUS52698 |
| 0.0069 | -2.33 | A0A5C7HZT5 | Transmembrane 9 superfamily member |  | At1g10950 | EZV62_013641; FSB_LOCUS37750; FSB_LOCUS19604 |
| 0.013 | -2.33 | A0A5C7IPU9 | CCAAT-binding factor domain-containing protein | Nucleolar complex protein 3 homolog | At1g79150 | EZV62_006210 |
| 0.018 | -2.33 | A0A5C7I4M1 | Cinnamyl-alcohol dehydrogenase | Cinnamyl-alcohol dehydrogenase 5 | At4g34230 | EZV62_011211; EZV62_011214; EZV62_011213; FSB_LOCUS33819; FSB_LOCUS33820; EZV62_011212 |
| 0.025 | -2.35 | A0A5C7IRV7 | Cysteine synthase |  | At3g59760 | EZV62_000639 |
| 0.006 | -2.38 | A0A5C7HJS0 | Uncharacterized protein | Mitochondrial outer membrane protein porin of 36 kDa | At5g37610 | EZV62_017769; FSB_LOCUS18 |
| 0.0013 | -2.39 | A0A5C7H1E9 | Enoyl reductase (ER) domain-containing protein | Probable cinnamyl alcohol dehydrogenase 1 | At1g72680 | EZV62_023334 |
| 0.027 | -2.4 | A0A5C7HRU1 | Peptidyl-prolyl cis-trans isomerase |  | At3g54010 | EZV62_014346; FSB_LOCUS34457 |
| 0.00095 | -2.46 | A0A5C7H329 | Malate synthase |  | At5g03860 | EZV62_023835 |
| 0.0041 | -2.47 | A0A5C7HLK1 | Bet_v_1 domain-containing protein | MLP-like protein 43 | At1g70890 | EZV62_018453 |
| 0.0014 | -2.49 | A0A5C7IGC1 | Ubiquitin receptor RAD23 |  | At1g79650 | EZV62_009360 |
| 0.12 | -2.49 | A0A5C7H9D9 | Phosphoglucomutase (alpha-D-glucose-1,6-bisphosphate-dependent |  | At1g70730 | EZV62_022331;FSB_LOCUS6010 |
| 0.0055 | -2.5 | A0A5C7I638 | Knottin scorpion toxin-like domain-containing protein |  | At2g41070 | EZV62_011578 |
| 0.0018 | -2.53 | A0A5C7HAI9 | Glutathione transferase | Glutathione S-transferase Z1 | At2g02390 | EZV62_018791 |
| 0.00032 | -2.56 | A0A5C7HY52 | BURP domain-containing protein | BURP domain protein RD22 | At5g25610 | EZV62_013420 |
| 0.0078 | -2.56 | A0A5C7HVL2 | Uncharacterized protein | 26S proteasome regulatory subunit RPN13 | At2g26590 | EZV62_012524 |
| 0.00026 | -2.58 | A0A5C7GST1 | Uncharacterized protein | DNA-binding protein DDB | At5g58760 | EZV62_027115; FSB_LOCUS2819 |
| 0.0034 | -2.6 | A0A5C7IL80 | (+)-neomenthol dehydrogenase |  | At3g61220 | EZV62_004880 |
| 0.0008 | -2.63 | A0A2N9ENX6 | NADP-dependent oxidoreductase domain-containing protein | NADPH-dependent aldo-keto reductase, chloroplastic-like | At1g17650 | FSB_LOCUS37415 |
| 0.0069 | -2.63 | A0A5C7I1T5 | Translocase of chloroplast |  | At4g02510 | EZV62_010144 |
| 0.00088 | -2.64 | A0A5C7ISU2 | Glutathione transferase | Glutathione S-transferase U7 | At2g29420 | EZV62_000900; EZV62_000901; EZV62_000903 |
| 0.0011 | -2.64 | A0A5C7HZ86 | Ribosomal RNA-processing protein 12-like conserved domain-containing protein |  | At4g27280 | EZV62_013059 |
| 0.012 | -2.64 | A0A5C7HCP2 | Cytochrome c domain-containing protein |  | At5g40810 | EZV62_020066 |
| 0.000043 | -2.65 | A0A5C7IY13; A0A2N9E2H4; A0A5C7HP29; A0A5C7IXX9; A0A5C7HV55; A0A2N9J9W9; A0A2N9EF16 | Histone H4 |  | At1g07660 | EZV62_002597; FSB_LOCUS32211; EZV62_016413; EZV62_002548; EZV62_012344; EZV62_015601; EZV62_023795 |
| 0.00053 | -2.65 | A0A5C7HBC6 | NTF2 domain-containing protein | Nuclear transport factor 2 | At5g60980 | EZV62_019395 |
| 0.021 | -2.67 | A0A5C7H4P3 | Hyaluronan/mRNA-binding protein domain-containing protein |  | At4g20580 | EZV62_023730 |
| 0.0014 | -2.7 | A0A5C7IDL8 | 3-deoxy-manno-octulosonate cytidylyltransferase |  | At1g53000 | EZV62_008274 |
| 0.0025 | -2.7 | A0A5C7IWG6 | Homoserine dehydrogenase |  | At5g21060 | EZV62_002008; FSB_LOCUS16103 |
| 0.00053 | -2.71 | A0A5C7HLE3 | Uncharacterized protein | Outer envelope pore protein 16-3, chloroplastic | At2g42210 | EZV62_015533 |
| 0.0048 | -2.71 | A0A5C7HPR0; A0A5C7I4R5 | Uncoupling protein | Mitochondrial uncoupling protein | At3g54110 | EZV62_016574; EZV62_011244; FSB_LOCUS59566 |
| 0.005 | -2.72 | A0A5C7IRD6 | Peptidase A1 domain-containing protein | Basic 7S globulin | At4g36700 | EZV62_000536 |
| 0.0035 | -2.73 | A0A5C7IFU4 | Electron transfer flavoprotein subunit alpha |  | At1g50940 | EZV62_009139 |
| 0.000053 | -2.74 | A0A5C7HF87 | ADP/ATP translocase |  | At3g08580 | EZV62_020378;EZV62_010993 |
| 0.0011 | -2.74 | A0A5C7H8W5 | Uncharacterized protein | 60S acidic ribosomal protein P2 | At2g27710 | EZV62_022358 |
| 0.0073 | -2.75 | A0A5C7IB73 | DM2 domain-containing protein | Upstream activation factor subunit spp27 | At2g39240 | EZV62_007760 |
| 0.0015 | -2.76 | A0A5C7I394; REV__A0A2N9F6N4; A0A2N9G720 | BEACH-type PH domain-containing protein | Protein SPIRRIG | At1g03060 | EZV62_010715; FSB_LOCUS23051 |
| 0.0022 | -2.8 | A0A5C7HZM6; A0A5C7I2E7 | Malate dehydrogenase |  | At3g47520 | EZV62_009585; EZV62_009586 |
| 0.00047 | -2.81 | A0A5C7HR47 | AB hydrolase-1 domain-containing protein | Salicylic acid-binding protein 2-like | AT2G23550 | EZV62_014090; FSB_LOCUS40054; FSB_LOCUS43230; FSB_LOCUS43228; FSB_LOCUS35416; FSB_LOCUS43229; FSB_LOCUS37768 |
| 0.00068 | -2.82 | A0A5C7IJF4 | Late embryogenesis abundant protein group 3 protein |  | At3g62420 | EZV62_004180 |
| 0.00046 | -2.91 | A0A5C7H2D2; A0A5C7H1U1 | SHSP domain-containing protein | 18.2 kDa class I heat shock protein-like | At5g59720 | EZV62_023469; EZV62_023472 |
| 0.0011 | -2.92 | A0A2N9EIR1 | Non-specific serine/threonine protein kinase | Wall-associated receptor kinase | At1g16120 | FSB_LOCUS2406 |
| 0.0059 | -2.92 | A0A5C7GZ95 | HMA domain-containing protein | Copper transport protein ATX1 | At1g66240 | EZV62_025221 |
| 0.000098 | -2.94 | A0A5C7HK19 | Bet_v_1 domain-containing protein | MLP-like protein 328 | At2g01520 | EZV62_018448 |
| 0.0028 | -2.97 | A0A5C7HY66; A0A2N9H929; A0A2N9EEI2 | Uncharacterized protein | Nucleolin 1-like | At1g48920 | EZV62_013440; FSB_LOCUS36073; FSB_LOCUS5248 |
| 0.048 | -3 | A0A5C7I3L0 | SMP domain-containing protein | Late embryogenesis abundant protein D-34-like | At4g26080 | EZV62_010668 |
| 0.000043 | -3.02 | A0A5C7I164 | FAD_binding_3 domain-containing protein | Monooxygenase 2-like | At1g65860 | EZV62_009991 |
| 0.00026 | -3.02 | A0A5C7IV69 | Enoyl reductase (ER) domain-containing protein | 2-alkenal reductase | At5g16970 | EZV62_001568 |
| 0.00013 | -3.04 | A0A5C7GYS8 | UDP-glucuronate decarboxylase |  | At3g53520 | EZV62_025626 |
| 0.00034 | -3.07 | A0A2N9FS03; A0A5C7HW19; A0A5C7I5B4; A0A2N9G6R6; A0A5C7IJF6 | Histone H2A |  | At1g51060 | FSB_LOCUS17406; EZV62_012686; EZV62_010730; FSB_LOCUS23037; EZV62_004144 |
| 0.02 | -3.07 | A0A2N9FGW8 | Protein CutA, chloroplastic |  | At2g33740 | FSB_LOCUS14295; EZV62_021868 |
| 0.0002 | -3.08 | A0A5C7H8I5 | Variable large protein | D-3-phosphoglycerate dehydrogenase | At4g34200 | EZV62_021635 |
| 0.0041 | -3.09 | A0A5C7IXA6 | Lipoxygenase | LOX2, chloroplastic | At3g45140 | EZV62_001761; EZV62_001758; EZV62_001766; FSB_LOCUS38776 |
| 0.017 | -3.11 | A0A5C7H7S3; A0A5C7H6P4 | Phosphoglycerate mutase-like protein |  | At1g58280 | EZV62_021784; EZV62_021786 |
| 0.000053 | -3.15 | A0A5C7IYC8 | Dihydropyrimidinase |  | At5g12200 | EZV62_002035 |
| 0.00013 | -3.15 | A0A5C7HT41 | Cytochrome P450 | Cytochrome P450 81 | At5g57220 | EZV62_013925; EZV62_025366; EZV62_025365 |
| 0.00052 | -3.15 | A0A5C7IDS2 | Beta-glucosidase | Beta-glucosidase 18 | At1g52400 | EZV62_008354; FSB_LOCUS55323 |
| 0.000052 | -3.19 | A0A5C7IKJ0 | RRM domain-containing protein | THO complex subunit 4D | At5g37720 | EZV62_004852 |
| 0.0007 | -3.27 | A0A5C7I8T1 | GPI-anchored protein |  | At4g26466 | EZV62_006816 |
| 0.04 | -3.27 | A0A5C7HVB2; A0A2N9ELX2; A0A2N9EL89; A0A5C7IV58; A0A2N9HG56; A0A060VD59; A0A5C7H5B4; A0A2N9EDT7; A0A5C7HSE5 | Histone H3 |  | At1g09200 | EZV62_001783; FSB_LOCUS3441; FSB_LOCUS3440; EZV62_001789; FSB_LOCUS32289; his3c2; EZV62_014719; FSB_LOCUS10040; EZV62_014341 |
| 0.0017 | -3.32 | A0A5C7IC74 | EF-hand domain-containing protein | Caleosin | At5g29560 | EZV62_007331 |
| 0.00073 | -3.38 | A0A5C7H1W7 | 4-coumarate--CoA ligase |  | At5g63380 | EZV62_023016; FSB_LOCUS58758; FSB_LOCUS36116 |
| 0.00012 | -3.49 | A0A2N9FT44; A0A5C7IE60 | Histone H2A |  | At4g27230 | FSB_LOCUS18197; EZV62_008613 |
| 0.00035 | -3.55 | A0A5C7HIP6 | Bifunctional inhibitor/plant lipid transfer protein/seed storage helical domain-containing protein |  | At2g41860 | EZV62_017359 |
| 0.00065 | -3.57 | A0A2N9EBC2 | 3-deoxy-manno-octulosonate cytidylyltransferase |  | At1g53000 | FSB_LOCUS4149 |
| 0.0011 | -3.57 | A0A5C7IY26 | Histone H2A |  | At5g27670 | EZV62_002588 |
| 0.00026 | -3.58 | A0A5C7H1K9 | H15 domain-containing protein | Histone H1 | At1g06760 | EZV62_023367 |
| 0.00046 | -3.62 | A0A5C7H2X1 | Actin | Actin-7 | At5g09810 | EZV62_023336 |
| 0.0006 | -3.66 | A0A5C7IHX7; A0A5C7IHD4 | Peptidase M20 dimerisation domain-containing protein |  | At4g20960 | EZV62_003620;EZV62_003635 |
| 0.00013 | -3.67 | A0A5C7IFM5 | SUI1 domain-containing protein |  | At1g54290 | EZV62_009123; FSB_LOCUS1758; FSB_LOCUS5440; FSB_LOCUS55349 |
| 0.000043 | -3.68 | A0A5C7IKG3 | TNFR-Cys domain-containing protein | Kinesin-like protein KIN-12F | At3g20150 | EZV62_004636 |
| 0.0013 | -3.69 | A0A5C7H2H1 | Endoplasmic reticulum transmembrane protein |  | At1g07810 | EZV62_023453 |
| 0.0035 | -3.69 | A0A5C7I2W0 | Co-chaperone protein p23 |  | At4g02450 | EZV62_010439; FSB_LOCUS226 |
| 0.000043 | -3.7 | A0A5C7HH65; A0A2N9HDW5 | Transmembrane protein |  | At1g27990 | EZV62_017338; FSB_LOCUS38254 |
| 0.00011 | -3.74 | A0A2N9IW57 | Uncharacterized protein | Luminal-binding protein | At5g42020 | FSB_LOCUS56163 |
| 0.000085 | -3.75 | A0A5C7H9W3 | DJ-1/PfpI domain-containing protein | protein DJ-1 homolog D | At3g02720 | EZV62_019249 |
| 0.00052 | -3.81 | A0A5C7I1D5 | 60S acidic ribosomal protein P1-like |  | At3g09200 | EZV62_010095 |
| 0.000033 | -3.89 | A0A2N9FFP9 | 14_3_3 domain-containing protein |  | At5g10450 | FSB_LOCUS13880 |
| 0.000056 | -3.92 | A0A5C7ITJ3; A0A5C7ISA1; A0A5C7IRG5; A0A5C7IR15 | AB hydrolase-1 domain-containing protein | Epoxide hydrolase 1 | At1g54990 | EZV62_000275; EZV62_000273; EZV62_000277; EZV62_000274; FSB_LOCUS35396 |
| 0.000052 | -3.97 | A0A2N9I983; A0A2N9FFW1 | 40S ribosomal protein S6 |  | At4g31700 | FSB_LOCUS48293; FSB_LOCUS13877; EZV62_024919 |
| 0.00012 | -3.97 | A0A5C7IBE6 | UDP-glycosyltransferases domain-containing protein | UDP-glycosyltransferase 87A1 | At2g30150 | EZV62_007739 |
| 0.000043 | -4.08 | A0A5C7HB67 | HVA22-like protein |  | At5g50720 | EZV62_019659 |
| 0.00012 | -4.09 | A0A5C7H3W8 | Glutathione dehydrogenase (ascorbate) | Glutathione S-transferase DHAR2-like | At1g19570 | EZV62_024131 |
| 0.000035 | -4.1 | A0A5C7IV03 | DUF1264 domain-containing protein | Oil body-associated protein 1A | At1g05510 | EZV62_001498 |
| 0.00023 | -4.14 | A0A2N9FNR3; A0A5C7IVM4 | Uncharacterized protein | Pentatricopeptide repeat-containing protein At5g27110 | At5g27110 | FSB_LOCUS16246; EZV62_001930 |
| 0.00023 | -4.29 | A0A5C7HEQ9 | Large ribosomal subunit protein uL30 N-terminal eukaryotes domain-containing protein | 60S ribosomal protein L7-4 | At2g01250 | EZV62_020524 |
| 0.000053 | -4.31 | A0A5C7H0R5 | SHSP domain-containing protein | 18.1 kDa class I heat shock protein | At5g59720 | EZV62_025751; FSB_LOCUS21634 |
| 0.00035 | -4.44 | A0A5C7I6Y6 | Abhydrolase_3 domain-containing protein | Probable carboxylesterase 7 | At2g03550 | EZV62_011346 |
| 0.00013 | -4.47 | A0A5C7GUE5 | DUF1264 domain-containing protein | Oil body-associated protein 2A | At5g45690 | EZV62_027030 |
| 0.00012 | -4.71 | A0A5C7ITB5 | AWPM-19-like family protein | AWPM-19-like | At5g46530 | EZV62_001024 |
| 0.000015 | -5.09 | A0A5C7IPQ6 | Reticulon-like protein |  | At4g23630 | EZV62_005974 |
| 0.000052 | -5.13 | A0A5C7HYY5 | Uncharacterized protein | ASPARTIC PROTEASE IN GUARD CELL 2 | At3g20015 | EZV62_013674 |
| 0.000038 | -5.31 | A0A5C7HFB2 | Ubiquitin-like domain-containing protein | Small ubiquitin-related modifier 1-like | At4g26840 | EZV62_020674 |
| 0.000048 | -5.34 | A0A5C7HLQ9 | Uncharacterized protein | Late embryogenesis abundant protein 46 | At5g06760 | EZV62_015786 |
| 0.000053 | -5.52 | A0A5C7IVU9; A0A5C7ISU3; A0A5C7IU02 | RRM domain-containing protein | Glycine-rich RNA-binding protein 2, mitochondrial | At4g13850 | EZV62_001221; EZV62_000483; EZV62_000481 |
| 0.0048 | -5.62 | A0A5C7GZL1 | Oleosin |  | At4g25140 | EZV62_022744 |
| 0.000035 | -5.66 | A0A5C7GWR1 | Peptidase A1 domain-containing protein | Aspartic proteinase | At1g11910 | EZV62_024812; FSB_LOCUS2255 |
| 0.000038 | -5.74 | A0A5C7HBV8; A0A5C7HAR1 | Uncharacterized protein | Kunitz trypsin inhibitor 5-like | At1g17860 | EZV62_019228; EZV62_019223 |
| 0.0002 | -5.74 | A0A5C7IMW0 | S5 DRBM domain-containing protein |  | At1g64880 | EZV62_005373 |
| 0.000015 | -5.76 | A0A5C7IAE2 | Glycosyltransferase | UDP-glycosyltransferase 87 | At2g30150 | EZV62_007738; FSB_LOCUS14547; FSB_LOCUS14549 |
| 0.00031 | -5.79 | A0A2N9IJK2 | Thioredoxin-dependent peroxiredoxin | 2-Cys peroxiredoxin BAS1, chloroplastic | At3g11630 | FSB_LOCUS52924 |
| 0.00012 | -5.9 | A0A2N9F5C5 | NAC-A/B domain-containing protein | Nascent polypeptide-associated complex subunit alpha-like protein | At5g13850 | FSB_LOCUS10214 |
| 0.000027 | -6.36 | A0A2N9ECJ3 | SHSP domain-containing protein | 17.8 kDa class I heat shock protein-like | At1g07400 | FSB_LOCUS394 |
| 0.00081 | -6.89 | A0A5C7GUU2 | Non-specific lipid-transfer protein | Non-specific lipid-transfer protein C, cotyledon-specific isoform | At3g43720 | EZV62_027511 |
| 0.000035 | -6.99 | A0A2N9I2F3 | Peptidyl-prolyl cis-trans isomerase |  | At3g54010 | FSB_LOCUS46724 |
| 0.0011 | -8.21 | A0A5C7H9X2 | AAA+ ATPase domain-containing protein | Protein STICHEL | At2g02480 | EZV62_018825 |
| 0.001 | -8.75 | A0A5C7GTU6 | Non-specific lipid-transfer protein | Non-specific lipid-transfer protein D, cotyledon-specific isoform | At1g27950 | EZV62_027508 |
