# Supplementary material for: Proteomics- and metabolomics-based analysis of the regulation of germination in Norway maple and sycamore embryonic axes
Source: Tree Physiol. 2025 Jan 6;45(2):tpaf003. doi: 10.1093/treephys/tpaf003 (PMC11791354; doi:10.1093/treephys/tpaf003)
Supplement: Table_S8_tpaf003 [file table_s8_tpaf003.docx]

**Table S8.** Functional analysis of significantly regulated proteins in embryonic axes with protruded radicles of sycamore seeds at the germinated stage compared to Norway maple and in terms of molecular function, biological process, cellular compartment and protein class based on Gene Ontology annotation (PANTHER). Child categories are presented for each category. GO terms and numbers (given in brackets) according to PANTHER classification system are followed the number of genes in each category.

|  | **Upregulated in sycamore** | **Downregulated in sycamore** |
| --- | --- | --- |
| **MOLECULAR FUNCTION** | **catalytic activity (GO:0003824) 40**  oxidoreductase activity (GO:0016491) 13  transferase activity (GO:0016740) 12  hydrolase activity (GO:0016787) 8  catalytic activity, acting on a protein (GO:0140096) 5  isomerase activity (GO:0016853) 5  catalytic activity, acting on a nucleic acid (GO:0140640) 1  ligase activity (GO:0016874) 1  lyase activity (GO:0016829) 1  **binding (GO:0005488) 33**  organic cyclic compound binding (GO:0097159) 18  protein binding (GO:0005515) 10  unfolded protein binding (GO:0051082) 4  protein-containing complex binding (GO:0044877) 8  small molecule binding (GO:0036094) 5  **transporter activity (GO:0005215) 5**  transmembrane transporter activity (GO:0022857) 5  **ATP-dependent activity (GO:0140657) 3**  ATP hydrolysis activity (GO:0016887) 1  ATP-dependent activity, acting on DNA (GO:0008094) 1  ATPase-coupled transmembrane transporter activity (GO:0042626) 1  microtubule motor activity (GO:0003777) 1  **antioxidant activity (GO:0016209) 3**  no hit  **structural molecule activity (GO:0005198) 3**  structural constituent of ribosome (GO:0003735) 3  **cytoskeletal motor activity (GO:0003774) 1**  microtubule motor activity (GO:0003777) 1  **molecular adaptor activity (GO:0060090) 1**  protein-macromolecule adaptor activity (GO:0030674) 1  **molecular function regulator activity (GO:0098772) 1**  enzyme regulator activity (GO:0030234) 1  molecular function activator activity (GO:0140677) 1  **transcription regulator activity (GO:0140110) 1**  DNA-binding transcription factor activity (GO:0003700) 1  **translation regulator activity (GO:0045182) 1**  translation regulator activity, nucleic acid binding (GO:0090079) 1 | **catalytic activity (GO:0003824) 43**  transferase activity (GO:0016740) 13  hydrolase activity (GO:0016787) 12  oxidoreductase activity (GO:0016491) 10  catalytic activity, acting on a protein (GO:0140096) 9  lyase activity (GO:0016829) 4  isomerase activity (GO:0016853) 3  demethylase activity (GO:0032451) 1  ligase activity (GO:0016874) 1  **binding (GO:0005488) 16**  organic cyclic compound binding (GO:0097159) 10  protein binding (GO:0005515) 4  protein-containing complex binding (GO:0044877) 3  small molecule binding (GO:0036094) 3  ion binding (GO:0043167) 3  amide binding (GO:0033218) 1  carbohydrate derivative binding (GO:0097367) 1  lipid binding (GO:0008289) 1  **translation regulator activity (GO:0045182) 4**  translation regulator activity, nucleic acid binding (GO:0090079) 4  **ATP-dependent activity (GO:0140657) 3**  ATP hydrolysis activity (GO:0016887) 2  **structural molecule activity (GO:0005198) 3**  structural constituent of ribosome (GO:0003735) 2  structural constituent of cytoskeleton (GO:0005200) 1  **molecular adaptor activity (GO:0060090) 1**  protein-macromolecule adaptor activity (GO:0030674) 1  **molecular function regulator activity (GO:0098772) 1**  enzyme regulator activity (GO:0030234) 1  **antioxidant activity (GO:0016209) 1**  no hit  **transcription regulator activity (GO:0140110) 1**  transcription coregulator activity (GO:0003712) 1 |
| **BIOLOGICAL PROCESS** | **cellular process (GO:0009987) 50**  cellular metabolic process (GO:0044237) 34  cellular component organization or biogenesis (GO:0071840) 13  cellular localization (GO:0051641) 7  cellular response to stimulus (GO:0051716) 7  microtubule-based process (GO:0007017) 1  protein folding (GO:0006457) 5  cell communication (GO:0007154) 4  signal transduction (GO:0007165) 4  transmembrane transport (GO:0055085) 3  vesicle-mediated transport (GO:0016192) 1  **metabolic process (GO:0008152) 43**  organic substance metabolic process (GO:0071704) 41  cellular metabolic process (GO:0044237) 34  primary metabolic process (GO:0044238) 34  nitrogen compound metabolic process (GO:0006807) 29  biosynthetic process (GO:0009058) 17  small molecule metabolic process (GO:0044281) 11  catabolic process (GO:0009056) 7  secondary metabolic process (GO:0019748) 2  pigment metabolic process (GO:0042440) 1  **response to stimulus (GO:0050896) 15**  response to stress (GO:0006950) 10  cellular response to stimulus (GO:0051716) 7  response to abiotic stimulus (GO:0009628) 6  response to chemical (GO:0042221) 5  response to endogenous stimulus (GO:0009719) 1  **localization (GO:0051179) 11**  cellular localization (GO:0051641) 7  establishment of localization (GO:0051234) 7  macromolecule localization (GO:0033036) 7  maintenance of location (GO:0051235) 1  organelle localization (GO:0051640) 1  **biological regulation (GO:0065007) 10**  regulation of biological process (GO:0050789) 10  **homeostatic process (GO:0042592) 2**  cellular homeostasis (GO:0019725) 2  chemical homeostasis (GO:0048878) 1 | **cellular process (GO:0009987) 37**  cellular metabolic process (GO:0044237) 29  cellular response to stimulus (GO:0051716) 6  cellular component organization or biogenesis (GO:0071840) 5  cellular localization (GO:0051641) 4  signal transduction (GO:0007165) 3  cell communication (GO:0007154) 3  cell cycle (GO:0007049) 2  microtubule-based process (GO:0007017) 2  transmembrane transport (GO:0055085) 1  vesicle-mediated transport (GO:0016192) 1  protein folding (GO:0006457) 1  cell cycle process (GO:0022402) 1  process utilizing autophagic mechanism (GO:0061919) 1  export from cell (GO:0140352) 1  **metabolic process (GO:0008152) 34**  organic substance metabolic process (GO:0071704) 34  cellular metabolic process (GO:0044237) 29  primary metabolic process (GO:0044238) 28  nitrogen compound metabolic process (GO:0006807) 25  biosynthetic process (GO:0009058) 20  small molecule metabolic process (GO:0044281) 17  catabolic process (GO:0009056) 6  secondary metabolic process (GO:0019748) 2  pigment metabolic process (GO:0042440) 1  **response to stimulus (GO:0050896) 8**  cellular response to stimulus (GO:0051716) 6  response to stress (GO:0006950) 5  response to abiotic stimulus (GO:0009628) 1  response to biotic stimulus (GO:0009607) 1  response to chemical (GO:0042221) 1  response to external stimulus (GO:0009605) 1  **localization (GO:0051179) 7**  establishment of localization (GO:0051234) 5  macromolecule localization (GO:0033036) 5  cellular localization (GO:0051641) 4  maintenance of location (GO:0051235) 1  **biological regulation (GO:0065007) 7**  regulation of biological process (GO:0050789) 6  regulation of biological quality (GO:0065008) 2  **biological process involved in interspecies interaction between organisms (GO:0044419) 1**  response to other organism (GO:0051707) 1 |
| **CELLULAR COMPONENT** | **cellular anatomical entity (GO:0110165) 60**  external encapsulating structure (GO:0030312) 1  intracellular anatomical structure (GO:0005622) 53  cytoplasm (GO:0005737) 35  organelle (GO:0043226) 35  membrane (GO:0016020) 13  cytosol (GO:0005829) 12  membrane-enclosed lumen (GO:0031974) 5  cell periphery (GO:0071944) 4  endomembrane system (GO:0012505) 4  envelope (GO:0031975) 3  nucleochloroplast stroma (GO:0009570) 2  nucleoplasm (GO:0005654) 1  organelle subcompartment (GO:0031984) 1  cell junction (GO:0030054) 1  replication fork (GO:0005657) 1  supramolecular complex (GO:0099080) 1  **protein-containing complex (GO:0032991) 13**  ribonucleoprotein complex (GO:1990904) 4  protein-DNA complex (GO:0032993) 3  membrane protein complex (GO:0098796) 2  mitochondrial protein-containing complex (GO:0098798) 2  catalytic complex (GO:1902494) 1  endoplasmic reticulum protein-containing complex (GO:0140534) 1  intracellular protein-containing complex (GO:0140535) 1  microtubule associated complex (GO:0005875) 1  proteasome accessory complex (GO:0022624) 1  proteasome regulatory particle (GO:0005838) 1  proteasome regulatory particle, lid subcomplex (GO:0008541) 1 | **cellular anatomical entity (GO:0110165) 38**  intracellular anatomical structure (GO:0005622) 34  cytoplasm (GO:0005737) 27  organelle (GO:0043226) 21  cytosol (GO:0005829) 10  membrane (GO:0016020) 5  membrane-enclosed lumen (GO:0031974) 4  cell periphery (GO:0071944) 2  chloroplast stroma (GO:0009570) 2  endomembrane system (GO:0012505) 2  supramolecular complex (GO:0099080) 2  cell junction (GO:0030054) 1  external encapsulating structure (GO:0030312) 1  extracellular region (GO:0005576) 1  nucleoplasm (GO:0005654) 1  organelle subcompartment (GO:0031984) 1  **protein-containing complex (GO:0032991) 11**  ribonucleoprotein complex (GO:1990904) 6  catalytic complex (GO:1902494) 3  mitochondrial protein-containing complex (GO:0098798) 2  nuclear protein-containing complex (GO:0140513) 2  Sm-like protein family complex (GO:0120114) 1  endoplasmic reticulum protein-containing complex (GO:0140534) 1  eukaryotic translation initiation factor 3 complex (GO:0005852) 1  intracellular protein-containing complex (GO:0140535) 1  membrane protein complex (GO:0098796) 1  protein-DNA complex (GO:0032993) 1 |
| **PROTEIN CLASS** | **metabolite interconversion enzyme (PC00262) 39**  oxidoreductase (PC00176) 17  transferase (PC00220) 8  hydrolase (PC00121) 7  isomerase (PC00135) 4  lyase (PC00144) 2  ligase (PC00142) 1  **translational protein (PC00263) 8**  ribosomal protein (PC00202) 5  translation factor (PC00223) 3  **transporter (PC00227) 7**  secondary carrier transporter (PC00258) 3  primary active transporter (PC00068) 2  **protein modifying enzyme (PC00260) 6**  protease (PC00190) 4  protein phosphatase (PC00195) 1  non-receptor serine/threonine protein kinase (PC00167) 1  **chromatin/chromatin-binding, or -regulatory protein (PC00077) 6**  no hit  **chaperone (PC00072) 6**  Hsp70 family chaperone (PC00027) 1  **RNA metabolism protein (PC00031) 4**  RNA helicase (PC00032) 1  general transcription factor (PC00259) 1  **membrane traffic protein (PC00150) 4**  membrane trafficking regulatory protein (PC00151) 1  **gene-specific transcriptional regulator (PC00264) 3**  DNA-binding transcription factor (PC00218) 3  **DNA metabolism protein (PC00009) 3**  damaged DNA-binding protein (PC00086) 2  DNA-directed DNA polymerase (PC00018) 1  **cytoskeletal protein (PC00085) 2**  actin or actin-binding cytoskeletal protein (PC00041) 1  microtubule or microtubule-binding cytoskeletal protein (PC00157) 1  **protein-binding activity modulator (PC00095) 2**  G-protein (PC00020) 1  protease inhibitor (PC00191) 1  **scaffold/adaptor protein (PC00226) 1**  no hit  **transfer/carrier protein (PC00219) 1**  no hit  **transmembrane signal receptor (PC00197) 1**  no hit | **metabolite interconversion enzyme (PC00262) 41**  oxidoreductase (PC00176) 15  transferase (PC00220) 11  hydrolase (PC00121) 8  lyase (PC00144) 4  ligase (PC00142) 2  isomerase (PC00135) 1  **protein modifying enzyme (PC00260) 9**  protease (PC00190) 6  non-receptor serine/threonine protein kinase (PC00167) 2  ubiquitin-protein ligase (PC00234) 1  **translational protein (PC00263) 6**  ribosomal protein (PC00202) 3  translation factor (PC00223) 3  **RNA metabolism protein (PC00031) 3**  RNA helicase (PC00032) 1  RNA processing factor (PC00147) 1  **transporter (PC00227) 2**  ion channel (PC00133) 1  primary active transporter (PC00068) 1  **transmembrane signal receptor (PC00197) 1**  no hit  **protein-binding activity modulator (PC00095) 1**  protease inhibitor (PC00191) 1  **scaffold/adaptor protein (PC00226) 1**  no hit  **DNA metabolism protein (PC00009) 1**  no hit  **chaperone (PC00072) 1**  no hit  **chromatin/chromatin-binding, or -regulatory protein (PC00077) 1**  histone modifying enzyme (PC00261) 1  **cytoskeletal protein (PC00085) 1**  microtubule or microtubule-binding cytoskeletal protein (PC00157) 1  **gene-specific transcriptional regulator (PC00264) 1**  DNA-binding transcription factor (PC00218) 1  **membrane traffic protein (PC00150) 1**  vesicle coat protein (PC00235) 1 |
