# Supplementary material for: Proteomics- and metabolomics-based analysis of the regulation of germination in Norway maple and sycamore embryonic axes
Source: Tree Physiol. 2025 Jan 6;45(2):tpaf003. doi: 10.1093/treephys/tpaf003 (PMC11791354; doi:10.1093/treephys/tpaf003)
Supplement: Table_S10_tpaf003 [file table_s10_tpaf003.docx]

**Table S10.** The list of identified proteins containing methionine sulfoxide (MetO) in our study with calculated changes in abundance assigned as upregulated (log_2_FC>2) and downregulated (log_2_FC<–2) in imbibed Norway maple seeds as compared to sycamore seeds. Protein name was derived from UniProt database (UniProt Consortium 2021) accessed on February 2024. * Gene abbreviation refers to homological *Arabidopsis thaliana* gene recognized by protein-coding gene classification information knowledgebases. **Amino acid sequences of proteins assigned as uncharacterized, containing a specific domain or identified only to class were extracted from UniProt database and were explicated using PSI-BLAST search method (Bhagwat and Aravind 2007). Green color font of a gene refers to the chloroplastic protein.

| **adj P Val** | **Log_2_FC** | **Proteins** | **Protein name** | **Name*** | **Gene*** | **Positions within proteins** | **Localization prob** | **PEP** | **Unique_identifier** | **Score for localization** | **Sequence window** | **Nr of Oxidation M** |
| --- | --- | --- | --- | --- | --- | --- | --- | --- | --- | --- | --- | --- |
| 0.0000045 | 7.74 | A0A5C7I9G4 | Rieske domain-containing protein | protochlorophyllide-dependent translocon component 52, chloroplastic-like | At4g25650 | 245 | 1 | 0.0338587 | UID637 | 55.084 | AATKQSAMSSTARTTMVSMAVICFAASRCIE | 2 |
| 0.0000013 | 7.63 | A0A5C7I9G4 | Rieske domain-containing protein | protochlorophyllide-dependent translocon component 52, chloroplastic-like | At4g25650 | 248 | 1 | 0.0338587 | UID638 | 55.084 | KQSAMSSTARTTMVSMAVICFAASRCIEVSQ | 2 |
| 0.0000056 | 6.59 | A0A5C7HLQ1 | ATP synthase subunit beta (mitochondrial) |  | At5g08690 | 117 | 1 | 2.46106E-05 | UID162 | 87.49 | RFDDGLPPILTALEVMDHSIRVVLEVASHLG | 1 |
| 0.000012 | 5.83 | A0A5C7GSL4 | BED-type domain-containing protein | Zinc finger BED domain-containing protein RICESLEEPER | At3g56820 | 144 | 1 | 0.00192996 | UID25 | 74.789 | YDLARMIILHGYPLTMVDHVGFRVFVKNLQP | 1 |
| 0.0000056 | 5.47 | A0A5C7ITA3 | SMP domain-containing protein | Late embryogenesis abundant protein D-34 | At4g26080 | 57 | 0.997223 | 5.13903E-42 | UID339 | 204.13 | SGDLANKPVAPRDAAMMQAAETVAFGDTQRG | 1 |
| 0.0000065 | 5.32 | A0A5C7HDY5 | Phospholipase A1 |  | At4g18550 | 534 | 1 | 3.15359E-23 | UID136 | 89.773 | TIQQTLGGVTEQGNQMAKHQGGDNRGGQQSL | 1 |
| 0.000012 | 5.28 | A0A5C7H7N6 | Uncharacterized protein | No Arabidopsis homologue |  | 1 | 1 | 0.0229813 | UID94 | 42.599 | _______________MGEEVAKSVKTETKKG |  |
| 0.000094 | 4.86 | A0A5C7HNS1 | UspA domain-containing protein | Universal stress protein PHOS32 | At5g54430 | 174 | 1 | 2.82236E-13 | UID176 | 126.02 | LVMGSRGLSTIQRILMGSVSNHLMTHAPCPV | 1 |
| 0.00095 | 4.69 | A0A5C7H8B7 | Peroxidase | Peroxidase 12 | At1g71695 | 317 | 1 | 1.78827E-14 | UID100 | 142.07 | LFFDQFALSMIKMGQMSVLTGTLGEVRANCS | 1;2 |
| 0.000069 | 4.62 | A0A5C7I5P7 | 40S ribosomal protein S25 |  | At2g21580 | 47 | 1 | 8.72587E-29 | UID250 | 144.88 | PFKWSKGKQKEKVNNMVLFDQGTYDKLLTEA | 1 |
| 0.0000082 | 4.57 | A0A5C7HNS1 | UspA domain-containing protein | Universal stress protein PHOS32 | At5g54430 | 117 | 1 | 2.19687E-06 | UID177 | 68.809 | FREPEIMQKYQVKPDMEVLDLLDTVSRQKEI | 1 |
| 0.0000056 | 4.44 | A0A5C7HDM3 | 26S proteasome non-ATPase regulatory subunit 2 homolog |  | At1g64520 | 416 | 1 | 0.0145857 | UID131 | 72.891 | FVNAFVNAGFGQDKLMTVPADASSGGASGNW | 1 |
| 0.0000082 | 4.21 | A0A5C7IWK4 | 5-methyltetrahydropteroyltriglutamate--homocysteine S-methyltransferase |  | At5g17920 | 488 | 1 | 0.00019507 | UID359 | 65.111 | LPILPTTTIGSFPQTMDLRRVRREYKAKKIS | 1 |
| 0.000013 | 4.18 | A0A5C7HXI3 | SHSP domain-containing protein | 17.7 kDa class II heat shock protein | At5g12030 | 6 | 0.973052 | 1.76653E-07 | UID220 | 77.566 | __________MDFRIMGMDSPLFSTLQHMID | 1 |
| 0.0000082 | 4.15 | A0A5C7IDK6 | Uncharacterized protein | GLABROUS1 enhancer-binding protein-like | At4g00270 | 1 | 1 | 0.0301174 | UID271 | 49.358 | _______________MATAIGEKQLQTHDDD | 1 |
| 0.0000091 | 4.15 | A0A5C7IB53 | Glutathione transferase | Glutathione S-transferase | At2g02390 | 47 | 1 | 1.48776E-13 | UID264 | 92.098 | YRDEDLSNKSPLLLQMNPVHKKIPVLVHNGK | 1 |
| 0.000012 | 4.01 | A0A5C7IXN9 | Osmotin-like protein |  | At4g11650 | 241 | 1 | 9.2152E-38 | UID369 | 56.29 | ACPATFTYAHDSPTLMHECSSPRELKVIFCH | 1 |
| 0.000034 | 3.97 | A0A5C7IWA8 | Uncharacterized protein | Enoyl-[acyl-carrier-protein] reductase [NADH], chloroplastic | At2g05990 | 219 | 1 | 2.64673E-12 | UID354 | 68.657 | SIDILVHSLANGPEVMKPLLETSRKGYLAAL | 1 |
| 0.000012 | 3.89 | A0A5C7GZ05;A0A5C7IXL3 | Uncharacterized protein | Heat shock cognate 70 kDa protein | At5g02490 | 64;64 | 1 | 3.38286E-21 | UID39 | 52.112 | DSERLIGDAAKNQVAMNPTNTVFDAKRLIGR;DTERLIGDAAKNQVAMNPTNTVFDAKRLIGR | 1 |
| 0.0025 | 3.88 | A0A5C7H8H1;A0A5C7HVB3 | Annexin |  | At1g35720 | 165; 205 | 1 | 0.0104402 | UID105 | 82.417 | LPLVSSYRYEGDEVNMTLAKSEAKILHEKIS;VPLVCSFRYEGDEVNMTLAKKEAKIIREAIS | 1 |
| 0.000074 | 3.65 | A0A5C7H8B7 | Peroxidase | Peroxidase 12 | At1g71695 | 314 | 1 | 2.87292E-11 | UID477 | 142.07 | DEKLFFDQFALSMIKMGQMSVLTGTLGEVRA | 1;2 |
| 0.000028 | 3.62 | A0A5C7H8B7 | Peroxidase | Peroxidase 12 | At1g71695 | 314 | 1 | 2.87292E-11 | UID99 | 142.07 | DEKLFFDQFALSMIKMGQMSVLTGTLGEVRA | 1;2 |
| 0.000079 | 3.62 | A0A5C7I488 | Sucrose synthase |  | At5g20830 | 530 | 1 | 3.53444E-10 | UID244 | 124.45 | DVFDTKFNIVSPGADMDIYFPYSEKQKRLTA | 1 |
| 0.000013 | 3.57 | A0A5C7H3K9 | Glyceraldehyde-3-phosphate dehydrogenase |  | At3g04120 | 128 | 1 | 3.95454E-36 | UID76 | 97.916 | GAKKVVISAPSKDAPMFVVGVNEHEYKPELD | 1 |
| 0.000017 | 3.54 | A0A5C7HAS1 | Methylmalonate-semialdehyde dehydrogenase (CoA acylating) |  | At2g14170 | 102 | 1 | 1.13028E-21 | UID120 | 154.28 | FKFQELIRRDMDKLAMNITTEQGKTLKDAHG | 1 |
| 0.000047 | 3.46 | A0A5C7GZY2 | Aminobutyraldehyde dehydrogenase |  | At3g48170 | 140 | 1 | 0.0238449 | UID55 | 89.171 | AEALDAKQKAPVSLPMDNFKCYILKEPIGVV | 1 |
| 0.000059 | 3.39 | A0A5C7HZX4 | Transketolase |  | At3g60750 | 466 | 1 | 6.35171E-11 | UID228 | 73.91 | LPGFLGGSADLASSNMTLLKMFGDFQKDTPE | 1 |
| 0.00034 | 3.37 | A0A5C7I4G8 | Ribosomal protein L19 |  | At1g02780 | 34 | 1 | 6.47721E-07 | UID247 | 80.522 | RGKVWLDPNEGNEISMANSRQNIRKLVKDGF | 1 |
| 0.000022 | 3.28 | A0A5C7GXS3; A0A5C7HMY6 | 40S ribosomal protein S17 |  | At1g79850 | 58; 58 | 1 | 0.0054843 | UID34 | 112.84 | SKRLRNKIAGFSTHLMKRIQKGPVRGISLKL | 1 |
| 0.000069 | 3.27 | A0A5C7H8B7 | Peroxidase | Peroxidase 12 | At1g71695 | 317 | 1 | 1.78827E-14 | UID478 | 142.07 | LFFDQFALSMIKMGQMSVLTGTLGEVRANCS | 1;2 |
| 0.000034 | 3.21 | A0A5C7HTV7 | Enoyl reductase (ER) domain-containing protein | Alcohol dehydrogenase | At1g77120 | 340 | 1 | 1.10391E-07 | UID201 | 81.534 | IREDAIEKIREITGGMGVDIAVEALGKPQTF | 1 |
| 0.0049 | 3.21 | A0A5C7IQ04 | Peroxiredoxin | 1-Cys peroxiredoxin | At1g48130 | 211 | 1 | 3.54559E-09 | UID326 | 79.693 | EMFPKGYQTVDLPSKMDYLRFTNVV______ | 1 |
| 0.000028 | 3.18 | A0A5C7HSN6 | Aconitate hydratase |  | At4g35830 | 626 | 1 | 9.10862E-13 | UID198 | 44.615 | DMFKATYEAITKGNPMWNQLSVPSGTLYAWD | 1 |
| 0.0001 | 3.18 | A0A5C7HRJ0 | Uncharacterized protein | Late embryogenesis abundant protein LEA_5 subgroup | At3g51810 | 199 | 1 | 5.9858E-28 | UID193 | 94.128 | GQTRKDQLSHEGYQEMGRKGGLSTMEKSGAQ | 1 |
| 0.00013 | 3.17 | A0A5C7HR14; A0A2S1KMY6; A0A5C7GUZ6; A0A5C7H156; A0A5C7H1I2; A0A5C7INT9; A0A5C7IP03; A0A5C7J0Q1; A0A6B9S303 | Tubulin beta chain |  | At5g62700 | 388; 388; 388; 388; 385; 388; 388; 388; 388 | 1 | 0.0261423 | UID12 | 97.431 | SIQEMFRRVSEQFTAMFRRKAFLHWYTGEGM | 1 |
| 0.0033 | 3.14 | A0A5C7HAY7 | Glutathione transferase | Elongation factor 1-gamma | At1g09640 | 165 | 1 | 2.67839E-35 | UID122 | 58.024 | SLEIDANIRLWYGPRMGFAVHLPPAEEAAIS | 1 |
| 0.00019 | 3.13 | A0A5C7HQB5 | Condensin complex subunit 1 C-terminal domain-containing protein |  | At2g33540 | 906 | 1 | 0.0371443 | UID184 | 87.616 | LSVRLRNLQICNNIKMRADAFAAFGALSNYG | 1 |
| 0.0033 | 3.13 | A0A5C7HQY3 | CTLH domain-containing protein |  | At5g66810 | 600 | 1 | 0.036639 | UID567 | 56.563 | QVAIGRRLGIEEPQLMKIMRAALHTHNEWFK | 2 |
| 0.00086 | 3.07 | A0A5C7I2L5 | Glutathione transferase | Glutathione S-transferase F6 | At1g02930 | 36 | 1 | 5.84871E-10 | UID234 | 88.191 | CLYEKDIDFELVPVDMSAGEHKSEAFLAKNP | 1 |
| 0.00014 | 3.04 | A0A5C7ITA3 | SMP domain-containing protein | Late embryogenesis abundant protein D-34 | At4g26080 | 266 | 1 | 1.53189E-05 | UID342 | 52.061 | NSPNLTTHPGGIGSSMDTAARLNEDDDDLR_ | 1 |
| 0.000058 | 3.03 | A0A5C7HSE7 | Large ribosomal subunit protein uL2 C-terminal domain-containing protein |  | At3g45470 | 97 | 1 | 0.00363881 | UID196 | 91.752 | IYTGQFIYCGKKATLMVGNVLPVRSIPEGAV | 1 |
| 0.0052 | 3.03 | A0A5C7I486; A0A5C7GU81; A0A5C7IT33 | 60S ribosomal protein L13a |  | At3g07110 | 67; 68; 67 | 1 | 0.00175478 | UID243 | 121.36 | LVRQKMKYMRFLRKRMNTKPSHGPIHFRAPA | 1 |
| 0.000046 | 2.99 | A0A5C7HQL8 | Large ribosomal subunit protein uL4 C-terminal domain-containing protein |  | At5g02040 | 17 | 1 | 7.15045E-43 | UID186 | 161.68 | AAARPLVTVQSLEGDMATDATPTVALPDVMK | 1 |
| 0.011 | 2.91 | A0A5C7INQ6 | Uncharacterized protein | low-temperature-induced 65 kDa protein-like | At5g52300 | 152 | 1 | 7.40568E-22 | UID317 | 78.588 | QEGTKGQHKVTLGSVMGEDVTHASQNKPVTH | 1 |
| 0.00014 | 2.8 | A0A5C7H329; A0A5C7H4G9 | Malate synthase |  | At5g03860 | 116; 119 | 1 | 0.021275 | UID71 | 94.692 | VADRRVEITGPVDRKMVINALNSGAKVFMAD;VADRRVEITGPVERKMVINALNSGAKVFMAD | 1 |
| 0.00085 | 2.8 | A0A5C7IUE7 | Large ribosomal subunit protein uL18 C-terminal eukaryotes domain-containing protein |  | At5g37760 | 211 | 1 | 1.32531E-08 | UID345 | 49.34 | KYIFGGHVAAYMRTLMEDEPEKYQSHFCEFI | 1 |
| 0.00025 | 2.77 | A0A5C7IIR0 | Tr-type G domain-containing protein | Elongation factor 2 | At1g56070 | 833 | 1 | 1.50244E-06 | UID288 | 130.24 | TLVADIRKRKGLKEQMTPLSDFEDKL_____ | 1 |
| 0.0019 | 2.74 | A0A5C7IWE5 | Poly [ADP-ribose] polymerase |  | At2g31320 | 683 | 1 | 0.00578737 | UID357 | 74.611 | KGFLPAVCSLPVPGYMFGKAIVCSDAVAEAA | 1 |
| 0.00013 | 2.72 | A0A5C7HB18 | Uncharacterized protein | Heat shock 70 kDa protein. mitochondrial | At4g37910 | 141 | 1 | 0.00201893 | UID124 | 139.31 | RLIGRRFDDPQTQKEMKMVPYKIVRAPNGDA | 1 |
| 0.0014 | 2.68 | A0A5C7IX98; A0A6B9S3G2 | Elongation factor 1-alpha |  | At1g07940 | 264; 264 | 1 | 2.00565E-09 | UID363 | 90.978 | TVPVGRVETGIIKPGMVVTFGPTGLTTEVKS | 1 |
| 0.00085 | 2.67 | A0A5C7IN17 | Small ribosomal subunit protein uS10 |  | At3g13120 | 7 | 1 | 0.0314763 | UID312 | 48.761 | _________MAAYVAMKGKPGLEEPQEQIHK | 1 |
| 0.002 | 2.67 | A0A5C7HV96 | Uncharacterized protein | Heat shock 70 kDa protein | At3g09440 | 88 | 1 | 3.89231E-05 | UID205 | 118.4 | RLIGRRFSDPSVQSDMKLWPFKVIPGPGDKP | 1 |
| 0.0089 | 2.66 | A0A5C7HJH0 | phosphoglucomutase (alpha-D-glucose-1.6-bisphosphate-dependent) |  | At1g70730 | 32 | 1 | 0.00118511 | UID156 | 63.184 | QKPGTSGLRKKVKVFMQPHYLQNFVQSTFNA | 1 |
| 0.000069 | 2.64 | A0A5C7HRJ0 | Uncharacterized protein | Late embryogenesis abundant protein LEA_5 subgroup | At3g51810 | 179 | 1 | 1.489E-20 | UID192 | 61.641 | GQTRKDQLGHEGYQEMGQRGGQTRKDQLSHE | 1 |
| 0.000094 | 2.6 | A0A5C7H8Z0; A0A5C7HKG2; A0A5C7HT26;A0A6B9S1H9; A0A5C7I489; A0A5C7IX25 | Tubulin alpha chain |  | At1g64740 | 318; 318; 318; 318; 318; 306 | 1 | 0.026514 | UID107 | 105.12 | AKCDPRHGKYMACCLMYRGDVVPKDVNAAVA | 1 |
| 0.00091 | 2.58 | A0A5C7HCS3 | Proteasome subunit beta | Proteasome subunit beta type-7-B | At5g40580 | 97 | 0.999998 | 1.8141E-10 | UID128 | 73.548 | CCGAGTAADTEAVTDMVSSQLQLHRYHTGRE | 1 |
| 0.0038 | 2.58 | A0A5C7HQY3 | CTLH domain-containing protein |  | At5g66810 | 603 | 1 | 0.036639 | UID568 | 56.563 | IGRRLGIEEPQLMKIMRAALHTHNEWFKLQM | 2 |
| 0.0043 | 2.52 | A0A5C7H151; A0A5C7IFK6 | Histidine kinase/HSP90-like ATPase domain-containing protein |  | At5g56030 | 450; 448 | 1 | 2.44711E-29 | UID62 | 120.96 | FAELLRYYSTKSGDEMTSLKDYVTRMKEGQK;LAELLRYHSTKSGDEMTSLKDYVTRMKEGQS | 1 |
| 0.00021 | 2.47 | A0A5C7ILC7 | Glutamine synthetase |  | At5g35630 | 373 | 1 | 0.00619844 | UID310 | 83.499 | KEGKGYFEDRRPASNMDPYVVTSMIAETTLL | 1 |
| 0.0017 | 2.43 | A0A5C7HHZ0 | DYW domain-containing protein | Putative pentatricopeptide repeat-containing protein At5g40405 | At5g40405 | 1 | 1 | 0.00455829 | UID154 | 49.418 | _______________MSSLRCTITKNPIIFL | 1 |
| 0.00017 | 2.42 | A0A5C7HDY5 | Phospholipase A1 |  | At4g18550 | 85 | 1 | 0.00352248 | UID137 | 45.864 | KGRVQSYADKARSGNMVGSREEHEEKTRGPH | 1 |
| 0.00014 | 2.41 | A0A5C7HYK7 | SHSP domain-containing protein | 17.6 kDa class II heat shock protein | At5g12020 | 6 | 0.998156 | 2.35189E-21 | UID225 | 115.55 | __________MDFRIMGMDSPLFSTLQHMMD | 1 |
| 0.0053 | 2.41 | A0A5C7GT03 | 40S ribosomal protein S14 |  | At2g36160 | 74 | 1 | 5.84326E-08 | UID30 | 113.73 | MKVKADRDESSPYAAMLAAQDVSTRCKELGI | 1 |
| 0.00034 | 2.39 | A0A5C7HR14; A0A2S1KMY6; A0A5C7H156; A0A5C7H1I2; A0A5C7INT9; A0A5C7IP03; A0A5C7J0Q1; A0A6B9S303 | Tubulin beta chain |  | At1g75780 | 66; 66; 66; 66; 66; 66; 66; 66 | 0.999595 | 0.0020871 | UID8 | 90.146 | YNEASCGRFVPRAVLMDLEPGTMDSVRSGPY;YNEASCGRFVPRAVLMDLEPGTMDSVRSGTY;YNEASCGRFVPRAVLMDLEPGTMDSVRTGPY;YNEASNGRYVPRAVLMDLEPGTMDSVRTGPY;YNEASTGRYVPRAVLMDLEPGTMDSVRSGPY | 1 |
| 0.00032 | 2.38 | A0A5C7IIR0 | Tr-type G domain-containing protein | Elongation factor 2 | At1g56070 | 759 | 1 | 0.00398275 | UID292 | 68.525 | IYSVLNQKRGHVFEEMQRQGTPLYNIKAYLP | 1 |
| 0.00037 | 2.33 | A0A5C7HXN9; A0A5C7HYD1; A0A5C7HYH5; A0A5C7IJ29 | ATP-dependent RNA helicase | Eukaryotic initiation factor 4A | At1g54270 | 185; 185; 185; 257 | 1 | 0.00382994 | UID222 | 110.86 | FDMLRRQSLRADHIKMFVLDEADEMLSRGFK;FDMLRRQSLRPDHIKMFVLDEADEMLSRGFK;FDMLRRQSLRPDYIKMFVLDEADEMLSRGFK;FDMLRRQSLRSDNIKMFVLDEADEMLSRGFK | 1 |
| 0.00087 | 2.29 | A0A5C7IQ10 | Succinate-semialdehyde dehydrogenase. mitochondrial |  | At1g79440 | 208 | 1 | 1.33131E-16 | UID330 | 106.19 | VGVVGAITPWNFPLAMITRKVGPALACGCTV | 1 |
| 0.001 | 2.28 | A0A5C7GSQ8; A0A5C7HDG9 | DUF295 domain-containing protein | Glyoxylate/hydroxypyruvate reductase | At1g79870 | 87; 121 | 0.959149 | 1.83436E-05 | UID26 | 119.27 | FKKFVVHNAKELELLMMHNRYKSLIHKCALS | 1 |
| 0.00031 | 2.27 | A0A5C7GS21; A0A5C7GPK8; A0A5C7GSL2; A0A5C7HDS1 | Acetohydroxy-acid reductoisomerase |  | At3g58610 | 540; 313; 295; 502 | 1 | 0.00369534 | UID22 | 96.604 | VDSLNPFMHARGVSFMVDNCSTTARLGSRKW | 1 |
| 0.00051 | 2.27 | A0A5C7H6K2 | Histidine kinase/HSP90-like ATPase domain-containing protein |  | At5g52640 | 459 | 1 | 0.0386065 | UID86 | 92.062 | GLVDSDTLPLNVSREMLQQHSSLKTIKKKLI | 1 |
| 0.00036 | 2.25 | A0A5C7IU90 | Serine hydroxymethyltransferase |  | At4g37930 | 484 | 0.936203 | 0.0174197 | UID344 | 88.803 | DVEKFSGTFDMPGFAMSEMKYKD________ |  |
| 0.00051 | 2.24 | A0A5C7I476 | Ribosomal protein L14b/L23e |  | At1g17560 | 62 | 0.946969 | 0.00906644 | UID241 | 87.216 | GRLNRLPSACVGDMVMATVKKGKPDLRKKVM | 1 |
| 0.00046 | 2.22 | A0A5C7HGB1 | Dienelactone hydrolase domain-containing protein |  | At3g25100 | 124 | 1 | 0.000109657 | UID148 | 81.12 | YRGKVGLDVAEAQHLMDGLDWQGAVKDIRAS | 1 |
| 0.0011 | 2.21 | A0A5C7IJK9 | Ig-like domain-containing protein | Heat shock 70 kDa protein 14 | At1g79930 | 58 | 0.936994 | 0.000364196 | UID301 | 120.96 | DKQRFIGTAGAATSMMNPKNTISQIKRLIGR | 1 |
| 0.0023 | 2.2 | A0A5C7I2L5 | Glutathione transferase | Glutathione S-transferase F6 | At1g02930 | 126 | 0.999988 | 3.25098E-15 | UID238 | 145.6 | ASKLNWEIVFKPMFGMTTDPAAVEELEAKLS | 1 |
| 0.00031 | 2.19 | A0A5C7GRT8 | Uncharacterized protein | Late embryogenesis abundant protein 14-like | At5g17165 | 235 | 1 | 0.00568484 | UID20 | 144.55 | ETVVGKDDKDDREKKMDGDVEEMRRRAGERD | 1 |
| 0.0002 | 2.12 | A0A5C7I6D2 | Peptidylprolyl isomerase |  | At3g25230 | 22 | 1 | 0.000186915 | UID255 | 109.42 | FFDLTIGGAPAGRVVMELYADTTPKTAENFR | 1 |
| 0.0011 | 2.12 | A0A5C7GWM8 | 60S ribosomal protein L21 |  | At1g57860 | 114 | 1 | 4.9686E-21 | UID32 | 128.43 | KISTKRQPEGPKPGFMVEGATLETVTPIPYD | 1 |
| 0.00097 | 2.11 | A0A5C7I2I3; A0A5C7I4Y6 | MPN domain-containing protein | NPL4-like protein 1 | At3g63000 | 206; 206 | 0.999958 | 0.000216376 | UID233 | 70.783 | TEEEKMVDAIAAGLGMKKVGFIFTQTIMQNK | 1 |
| 0.0031 | 2.11 | A0A5C7HV96 | Uncharacterized protein | Heat shock 70 kDa protein | At3g09440 | 241 | 1 | 0.0144036 | UID206 | 104.2 | TAGDTHLGGEDFDNRMVNHFVSEFRRKHKKD | 1 |
| 0.00033 | 2.1 | A0A5C7I2F0 | Cupin type-1 domain-containing protein | Vicilin-like protein | At3g22640 | 402 | 1 | 3.30765E-76 | UID232 | 68.83 | ATKIAVVTDGDGYFEMACPHVSKSSDDEQQQ | 1 |
| 0.023 | 2.09 | A0A5C7IQT7 | Dihydrolipoyl dehydrogenase |  | At3g16950 | 471 | 1 | 1.33092E-21 | UID333 | 42.659 | HLSIPAACFTHPEISMVGLTEPQAREKGEKE | 1 |
| 0.001 | 2.08 | A0A5C7GRT8 | Uncharacterized protein | Late embryogenesis abundant protein 14-like | At5g17165 | 242 | 1 | 0.00845348 | UID21 | 121.36 | DKDDREKKMDGDVEEMRRRAGERDEKKNNKY | 1 |
| 0.003 | 2.04 | A0A5C7HPZ7 | Peptidase M24 domain-containing protein | ERBB-3 BINDING PROTEIN 1 | At3g51800 | 429 | 1 | 0.0463826 | UID183 | 68.44 | ASRFIFSEISQNFPIMPFTARALEEKRARLG | 1 |
| 0.026 | 2.03 | A0A5C7IIR0; A0A5C7IRX9 | Tr-type G domain-containing protein | Elongation factor 2 | At1g56070 | 650; 650 | 0.999885 | 4.60922E-28 | UID289 | 119.01 | WCFGPETTGPNMVVDMCKGVQYLNEIKDSVV | 1 |
| 0.00043 | 2 | A0A5C7HNF5 | Monodehydroascorbate reductase (NADH) |  | At1g63940 | 202 | 1 | 8.63817E-26 | UID173 | 177.54 | VGGGYIGLELSAALRMNDIDVSMVYPEPWCM | 1 |

| **Log_2_FC** | **adj P Val** | **Proteins** | **Protein names** | **Explicated protein name**** | **Gene*** | **Positions within proteins** | **Localization prob** | **PEP** | **Unique_identifier** | **Score for localization** | **Sequence window** | **Nr of Oxidation M** |
| --- | --- | --- | --- | --- | --- | --- | --- | --- | --- | --- | --- | --- |
| 0.00064 | -2.04 | A0A5C7ID52; A0A5C7IWK4 | 5-methyltetrahydropteroyltriglutamate-homocysteine S-methyltransferase |  | At3g03780 | 557; 601 | 1 | 9.28341E-17 | UID269 | 123.84 | WSSMAQSMTARPMKGMLTGPVTILNWSFVRN; WSSMAQSMTKRPMKGMLTGPVTILNWSFVRN | 1 |
| 0.00027 | -2.11 | A0A5C7HPI8 | Gibberellin-regulated protein 14 |  | At5g14920 | 240 | 1 | 0.00474652 | UID181 | 108.67 | GTYGNREKCGKCYTEMTTHGNKPKCP_____ | 1 |
| 0.00041 | -2.22 | A0A5C7I6D2 | Peptidylprolyl isomerase |  | At3g25230 | 107 | 1 | 2.56704E-55 | UID253 | 52.721 | DENFTKKHTEAGILSMANAGPGTNGSQFFIC | 1 |
| 0.015 | -2.25 | A0A5C7IPK3 | Phosphoglycerate kinase |  | At3g12780 | 176 | 1 | 1.94437E-06 | UID324 | 62.732 | EGVTKYLKPSVAGFLMQKELDYLVGAVANPK | 1 |
| 0.00044 | -2.3 | A0A5C7IAE2 | Glycosyltransferase | UDP-glycosyltransferase 87A2 | At2g30140 | 285 | 1 | 0.00614506 | UID262 | 63.488 | KWLNSKPTSSVLYVSMGSLFKVSSAQLDELV | 1 |
| 0.00053 | -2.3 | A0A7L8XKU1 | Large ribosomal subunit protein uL23c |  | AtCg00840 | 66 | 0.988374 | 0.0340636 | UID380 | 59.245 | NSHQLPRKGRRMGPIMGHTMHYRRMIITLQP | 2 |
| 0.0013 | -2.33 | A0A5C7IFM5 | SUI1 domain-containing protein | Protein translation factor sui1 homolog 2 | At1g54290 | 129 | 1 | 6.76807E-32 | UID1036 | 44.258 | QQKRQIIEIAESIPSMCNMRPMECDFESSRS | 1; 2; 3 |
| 0.0026 | -2.47 | A0A5C7IFM5 | SUI1 domain-containing protein | Protein translation factor sui1 homolog 2 | At1g54290 | 132 | 1 | 2.55352E-42 | UID1037 | 44.258 | RQIIEIAESIPSMCNMRPMECDFESSRSDMS | 1; 2; 3 |
| 0.00007 | -2.75 | A0A5C7HEV3 | Beta-amylase |  | At3g23920 | 499 | 1 | 0.0338574 | UID144 | 116.54 | ARPNGINKNGPPKLRMYGVTYLRLSDDLLEE | 1 |
| 0.042 | -2.79 | A0A5C7GZ05; A0A5C7GZ31; A0A5C7HNB7; A0A5C7IXL3 | Uncharacterize dprotein | Heat shock cognate 70 kDa protein | At5g02490 | 243; 244; 243; 243 | 1 | 0.00320703 | UID38 | 114.4 | TAGDTHLGGEDFDNRMVNHFVQEFKRKHKKD;TAGDTHLGGEDFDNRMVNHFVQEFKRKNKKD | 1 |
| 0.00053 | -2.83 | A0A5C7GZ64 | Ferritin |  | At5g01600 | 257 | 1 | 0.00404175 | UID48 | 64.454 | LRRVGKGHGVWHFDQMLLNEEAVVA______ | 1 |
| 0.00055 | -2.83 | A0A5C7H0R5 | SHSP domain-containing protein | 18.1 kDa class I heat shock protein | At5g59720 | 132 | 1 | 0.000730931 | UID60 | 124.42 | FRLPENARVDQIKACMENGVLTVNVPKMEEK | 1 |
| 0.000069 | -2.92 | A0A7L8XKU1 | Large ribosomal subunit protein uL23c |  | AtCg00840 | 62 | 0.988374 | 0.0340636 | UID379 | 59.245 | VIAMNSHQLPRKGRRMGPIMGHTMHYRRMII | 2 |
| 0.000063 | -3.01 | A0A5C7I4H9 | glutathione transferase | glutathione S-transferase F13 | At3g62760 | 10 | 1 | 0.0189552 | UID248 | 82.287 | ______MALKLYGAPMSTCTTRVTISLYEKD | 1 |
| 0.000063 | -3.13 | A0A5C7IEE3 | DJ-1/PfpI domain-containing protein | protein DJ-1 homolog B | At1g53280 | 329 | 1 | 4.04388E-07 | UID275 | 98.439 | WGLLKGLKATCYPSFMEQLAPACATTVESRV | 1 |
| 0.00037 | -3.15 | A0A5C7IVC4 | Protein kinase domain-containing protein | serine/threonine-protein kinase Nek6 | At3g20860 | 10 | 0.999972 | 0.0203554 | UID349 | 53.751 | ______MEEIKSDDQMRSKMDDYQEIEQIGR | 1 |
| 0.000079 | -3.36 | A0A5C7H6K2 | Histidine kinase/HSP90-like ATPase domain-containing protein |  | At5g52640 | 685 | 1 | 0.0123585 | UID87 | 118.74 | TPCVVVTSKYGWSANMERIMQSQTLSDANKQ | 1 |
| 0.000028 | -3.48 | A0A5C7GXS3 | 40S ribosomal protein S17 |  | At1g79850 | 82 | 1 | 0.00199472 | UID35 | 133.91 | RGISLKLQEEERERRMDFVPDESAIRIDEIK | 1 |
| 0.000024 | -3.58 | A0A5C7H8G1 | Peroxidase | Peroxidase 4 | At1g14540 | 316 | 1 | 1.29704E-29 | UID481 | 144.1 | LFFDQFALSMIKMGQMNVLTGTLGEVRANCS | 1; 2 |
| 0.000012 | -3.72 | A0A5C7HSM7 | KOW domain-containing protein | 60S ribosomal protein L26-1 | At3g49910 | 243 | 1 | 0.0018436 | UID197 | 61.423 | RKAHFTAPSSVRRILMSAPLSTDLRQKYNVR | 1 |
| 0.000028 | -3.73 | A0A5C7H8G1 | Peroxidase | Peroxidase 4 | At1g14540 | 313 | 1 | 1.77841E-51 | UID480 | 144.1 | DEKLFFDQFALSMIKMGQMNVLTGTLGEVRA | 1; 2 |
| 0.000063 | -3.89 | A0A5C7IAQ5 | DUF1639 domain-containing protein | Chromogranin | At4g20480 | 191 | 1 | 0.0384399 | UID263 | 44.567 | QRSLLLVSPGAWLTDMCQERYEVREKKSSKK | 1 |
| 0.000052 | -4 | A0A5C7IJX8 | Phytocyanin domain-containing protein | Stellacyanin | At5g20230 | 12 | 1 | 0.00017635 | UID1061 | 73.466 | ____MEKMLVVLVVVMAAISCLGGKLAHAQL | 3 |
| 0.000087 | -4.01 | A0A5C7GSF3 | Glutamate receptor |  | At3g04110 | 568 | 1 | 0.038226 | UID401 | 41.429 | GLSMIVPAKSEESAWMFMRPFTWKMWVVTGV | 2 |
| 0.000061 | -4.06 | A0A5C7GSF3 | Glutamate receptor |  | At3g04110 | 570 | 1 | 0.038226 | UID402 | 41.429 | SMIVPAKSEESAWMFMRPFTWKMWVVTGVIL | 2 |
| 0.000028 | -4.18 | A0A5C7H0C0 | Elongation factor Tu. chloroplastic |  | At4g20360 | 111 | 0.999999 | 3.0231E-170 | UID58 | 129.11 | GKTTLTAALTMALAAMGNSAPKKYDEIDAAP | 1 |
| 0.000079 | -4.23 | A0A5C7IJX8 | Phytocyanin domain-containing protein | Stellacyanin | At5g20230 | 1 | 1 | 0.00017635 | UID1059 | 73.466 | _______________MEKMLVVLVVVMAAIS | 3 |
| 0.0000082 | -4.35 | A0A5C7IFM5 | SUI1 domain-containing protein | Protein translation factor sui1 homolog 2 | At1g54290 | 129 | 1 | 6.76807E-32 | UID658 | 44.258 | QQKRQIIEIAESIPSMCNMRPMECDFESSRS | 1; 2; 3 |
| 0.000017 | -4.48 | A0A5C7I8S3 | Uncharacterize dprotein | NADPH-dependent aldehyde reductase 1. chloroplastic | At1g54870 | 25 | 1 | 0.0100183 | UID258 | 60.528 | FPPQKQHAQPGKEHEMDPSPQFAKQDYTPSN | 1 |
| 0.000012 | -4.5 | A0A5C7IFM5 | SUI1 domain-containing protein | Protein translation factor sui1 homolog 2 | At1g54290 | 135 | 1 | 9.51649E-43 | UID660 | 44.258 | IEIAESIPSMCNMRPMECDFESSRSDMSRQW | 1; 2; 3 |
| 0.000017 | -4.59 | A0A5C7IJX8 | Phytocyanin domain-containing protein | stellacyanin | At5g20230 | 4 | 1 | 0.00017635 | UID1060 | 73.466 | ____________MEKMLVVLVVVMAAISCLG | 3 |
| 0.0000064 | -4.68 | A0A5C7IEE3 | DJ-1/PfpI domain-containing protein | protein DJ-1 homolog B | At1g53280 | 385 | 1 | 8.25299E-07 | UID274 | 101.97 | LYGKEKADEVAGPLVMRSNHGDEYTITEFSP | 1 |
| 0.0000064 | -4.7 | A0A5C7H3K9 | Glyceraldehyde-3-phosphate dehydrogenase |  | At3g04120 | 329 | 1 | 0.00124279 | UID78 | 52.49 | NEWGYSSRVVDLIVHMAKSQ___________ | 1 |
| 0.0000056 | -4.84 | A0A5C7I2L5 | Glutathione transferase | Glutathione S-transferase F6 | At1g02930 | 123 | 0.978174 | 5.13865E-11 | UID237 | 102.69 | DPVASKLNWEIVFKPMFGMTTDPAAVEELEA | 1 |
| 0.000012 | -4.94 | A0A5C7IFM5 | SUI1 domain-containing protein | Protein translation factor sui1 homolog 2 | At1g54290 | 132 | 1 | 2.55352E-42 | UID659 | 44.258 | RQIIEIAESIPSMCNMRPMECDFESSRSDMS | 1; 2; 3 |
| 0.0000056 | -5.07 | A0A5C7H8G1 | Peroxidase | Peroxidase 4 | At1g14540 | 313 | 1 | 1.77841E-51 | UID102 | 144.1 | DEKLFFDQFALSMIKMGQMNVLTGTLGEVRA | 1; 2 |
| 0.0000064 | -5.15 | A0A5C7H8G1 | Peroxidase | Peroxidase 4 | At1g14540 | 316 | 1 | 1.29704E-29 | UID103 | 144.1 | LFFDQFALSMIKMGQMNVLTGTLGEVRANCS | 1; 2 |
| 0.0000045 | -5.44 | A0A5C7HHV4 | DUF4283 domain-containing protein |  | At1g36960 | 1 | 1 | 0.020948 | UID153 | 54.549 | _______________MNAEDIERLCGYLSLE | 1 |
| 0.000011 | -5.92 | A0A5C7IFM5 | SUI1 domain-containing protein | Protein translation factor sui1 homolog 2 | At1g54290 | 129 | 1 | 6.76807E-32 | UID280 | 44.258 | QQKRQIIEIAESIPSMCNMRPMECDFESSRS | 1; 2; 3 |
| 0.0000033 | -6.39 | A0A5C7H9X2 | AAA+ ATPase domain-containing protein | Protein STICHEL | At2g02480 | 1366 | 1 | 3.38292E-17 | UID117 | 80.69 | NPESRSESLQQCCSQMHKLDDKCHCKALKMM | 1 |
