# Supplementary material for: Proteomics- and metabolomics-based analysis of the regulation of germination in Norway maple and sycamore embryonic axes
Source: Tree Physiol. 2025 Jan 6;45(2):tpaf003. doi: 10.1093/treephys/tpaf003 (PMC11791354; doi:10.1093/treephys/tpaf003)
Supplement: Table_S11_tpaf003 [file table_s11_tpaf003.docx]

**Table S8B.** Functional analysis of proteins containing MetO significantly regulated in embryonic axes of sycamore seeds at the imbibed stage compared to Norway maple and in terms of molecular function, biological process, cellular compartment and protein class based on Gene Ontology annotation (PANTHER). Child categories are presented for each category. GO terms and numbers (given in brackets) according to PANTHER classification system are followed the number of genes in each category.

|  | **Upregulated in sycamore** | **Downregulated in sycamore** |
| --- | --- | --- |
| **MOLECULAR FUNCTION** | **binding (GO:0005488) 9**  small molecule binding (GO:0036094) 4  ion binding (GO:0043167) 4  organic cyclic compound binding (GO:0097159) 4  amide binding (GO:0033218) 2  protein binding (GO:0005515) 2  carbohydrate derivative binding (GO:0097367) 1  **catalytic activity (GO:0003824) 8**  transferase activity (GO:0016740) 5  catalytic activity, acting on a protein (GO:0140096) 2  catalytic activity, acting on a nucleic acid (GO:0140640) 1  isomerase activity (GO:0016853) 1  oxidoreductase activity (GO:0016491) 1  **structural molecule activity (GO:0005198) 3**  structural constituent of ribosome (GO:0003735) 3  **ATP-dependent activity (GO:0140657) 1**  ATP-dependent activity, acting on DNA (GO:0008094) 1  **molecular transducer activity (GO:0060089) 1**  signaling receptor activity (GO:0038023) 1  **translation regulator activity (GO:0045182) 1**  translation regulator activity, nucleic acid binding (GO:0090079) 1  **transporter activity (GO:0005215) 1**  transmembrane transporter activity (GO:0022857) 1 | **catalytic activity (GO:0003824) 30**  oxidoreductase activity (GO:0016491) 9  transferase activity (GO:0016740) 8  hydrolase activity (GO:0016787) 8  catalytic activity, acting on a protein (GO:0140096) 6  isomerase activity (GO:0016853) 3  ligase activity (GO:0016874) 2  lyase activity (GO:0016829) 1  **binding (GO:0005488) 26**  organic cyclic compound binding (GO:0097159) 17  small molecule binding (GO:0036094) 9  ion binding (GO:0043167) 5  protein binding (GO:0005515) 6  carbohydrate derivative binding (GO:0097367) 4  protein-containing complex binding (GO:0044877) 2  amide binding (GO:0033218) 1  lipid binding (GO:0008289) 1  **structural molecule activity (GO:0005198) 11**  structural constituent of ribosome (GO:0003735) 7  structural constituent of cytoskeleton (GO:0005200) 4  **translation regulator activity (GO:0045182) 6**  translation regulator activity, nucleic acid binding (GO:0090079) 6  **transporter activity (GO:0005215) 1**  transmembrane transporter activity (GO:0022857) 1  **antioxidant activity (GO:0016209) 1**  no hit |
| **BIOLOGICAL PROCESS** | **cellular process (GO:0009987) 13**  cellular metabolic process (GO:0044237) 10  cellular nitrogen compound metabolic process (GO:0034641) 5  cellular component organization or biogenesis (GO:0071840) 3  protein folding (GO:0006457) 3  cellular response to stimulus (GO:0051716) 2  maintenance of location in cell (GO:0051651) 1  microtubule-based process (GO:0007017) 1  cellular localization (GO:0051641) 1  **metabolic process (GO:0008152) 10**  cellular metabolic process (GO:0044237) 10  nitrogen compound metabolic process (GO:0006807) 10  organic substance metabolic process (GO:0071704) 10  primary metabolic process (GO:0044238) 8  biosynthetic process (GO:0009058) 6  catabolic process (GO:0009056) 2  small molecule metabolic process (GO:0044281) 2  **response to stimulus (GO:0050896) 3**  response to stress (GO:0006950) 3  cellular response to stimulus (GO:0051716) 2  response to abiotic stimulus (GO:0009628) 2  response to chemical (GO:0042221) 1  **biological regulation (GO:0065007) 1**  regulation of biological quality (GO:0065008) 1  **homeostatic process (GO:0042592) 1**  cellular homeostasis (GO:0019725) 1  chemical homeostasis (GO:0048878) 1  **localization (GO:0051179) 1**  cellular localization (GO:0051641) 1  maintenance of location (GO:0051235) 1 | **metabolic process (GO:0008152) 36**  organic substance metabolic process (GO:0071704) 35  primary metabolic process (GO:0044238) 32  nitrogen compound metabolic process (GO:0006807) 31  cellular metabolic process (GO:0044237) 29  biosynthetic process (GO:0009058) 17  small molecule metabolic process (GO:0044281) 13  catabolic process (GO:0009056) 9  **cellular process (GO:0009987) 34**  cellular metabolic process (GO:0044237) 29  cellular component organization or biogenesis (GO:0071840) 8  protein folding (GO:0006457) 6  cell cycle (GO:0007049) 5  cellular response to stimulus (GO:0051716) 5  microtubule-based process (GO:0007017) 4  cell cycle process (GO:0022402) 1  **response to stimulus (GO:0050896) 11**  response to stress (GO:0006950) 9  cellular response to stimulus (GO:0051716) 5  response to abiotic stimulus (GO:0009628) 5  response to chemical (GO:0042221) 5  response to endogenous stimulus (GO:0009719) 1  **biological regulation (GO:0065007) 4**  regulation of biological process (GO:0050789) 2  regulation of biological quality (GO:0065008) 2  **detoxification (GO:0098754) 1**  no hit  **homeostatic process (GO:0042592) 1**  cellular homeostasis (GO:0019725) 1 |
| **CELLULAR COMPONENT** | **cellular anatomical entity (GO:0110165) 17**  intracellular anatomical structure (GO:0005622) 15  cytoplasm (GO:0005737) 14  cytosol (GO:0005829) 6  organelle (GO:0043226) 6  cell periphery (GO:0071944) 4  membrane (GO:0016020) 3  perinuclear region of cytoplasm (GO:0048471) 1  replication fork (GO:0005657) 1  supramolecular complex (GO:0099080) 1  **protein-containing complex (GO:0032991) 4**  ribonucleoprotein complex (GO:1990904) 3 | **cellular anatomical entity (GO:0110165) 49**  external encapsulating structure (GO:0030312) 1  extracellular region (GO:0005576) 2  intracellular anatomical structure (GO:0005622) 46  cytoplasm (GO:0005737) 41  organelle (GO:0043226) 25  cytosol (GO:0005829) 22  membrane (GO:0016020) 5  supramolecular complex (GO:0099080) 5  cell periphery (GO:0071944) 4  membrane-enclosed lumen (GO:0031974) 3  perinuclear region of cytoplasm (GO:0048471) 2  nucleoplasm (GO:0005654) 1  cell junction (GO:0030054) 1  envelope (GO:0031975) 1  **protein-containing complex (GO:0032991) 14**  ribonucleoprotein complex (GO:1990904) 9  catalytic complex (GO:1902494) 4  intracellular protein-containing complex (GO:0140535) 3  mitochondrial protein-containing complex (GO:0098798) 2  membrane protein complex (GO:0098796) 1  nuclear protein-containing complex (GO:0140513) 1  proteasome accessory complex (GO:0022624) 1  proteasome regulatory particle (GO:0005838) 1  proteasome regulatory particle, lid subcomplex (GO:0008541) 1  protein-DNA complex (GO:0032993) 1 |
| **PROTEIN CLASS** | **metabolite interconversion enzyme (PC00262) 8**  transferase (PC00220) 5  oxidoreductase (PC00176) 3  **translational protein (PC00263) 5**  ribosomal protein (PC00202) 3  translation factor (PC00223) 2  **chaperone (PC00072) 4**  Hsp70 family chaperone (PC00027) 1  Hsp90 family chaperone (PC00028) 1  **storage protein (PC00210) 1**  no hit  **protein modifying enzyme (PC00260) 1**  non-receptor serine/threonine protein kinase (PC00167) 1  **DNA metabolism protein (PC00009) 1**  DNA-directed DNA polymerase (PC00018) 1  **RNA metabolism protein (PC00031) 1**  no hit  **transmembrane signal receptor (PC00197) 1**  no hit | **metabolite interconversion enzyme (PC00262) 21**  oxidoreductase (PC00176) 12  transferase (PC00220) 5  isomerase (PC00135) 2  hydrolase (PC00121) 1  ligase (PC00142) 1  **translational protein (PC00263) 13**  ribosomal protein (PC00202) 8  translation factor (PC00223) 5  **chaperone (PC00072) 9**  Hsp70 family chaperone (PC00027) 4  Hsp90 family chaperone (PC00028) 2  **protein modifying enzyme (PC00260) 6**  protease (PC00190) 3  protein phosphatase (PC00195) 2  ubiquitin-protein ligase (PC00234) 1  **cytoskeletal protein (PC00085) 4**  microtubule or microtubule-binding cytoskeletal protein (PC00157) 4  **DNA metabolism protein (PC00009) 2**  replication origin binding protein (PC00199) 1  **RNA metabolism protein (PC00031) 2**  RNA helicase (PC00032) 1  **transporter (PC00227) 2**  primary active transporter (PC00068) 1  secondary carrier transporter (PC00258) 1  **calcium-binding protein (PC00060) 1**  no hit  **scaffold/adaptor protein (PC00226) 1**  no hit |
