# Supplementary material for: Proteomics- and metabolomics-based analysis of the regulation of germination in Norway maple and sycamore embryonic axes
Source: Tree Physiol. 2025 Jan 6;45(2):tpaf003. doi: 10.1093/treephys/tpaf003 (PMC11791354; doi:10.1093/treephys/tpaf003)
Supplement: Table_S13_tpaf003 [file table_s13_tpaf003.docx]

**Table S13.** The list of identified proteins containing methionine sulfoxide (MetO) in our study with calculated changes in abundance assigned as upregulated (log_2_FC>2) and downregulated (log_2_FC<–2) in germinated Norway maple seeds as compared to sycamore seeds. Protein name was derived from UniProt database (UniProt Consortium 2021) accessed on February 2024. * Gene abbreviation refers to homological *Arabidopsis thaliana* gene recognized by protein-coding gene classification information knowledgebases. **Amino acid sequences of proteins assigned as uncharacterized, containing a specific domain or identified only to class were extracted from UniProt database and were explicated using PSI-BLAST search method (Bhagwat and Aravind 2007). Green color font of a gene refers to the chloroplastic protein.

| **adj P Val** | **Log_2_FC** | **Leading proteins** | **Protein name** | **Protein name*** | **Gene name*** | **Positions within proteins** | **Localization prob** | **PEP** | **Unique_identifier** | **Score for localization** | **Sequence window** | **Nr of Oxidation M** |
| --- | --- | --- | --- | --- | --- | --- | --- | --- | --- | --- | --- | --- |
| 0.0013 | 6.09 | A0A2N9HBP3 | AP-3 complex subunit beta C-terminal domain-containing |  | At3g55480 | 1024; 102; 213; 264; 545 | 1 | 0.027551 | UID28 | 85.676 | SYLESSKDIWDTLRLMYSSEENITRIHELYQ | 1 |
| 0.000031 | 5.72 | A0A5C7IQF1 | Outer envelope pore protein 16-2, chloroplastic |  | At4g16160 | 1 | 1 | 9.01E-19 | UID616 | 187.16 | _______________MDSSSITSNLEHRSLL | 1 |
| 0.000098 | 5.67 | A0A2N9FGJ5 | Uncharacterized protein | Protein sulfur deficiency-induced 1 | At5g48850 | 268 | 1 | 0.003056 | UID64 | 94.692 | LVGLDDDFVKGLEQLMDEWGPLRSKRLPIFE | 1 |
| 0.000031 | 5.41 | A0A5C7IXN9 | Osmotin-like protein |  | At4g11650 | 241 | 1 | 8.26E-46 | UID671 | 42.185 | ACPATFTYAHDSPTLMHECSSPRELKVIFCH | 1 |
| 0.000082 | 5.13 | A0A5C7GSL4 | BED-type domain-containing protein | Zinc finger BED domain-containing protein RICESLEEPER | At3g56820 | 144 | 1 | 0.007317 | UID176 | 83.692 | YDLARMIILHGYPLTMVDHVGFRVFVKNLQP | 1 |
| 0.000098 | 4.98 | A0A5C7IIR0 | Tr-type G domain-containing protein | Elongation factor 2 | At1g56070 | 759 | 1 | 0.01243 | UID551 | 107.9 | IYSVLNQKRGHVFEEMQRQGTPLYNIKAYLP | 1 |
| 0.000056 | 4.86 | A0A5C7HLQ1 | ATP synthase subunit beta (mitochondrial) |  | At5g08690 | 117 | 1 | 5.4E-08 | UID387 | 67.866 | RFDDGLPPILTALEVMDHSIRVVLEVASHLG | 1 |
| 0.000085 | 4.2 | A0A5C7I6D2 | Peptidylprolyl isomerase |  | At3g25230 | 107 | 1 | 6.15E-32 | UID505 | 182.41 | DENFTKKHTEAGILSMANAGPGTNGSQFFIC | 1 |
| 0.00012 | 4.2 | A0A5C7H9F7 | Peptidase A1 domain-containing protein |  | At3g46910 | 412 | 1 | 1.82E-05 | UID301 | 154.05 | YQLQDNLLQFDLARSMLGFSSSLLLRGTSCS | 1 |
| 0.00014 | 3.99 | A0A2N9I9Y5;A0A5C7ID52;A0A5C7IWK4 | 5-methyltetrahydropteroyltriglutamate--homocysteine S-methyltransferase |  | At3g03780 | 557; 601 | 1 | 1.19E-39 | UID132 | 266.93 | WSSAAQSMTARPMKGMLTGPVTILNWSFVRN; WSSMAQSMTARPMKGMLTGPVTILNWSFVRN; WSSMAQSMTKRPMKGMLTGPVTILNWSFVRN | 1 |
| 0.0082 | 3.79 | A0A5C7H8B7 | Peroxidase | Peroxidase 12 | At1g71695 | 317 | 1 | 3.1E-07 | UID293 | 125.89 | LFFDQFALSMIKMGQMSVLTGTLGEVRANCS | 1;2 |
| 0.00058 | 3.63 | A0A2N9I406 | Signal recognition particle 9 kDa protein |  | At3g49100 | 73 | 1 | 0.019527 | UID126 | 73.573 | DAKKMEKLNNIFFTLMARGPDADVSEVAGKE | 1 |
| 0.02 | 3.44 | A0A5C7H6Y2 | Glycosyltransferase | UDP-glycosyltransferase 73C4 | At2g36770 | 423 | 1 | 0.022088 | UID967 | 60.518 | DDLVKGIEQLMGDHEMKKRAQMLSSKFQQGF |  |
| 0.00041 | 3.41 | A0A2N9F3C7;A0A6B9S1H9;A0A5C7H8Z0;A0A5C7HT26;A0A5C7IX25 | Tubulin alpha chain | Alpha-2.4 tubulin | At1g04820 | 129; 268; 256 | 1 | 8.83E-20 | UID42 | 91.076 | TEFQTNLVPYPRIHFMLSSYAPVISAEKAFH;TEFQTNLVPYPRIHFMLSSYAPVISAEKAYH | 1 |
| 0.0079 | 3.4 | A0A2N9IT34 | Uncharacterized protein | Protein NRT1/ PTR FAMILY 2.6-like | At3g45660 | 1 | 1 | 0.001248 | UID145 | 67.726 | _______________MDGREAQISNSAGSKR | 1 |
| 0.00043 | 3.28 | A0A5C7IIR0 | Tr-type G domain-containing protein | Elongation factor 2 | At1g56070 | 589 | 1 | 5.6E-14 | UID554 | 164.94 | RTVMSKSPNKHNRLYMEARPLEDGLAEAIDD | 1 |
| 0.00015 | 3.25 | A0A5C7HMQ2 | Fructose-bisphosphate aldolase |  | At2g21330 | 136 | 1 | 3.03E-18 | UID401 | 92.198 | NDHHVLLEGTLLKPNMVTPGSESPKVTPEVI | 1 |
| 0.012 | 3.2 | A0A5C7H6Y2 | Glycosyltransferase | UDP-glycosyltransferase 73C3 | At2g36780 | 418 | 1 | 0.022088 | UID966 | 60.518 | QMVNKDDLVKGIEQLMGDHEMKKRAQMLSSK |  |
| 0.00043 | 3.17 | A0A2N9F361 | Transketolase |  | At3g60750 | 241 | 1 | 8.2E-09 | UID40 | 113.65 | NKPDAILVDHRTFCIMGDGCAMEGISHEAAS | 1 |
| 0.00031 | 3.05 | A0A5C7HBX9 | Peptidylprolyl isomerase |  | At3g62030 | 89 | 0.933493 | 3.29E-05 | UID323 | 85.402 | GKGSVIKGWDEGVMGMQLGETARLRCSPDYA | 1 |
| 0.0006 | 3.01 | A0A5C7IIR0;A0A5C7IRX9 | Tr-type G domain-containing protein | Elongation factor 2 | At1g56070 | 377 | 1 | 0.002433 | UID556 | 84.718 | QYANAIRNCDPDGPLMLYVSKMIPASDKGRF | 1 |
| 0.00026 | 2.98 | A0A5C7IX98;A0A6B9S3G2 | Elongation factor 1-alpha |  | At1g07940 | 264 | 1 | 7.05E-10 | UID665 | 104.81 | TVPVGRVETGIIKPGMVVTFGPTGLTTEVKS | 1 |
| 0.0032 | 2.92 | A0A5C7IWE5 | Poly [ADP-ribose] polymerase |  | At2g31320 | 683 | 1 | 1.52E-07 | UID655 | 140.14 | KGFLPAVCSLPVPGYMFGKAIVCSDAVAEAA | 1 |
| 0.002 | 2.78 | A0A5C7GZ87 | T-complex protein 1 subunit beta (CCT-beta) |  | At5g20890 | 119 | 1 | 0.015351 | UID215 | 52.167 | LLREAEKLVAAKIHPMTIISGFRMAAECACN | 1 |
| 0.0021 | 2.74 | A0A5C7HAR9;A0A5C7HN13 | Ribosomal protein L3 |  | At2g43030 | 217; 193; 47 | 1 | 0.00028 | UID310 | 90.968 | FEKQVPVDAVFQKDEMIDIIGVTKGKGYEGV | 1 |
| 0.0026 | 2.71 | A0A5C7HXN9;A0A5C7HYD1;A0A5C7IJ29 | ATP-dependent RNA helicase | Eukaryotic initiation factor 4A | At1g54270 | 194; 266; 207; 300; 193 | 1 | 0.001236 | UID467 | 113.69 | RADHIKMFVLDEADEMLSRGFKDQMYDIFQL;RPDHIKMFVLDEADEMLSRGFKDQIYDIFQL;RPDYIKMFVLDEADEMLSRGFKDQIYDIFQL | 1 |
| 0.0006 | 2.67 | A0A5C7H3Z3 | UDP-arabinopyranose mutase |  | At3g08900 | 29 | 1 | 0.00131 | UID261 | 94.45 | LDIVIPTIRNLDFLEMWRPFFEKYHLIIVQD | 1 |
| 0.00086 | 2.65 | A0A5C7HLY0 | UDP-glucose 6-dehydrogenase |  | At1g26570 | 437 | 1 | 3.71E-05 | UID392 | 103.39 | WDEFKSLDYQRIYEGMQKPAFVFDGRNIVDA | 1 |
| 0.0055 | 2.64 | A0A2N9HHE9;A0A5C7IB13 | T-complex protein 1 subunit zeta |  | At3g02530 | 113; 78 | 1 | 0.02274 | UID116 | 96.492 | IGELMKQSERYIDEGMHPRVLVDGFEIAKRA | 1 |
| 0.024 | 2.56 | A0A2N9FQY6;A0A2N9FY75;A0A5C7HR14;A0A5C7GUZ6;A0A5C7H156;A0A5C7INT9;A0A5C7IP03;A0A6B9S303 | Tubulin beta chain | Tubulin beta-1 chain | At1g75780 | 149; 147 | 1 | 2.43E-29 | UID76 | 156.26 | GFQVCHSLGGGTGSGMGTLLISKIREEYPDR | 1 |
| 0.00031 | 2.55 | A0A2N9HCL4 | Uncharacterized protein | Elongation factor 1-gamma | At1g57720 | 270 | 1 | 0.02952 | UID112 | 102.77 | KPKAKNPLDLLPPSKMILDDWKRLYSNTKSN | 1 |
| 0.0022 | 2.54 | A0A5C7HL76 | 60S ribosomal protein L27 |  | At3g22230 | 81 | 1 | 0.000321 | UID385 | 67.08 | SRVKAFIKLVNYQHLMPTRYTLDVDLKDIVT | 1 |
| 0.00096 | 2.51 | A0A5C7IUE7 | Large ribosomal subunit protein uL18 C-terminal eukaryotes domain-containing protein |  | At5g37760 | 211 | 1 | 2.66E-08 | UID637 | 72.421 | KYIFGGHVAAYMRTLMEDEPEKYQSHFCEFI | 1 |
| 0.039 | 2.49 | A0A5C7HDW7 | 11-beta-hydroxysteroid dehydrogenase-like 4A |  | At5g50590 | 192 | 1 | 2.94E-07 | UID334 | 168.43 | KGNIVALSSADAWLPMPRHSFYNVFN_____ | 1 |
| 0.0021 | 2.47 | A0A5C7IT64 | Malate dehydrogenase |  | At1g04410 | 30 | 0.99999 | 9.47E-06 | UID624 | 82.904 | GQIGYALVPMIARGVMLGADQPVILHMLDIP | 1 |
| 0.0021 | 2.44 | A0A5C7HHI8 | DUF642 domain-containing protein | Protein DUF642 L-galactono-1.4-lactone-responsive gene 2-like | At5g25460 | 78 | 1 | 2.47E-12 | UID362 | 101.59 | KGLVEYIHGGPQPGGMFFAVSHGVHAVRLGN | 1 |
| 0.0093 | 2.38 | A0A5C7GYD0;A0A5C7GZ31 | Phorbol-ester/DAG-type domain-containing protein |  | At2g17370 | 107 | 1 | 0.000152 | UID203 | 100.39 | KHWPFKVIPGPADKPMIVVTYKGEEKQFFAE; KHWPFKVIPGPADKPMIVVTYKGEEKQFSAE | 1 |
| 0.025 | 2.38 | A0A5C7HAY7 | Glutathione transferase | Elongation factor 1-gamma | At1g09640 | 84 | 1 | 0.002131 | UID311 | 77.871 | AEYSGVQVKLVEDFQMGVSNKTPEFLKMNPI | 1 |
| 0.0007 | 2.37 | A0A5C7IHK2;A0A5C7HWI3 | Profilin |  | At2g19760 | 64; 73; 43 | 1 | 0.000192 | UID3 | 100.02 | GSLAPTGLHLAGAKYMVIQGEPGAVIRGKKG; GTLAPTGLYLGGTKYMVIQGEPGAVIRGKKG | 1 |
| 0.00049 | 2.22 | A0A5C7HPV8 | Ribosomal protein S11 |  | At1g79850 | 74; 126 | 1 | 9.5E-05 | UID416 | 91.166 | MKVKADRDESSPYAAMLAAQDVSQRCKELGI | 1 |
| 0.0014 | 2.22 | A0A5C7IFK8 | Water stress and hypersensitive response domain-containing protein |  | At2g41430 | 19 | 1 | 0.005808 | UID538 | 54.259 | LLNKAKEFVADKVVHMKKPEASVEDVDLKNV | 1 |
| 0.0028 | 2.22 | A0A5C7J1B7 | Glycosyltransferase | UDP-glycosyltransferase 87A2 | At2g30140 | 1 | 1 | 9.07E-10 | UID682 | 189.13 | _______________MINNVCHIVAMPYPAK | 1 |
| 0.0037 | 2.19 | A0A5C7IIR0 | Tr-type G domain-containing protein | Elongation factor 2 | At1g56070 | 412 | 1 | 0.001781 | UID552 | 114.97 | RVFAGKVSTGLKVRIMGPNYVPGEKKDLYTK | 1 |
| 0.045 | 2.12 | A0A5C7IUF8 | Uncharacterized protein | Chaperonin CPN60-2. mitochondrial | At3g13470 | 153 | 1 | 1.34E-05 | UID638 | 98.407 | VAAGMNAMDLRRGISMAVDAVVTNLKSRAKM | 1 |
| 0.0049 | 2.1 | A0A5C7H410 | Malate dehydrogenase |  | At1g53240 | 110 | 1 | 7.9E-05 | UID262 | 55.589 | GASGGIGQPLALLVKMSPLVSALHLYDIANV | 1 |
| 0.0031 | 2.08 | A0A5C7H1R9 | Dihydropyrimidine dehydrogenase (NADP(+)) |  | At3g18860 | 365 | 1 | 0.017788 | UID234 | 70.488 | GLVKKLCDELKDFMKMHNFSSIEDFRGASLE | 1 |
| 0.0039 | 2.08 | A0A5C7HLY0 | UDP-glucose 6-dehydrogenase |  | At1g26570 | 318 | 1 | 0.001017 | UID393 | 86.014 | NDYQKSRFVNRVVASMFNTVSSKKIAILGFA | 1 |
| 0.0019 | 2.07 | A0A2N9FIA8 | 40S ribosomal protein S18 |  | At4g09800 | 68 | 1 | 0.002781 | UID67 | 85.554 | NKRAGELTAAELDNLMVIVANPRQFKIPDWF | 1 |
| 0.0061 | 2.02 | A0A2N9H628 | Polyadenylate-binding protein | Polyadenylate-binding protein 2-like | At1g11650 | 374 | 1 | 0.027601 | UID108 | 64.65 | TSEEASRALDSMNGKMVVSKPLYVALAQRKE | 1 |
| 0.024 | 2.02 | A0A5C7HF14 | 14-3-3 domain-containing protein | 14-3-3-like protein D | At5g10450 | 152 | 1 | 0.012794 | UID350 | 91.076 | EFKSGDEKKEVAANSMKAYETATSTAEAELA | 1 |
| 0.0035 | 2.01 | A0A2N9GJN6 | Integrase catalytic domain-containing protein | Gag-Pol polyprotein | At2g38695 | 13 | 1 | 0.034916 | UID1479 | 53.754 | ___MPFGLSNAPSTFMRVMTQVLRPFMGKFV | 3 |
| 0.019 | 2 | A0A5C7H8B7 | Peroxidase | Peroxidase 12 | At1g71695 | 317 | 1 | 3.1E-07 | UID981 | 125.89 | LFFDQFALSMIKMGQMSVLTGTLGEVRANCS | 1;2 |

| **adj P Val** | **Log_2_FC** | **Proteins** | **Protein name** | | **Explicated protein name**** | **Gene*** | **Positions within proteins** | **Localization prob** | **PEP** | **Unique_identifier** | **Score for localization** | **Sequence window** | **Nr of Oxidation M** |
| --- | --- | --- | --- | --- | --- | --- | --- | --- | --- | --- | --- | --- | --- |
| 0.0012 | -2.01 | A0A5C7ITA3 | SMP domain-containing protein | | Late embryogenesis abundant protein D-34 | At4g26080 | 19 | 1 | 1.95E-17 | UID1314 | 43.696 | EQQKRRQKNEDQQHMMSHEGHQQQQQREPIK | 1; 2 |
| 0.0056 | -2.01 | A0A2N9J1I4; A0A5C7H8B7; A0A5C7H8D0; A0A5C7H8G1 | Peroxidase | | Peroxidase 4 | At1g14540 | 248; 270; 271 | 1 | 0.010196 | UID154 | 128.86 | IRSPNKFDNKYYVDLMNRQGLFTSDQDLYTD; IRTPNLFDNKYYVDLMNRQGLFTSDQDLYTD; IRTPNVFDNKYYVDLMNRQGLFTSDQDLYTD | 1 |
| 0.0012 | -2.03 | A0A5C7GTJ2 | 3-oxoacyl-[acyl-carrier-protein] synthase |  | | At2g04540 | 328 | 1 | 1.69E-05 | UID184 | 56.972 | IAEYLGGAVNCDAYHMTDPRADGLGVSSCIE | 1 |
| 0.0016 | -2.03 | A0A5C7GZ64 | Ferritin | |  | At5g01600 | 170 | 0.999995 | 8.04E-10 | UID214 | 139.48 | YQNKRGGKVKLQSMLMPLTEFDHVEKGDALY | 1 |
| 0.0023 | -2.03 | A0A5C7HF87 | ADP/ATP translocase | |  | At3g08580 | 325; 262 | 1 | 0.000253 | UID351 | 120.99 | RMMMTSGEAVKYKSSMDAFSQIVKNEGAKSL | 1 |
| 0.0037 | -2.03 | A0A5C7IMQ9 | NADH-ubiquinone oxidoreductase 21kDa subunit N-terminal domain-containing protein |  | | At4g19310 | 1 | 1 | 1.5E-07 | UID590 | 122.34 | _______________MNTDITASEKPQYPVI | 1 |
| 0.0012 | -2.04 | A0A5C7ITA3 | SMP domain-containing protein | | Late embryogenesis abundant protein D-34 | At4g26080 | 18 | 1 | 2.83E-09 | UID1313 | 43.696 | QEQQKRRQKNEDQQHMMSHEGHQQQQQREPI | 1; 2 |
| 0.0026 | -2.06 | A0A5C7H2Y8 | MI domain-containing protein | | MA3 domain-containing translation regulatory factor 1 | At5g63190 | 15 | 1 | 3.23E-05 | UID243 | 110.8 | _MASSEGFLTEEQREMFKLATQNAEILSSSP | 1 |
| 0.00074 | -2.08 | A0A5C7HKG3 | Uncharacterized protein | | Protein fatty acid export 3 | At2g38550 | 124 | 1 | 0.001864 | UID1068 | 115.14 | TWKQALASFKEQALKMQSVSQEAYEMYSKKA | 2 |
| 0.00091 | -2.08 | A0A5C7INE0 | Major facilitator superfamily (MFS) profile domain-containing protein |  | | At3g21250 | 282 | 1 | 1.97E-11 | UID1283 | 133.1 | MFGVSAIPSILLALGMSFSPESPRWLFQQGK | 2 |
| 0.00091 | -2.1 | A0A5C7HXY9 | Sucrose synthase | |  | At1g73370 | 528; 516; 77; 369 | 1 | 7.62E-05 | UID468 | 91.845 | DVFDPKFNIVSPGADMSIYFPYTEEKRRLKS | 1 |
| 0.0045 | -2.1 | A0A5C7HDL0 | Oleosin | | Oleosin 5 | At3g01570 | 138 | 1 | 7E-06 | UID330 | 72.096 | ATGTVPEMADQAKRRMADMAGYVGQKTKDVG | 1; 2 |
| 0.00084 | -2.13 | A0A5C7H415 | Clp R domain-containing protein | | Chaperone protein ClpB1 | At1g74310 | 295 | 1 | 0.000116 | UID263 | 98.694 | DEIHLVLGAGRTEGSMDAANLFKPMLARGQL | 1 |
| 0.00059 | -2.15 | A0A5C7HX23 | Ubiquinone biosynthesis protein | |  | At2g30920 | 216 | 1 | 4.59E-07 | UID459 | 88.836 | QAQPLNVSTSFKQRAMLVDEIWHAAGDEGSD | 1 |
| 0.001 | -2.15 | A0A5C7GZ87 | T-complex protein 1 subunit beta (CCT-beta) |  | | At5g20890 | 482 | 1 | 1.73E-05 | UID216 | 130.1 | KVKQAVLLSATEASEMILRVDEIITCAPRKR | 1 |
| 0.011 | -2.15 | A0A2N9EW44; A0A2N9J2E3; A0A5C7IFK6 | Histidine kinase/HSP90-like ATPase domain-containing protein |  | | At5g56030 | 589; 581; 584 | 1 | 6.94E-09 | UID33 | 104.55 | SPCCLVTGEYGWTANMERIMKAQALRDNSMA; SPCCLVTGEYGWTANMERIMKAQALRDSSMA | 1 |
| 0.022 | -2.17 | A0A5C7IXI8 | Cupin type-1 domain-containing protein | | Vicilin-like seed storage protein At2g28490 | At2g28490 | 113 | 1 | 1.28E-05 | UID670 | 102.87 | GFITMEPKTLFIPQYMDSSLILFVRTGEARV | 1 |
| 0.00048 | -2.19 | A0A5C7GZL1 | Oleosin | |  | At4g25140 | 28 | 1 | 0.000285 | UID218 | 106.68 | LQHQPQNYPTDAFRGMLPDHSPSKSQIIAVV | 1 |
| 0.00044 | -2.21 | A0A5C7HYU8 | SHSP domain-containing protein | | 17.3 kDa class II heat shock protein-like | At4g14830 | 19 | 1 | 1.85E-12 | UID471 | 116.39 | RMIEVDSPLFSTLRHMIDTSEDIDKSYNAPT | 1 |
| 0.0012 | -2.21 | A0A5C7H8I5 | Variable large protein | | D-3-phosphoglycerate dehydrogenase | At4g34200 | 1 | 1 | 1.65E-05 | UID298 | 95.631 | _______________MNSTEGTEEKNEQGDV | 1 |
| 0.0044 | -2.26 | A0A5C7IJX8 | Phytocyanin domain-containing protein | | Stellacyanin | At5g20230 | 4 | 1 | 0.035579 | UID1948 | 49.929 | ____________MEKMLVVLVVVMAAISCLG | 3 |
| 0.00097 | -2.29 | A0A5C7H3U3 | Eukaryotic peptide chain release factor GTP-binding subunit |  | | At1g18070 | 279 | 1 | 0.01367 | UID948 | 65.598 | KDVQFLPLSGLMGTNMQTRVNKNTCPWWNGP | 2 |
| 0.00077 | -2.33 | A0A5C7ITA3 | SMP domain-containing protein | | Late embryogenesis abundant protein D-34 | At4g26080 | 19 | 1 | 1.95E-17 | UID626 | 43.696 | EQQKRRQKNEDQQHMMSHEGHQQQQQREPIK | 1;2 |
| 0.0006 | -2.36 | A0A5C7GXQ0 | Uncharacterized protein | | Mitochondrial dicarboxylate/tricarboxylate transporter DTC | At5g19760 | 582; 417 | 1 | 0.000523 | UID197 | 90.108 | DAQGKYPYTGSLDCAMKTLKAGGPFKFYTGF | 1 |
| 0.00091 | -2.36 | A0A2N9E196; A0A5C7HNF2; A0A5C7IY72 | Actin | | Actin-1 | At5g59890 | 267; 268 | 0.995427 | 2.2E-08 | UID5 | 100.95 | GAERFRCPEVLFQPSMIGMEAAGIHETTYNS | 1 |
| 0.0005 | -2.39 | A0A5C7IJX8 | Phytocyanin domain-containing protein | | Stellacyanin | At5g20230 | 12 | 1 | 0.035579 | UID1949 | 49.929 | ____MEKMLVVLVVVMAAISCLGGKLAHAQL | 3 |
| 0.00048 | -2.4 | A0A5C7IEE3 | DJ-1/PfpI domain-containing protein | | Protein DJ-1 homolog B | At1g53280 | 385 | 1 | 0.030599 | UID533 | 98.04 | LYGKEKADEVAGPLVMRSNHGDEYTITEFSP | 1 |
| 0.00073 | -2.4 | A0A2N9J0P3 | RRM domain-containing protein | | Serine/arginine-rich-splicing factor SR34-like | At1g02840 | 145 | 1 | 1.06E-06 | UID153 | 90.926 | GSGTTGIVDYANYEDMKYAVKKLDDSEFRNA | 1 |
| 0.0016 | -2.4 | A0A5C7IJX8 | Phytocyanin domain-containing protein | | Stellacyanin | At5g20230 | 1 | 1 | 0.035579 | UID1947 | 49.929 | _______________MEKMLVVLVVVMAAIS | 3 |
| 0.0044 | -2.42 | A0A5C7GYN4 | ATP synthase subunit d, mitochondrial |  | | At3g52300 | 27 | 1 | 0.026209 | UID204 | 57.59 | AFKAGRTIDWDGMAKMLVSEEARKEFSALRR | 1 |
| 0.00045 | -2.43 | A0A5C7HFK5 | ADP/ATP translocase | | ADP, ATP carrier protein, mitochondrial | At3g08580 | 95 | 1 | 0.008814 | UID352 | 76.156 | APSEKGAAGFAIDFLMGGVSAAVSKTAAAPI | 1 |
| 0.03 | -2.44 | A0A5C7I4P4 | D-3-phosphoglycerate dehydrogenase |  | | At3g19480 | 369 | 1 | 1.58E-28 | UID496 | 142.22 | GALKGELAATAVNAPMVPAEVLTELKPYVVL | 1 |
| 0.0012 | -2.45 | A0A2N9FXD0 | Reverse transcriptase domain-containing protein | | LINE-1 reverse transcriptase-like | At1g65750 | 1 | 0.992815 | 0.018021 | UID83 | 67.563 | _______________MMTEFAFSVAEKVIEK | 1 |
| 0.0083 | -2.45 | A0A5C7H8B7; A0A5C7H8G1 | Peroxidase | | Peroxidase 12 | At1g71695 | 231;230 | 1 | 0.000613 | UID291 | 73.11 | CSSFTDRLYPTQDPTMDKTFANNLKKTCPAS; CSSFTDRLYPTQDPTMDKTFANNLKKTCPAV | 1 |
| 0.00073 | -2.48 | A0A5C7IK54 | Uncharacterized protein | | Late embryogenesis abundant protein 2 | At1g02820 | 71 | 1 | 5.49E-08 | UID574 | 121.02 | AAKDKTVQAAKAAQDMATQAAQATKDKAGQT | 1 |
| 0.00059 | -2.52 | A0A5C7HBQ1 | 14-3-3 domain-containing protein | | 14-3-3 protein 6 | At1g22300 | 265 | 1 | 1.48E-14 | UID319 | 141.64 | IMQLLRDNLTLWTSDMQDDGTDDIKEAPKAE | 1 |
| 0.011 | -2.54 | A0A5C7IN17 | Small ribosomal subunit protein uS10 |  | | At3g13120 | 7 | 1 | 0.020142 | UID591 | 80.377 | _________MAAYVAMKGKPGLEEPQEQIHK | 1 |
| 0.00031 | -2.55 | A0A5C7IWC8 | Ribosomal protein | | 60S ribosomal protein L10a-3 | At5g22440 | 17 | 1 | 0.027776 | UID652 | 83.869 | SKLQSDALREAISAIMLYSKETKKRNFTETV | 1 |
| 0.0006 | -2.56 | A0A5C7HQL8 | Large ribosomal subunit protein uL4 C-terminal domain-containing protein |  | | At5g02040 | 384 | 1 | 1.37E-31 | UID421 | 174.47 | EATAIKAAGKAWYQTMISDSDYTEFENFSKW | 1 |
| 0.00032 | -2.57 | A0A5C7H3U3 | Eukaryotic peptide chain release factor GTP-binding subunit |  | | At1g18070 | 275 | 1 | 0.01367 | UID947 | 65.598 | YNVKKDVQFLPLSGLMGTNMQTRVNKNTCPW | 2 |
| 0.00036 | -2.57 | A0A2N9FT44 | Histone H2A | |  | At4g27230 | 110 | 1 | 0.003744 | UID81 | 59.975 | LSKLLGDVTIANGGVMPNIHNLLLPKKAGSS | 1 |
| 0.00097 | -2.58 | A0A5C7HQT7 | Cysteine proteinase | | Cysteine proteinase RD21 | At1g47128 | 237 | 1 | 0.00833 | UID424 | 80.752 | VVSIDGYEDVSPFDEMSLKKAVSHQPVSVAI | 1 |
| 0.0002 | -2.67 | A0A5C7HEV3 | Beta-amylase | |  | At3g23920 | 176 | 1 | 6.46E-05 | UID347 | 69.704 | LFEVIQQCGLKLQAIMSFHQCGGNVGDVVTI | 1 |
| 0.00055 | -2.67 | A0A5C7ILL1; A0A5C7IMA8 | Pentacotripeptide-repeat region of PRORP domain-containing protein |  | | At1g60070 | 266; 291 | 1 | 0.004007 | UID584 | 95.573 | FKGEVIYRTLLANCVMAVNVKKAEEVFNKMK; FQGEVIYRTLLANCVMAVNVKKAEEVFNKMK | 1 |
| 0.00055 | -2.67 | A0A2N9EX04 | CCHC-type domain-containing protein | | Serine/threonine-protein phosphatase 7 long form | At1g48120 | 297 | 1 | 0.00562 | UID34 | 60.161 | TQAQLTSGLEFSTHAMDKFEKWSEKASLHHV | 1 |
| 0.031 | -2.69 | A0A5C7IDQ0 | UspA domain-containing protein | |  | At3g01520 | 224 | 1 | 0.000249 | UID529 | 113.72 | DKDGGEPLVKIKQHDMEVDHQDEKLKG____ | 1 |
| 0.0004 | -2.7 | A0A5C7HAJ8 | DUF1985 domain-containing protein |  | | At3g32960 | 200 | 1 | 0.03493 | UID308 | 78.655 | LKVNVHNKKIEGIGLMEDLLNRDMSLYNDNL | 1 |
| 0.00059 | -2.71 | A0A5C7HM46 | UspA domain-containing protein | |  | At4g27320 | 1 | 1 | 9.75E-43 | UID396 | 249.2 | _______________MEETTDASAAMQQQPL | 1 |
| 0.00047 | -2.77 | A0A2N9IL82; A0A5C7HES7 | T-complex protein 1 subunit gamma |  | | At5g26360 | 579; 506 | 1 | 0.001062 | UID138 | 111.61 | NVKAQTFKTAIEAACMLLRIDDIVSGIKKKQ | 1 |
| 0.00077 | -2.82 | A0A5C7H9X2 | AAA+ ATPase domain-containing protein | | Protein STICHEL | At2g02480 | 1400 | 1 | 4.05E-10 | UID304 | 123.43 | GQQQAGQTGTHQQSQMVDRAQTVVTYCRLPQ | 1 |
| 0.00073 | -2.85 | A0A5C7H3K9 | Glyceraldehyde-3-phosphate dehydrogenase |  | | At3g04120 | 329 | 1 | 0.000957 | UID256 | 56.013 | NEWGYSSRVVDLIVHMAKSQ___________ | 1 |
| 0.00051 | -2.87 | A0A5C7HB18 | Uncharacterized protein | | Heat shock 70 kDa protein. mitochondrial | At4g37910 | 651 | 1 | 5.27E-12 | UID314 | 82.702 | KLDVANKAVSKIGQHMAGGSGGGSASGGSQG | 1 |
| 0.00034 | -2.9 | A0A499QZC2; A0A7G7XUL0 | ATP synthase subunit beta, chloroplastic |  | | AtCg00480 | 86; 47 | 0.999978 | 0.008252 | UID1 | 81.338 | NNRVRAVAMSATDGLMRGIEVIDTGAPLSVP | 1 |
| 0.00098 | -2.93 | A0A2N9HL54;A0A5C7GTN4 | Chlorophyll a-b binding protein, chloroplastic |  | | At5g54270 | 108; 105; 102; 30; 110 | 1 | 1.21E-06 | UID120 | 156.01 | FAKNRELEVIHSRWAMLGALGCVFPELLARN | 1 |
| 0.00031 | -3 | A0A2N9EV21;A0A2N9FRJ0; A0A5C7IX98; A0A5C7IYI4; A0A6B9S3G2 | Elongation factor 1-alpha | |  | At1g07920 | 235; 282 | 1 | 0.002266 | UID22 | 58.366 | TFGPTGLTTEVKSVEMHHEALLEALPGDNVG | 1 |
| 0.00026 | -3.01 | A0A5C7HUH5 | Cytosol aminopeptidase domain-containing protein | | Leucine aminopeptidase 1-like | At2g24200 | 115 | 1 | 1.34E-20 | UID443 | 122.68 | MAKDENSKFQNSILKMLDAQLGGLLSEASSE | 1 |
| 0.0085 | -3.03 | A0A5C7IFM5 | SUI1 domain-containing protein | | Protein translation factor sui1 homolog 2 | At1g54290 | 135 | 1 | 6.25E-38 | UID540 | 97.741 | IEIAESIPSMCNMRPMECDFESSRSDMSRQW | 1; 2 |
| 0.00014 | -3.08 | A0A5C7IQ10 | Succinate-semialdehyde dehydrogenase, mitochondrial |  | | At1g79440 | 452 | 1 | 0.001536 | UID610 | 51.76 | KGAKVLLGGKRHSLGMTFYEPTVIGDVKNDM | 1 |
| 0.00035 | -3.08 | A0A5C7GPW9 | DUF4005 domain-containing protein | | Regulator of rDNA transcription protein 15 | At3g44530 | 54 | 1 | 0.03148 | UID1539 | 66.27 | GHDCPLNTLERSKSEMYQMLRMRPTLHGRGQ | 3 |
| 0.0011 | -3.08 | A0A5C7HRJ0 | Uncharacterized protein | | Late embryogenesis abundant protein LEA_5 subgroup | At3g51810 | 208 | 1 | 0.010617 | UID428 | 114.97 | HEGYQEMGRKGGLSTMEKSGAQRVAEEGIDI | 1 |
| 0.015 | -3.09 | A0A5C7HDY5 | Phospholipase A1 | |  | At4g18550 | 534 | 1 | 1.34E-24 | UID336 | 122.79 | TIQQTLGGVTEQGNQMAKHQGGDNRGGQQSL | 1 |
| 0.00017 | -3.1 | A0A5C7IJK9 | Ig-like domain-containing protein | | Heat shock 70 kDa protein 14 | At1g79930 | 57 | 1 | 0.000059 | UID1254 | 98.156 | GDKQRFIGTAGAATSMMNPKNTISQIKRLIG | 1; 2 |
| 0.00026 | -3.11 | A0A5C7H4P3 | Hyaluronan/mRNA-binding protein domain-containing protein |  | | At4g20580 | 272 | 1 | 0.001026 | UID266 | 64.04 | RKDAADKEEKARKSVMISEFLKPAEGERYYG | 1 |
| 0.0011 | -3.15 | A0A5C7IJK9 | Ig-like domain-containing protein | | Heat shock 70 kDa protein 14 | At1g79930 | 58 | 1 | 1.45E-05 | UID1255 | 98.156 | DKQRFIGTAGAATSMMNPKNTISQIKRLIGR | 1; 2 |
| 0.00021 | -3.17 | A0A5C7HNF5 | Monodehydroascorbate reductase (NADH) |  | | At1g63940 | 321 | 1 | 0.003677 | UID409 | 76.588 | SVPDVYAVGDVATFPMKLYNEIRRVEHVDHA | 1 |
| 0.00015 | -3.2 | A0A5C7IQ04 | Peroxiredoxin | | 1-Cys peroxiredoxin | At1g48130 | 211 | 1 | 0.000129 | UID607 | 90.653 | EMFPKGYQTVDLPSKMDYLRFTNVV______ | 1 |
| 0.00015 | -3.2 | A0A5C7I2L5 | Glutathione transferase | | Glutathione S-transferase F6 | At1g02930 | 123 | 1 | 1.02E-24 | UID486 | 87.339 | DPVASKLNWEIVFKPMFGMTTDPAAVEELEA | 1; 2 |
| 0.0004 | -3.21 | A0A5C7GPW9 | DUF4005 domain-containing protein | | Regulator of rDNA transcription protein 15 | At3g44530 | 60 | 1 | 0.03148 | UID1541 | 66.27 | NTLERSKSEMYQMLRMRPTLHGRGQNQHAEA | 3 |
| 0.00014 | -3.24 | A0A5C7HM75 | Uncharacterized protein | | Seed maturation protein LEA 4 | At3g12960 | 79 | 1 | 1.35E-05 | UID397 | 136.57 | LNQEQAHLHNEEAKRMAQSGAHPGYKTRGTT | 1 |
| 0.00073 | -3.38 | A0A5C7H2H1 | Endoplasmic reticulum transmembrane protein |  | | At1g07810 | 68 | 1 | 1.91E-05 | UID237 | 162.36 | GFQLLDIYWKNEHRLMCTSETCTATERDRYE | 1 |
| 0.00041 | -3.39 | A0A5C7H8G1 | Peroxidase | | Peroxidase 4 | At1g14540 | 313 | 1 | 4.43E-35 | UID294 | 164.81 | DEKLFFDQFALSMIKMGQMNVLTGTLGEVRA | 1; 2 |
| 0.00016 | -3.44 | A0A5C7H8G1 | Peroxidase | | Peroxidase 4 | At1g14540 | 316 | 1 | 8.26E-09 | UID983 | 164.81 | LFFDQFALSMIKMGQMNVLTGTLGEVRANCS | 1; 2 |
| 0.00026 | -3.44 | A0A5C7HIR9 | RRM domain-containing protein | | Nucleolin 2-like | At3g18610 | 379 | 1 | 7.23E-09 | UID372 | 112.56 | TPSTPQAATGSKTLFMGNLPFQIEQSDVEHF | 1 |
| 0.00014 | -3.51 | A0A5C7H720 | 14-3-3 domain-containing protein | |  | At1g78300 | 245 | 1 | 8.91E-15 | UID280 | 48.958 | IMQLLRDNLTLWTSDMQDDGADEIKEAAPKA | 1 |
| 0.0002 | -3.61 | A0A5C7H8G1 | Peroxidase | | Peroxidase 4 | At1g14540 | 313 | 1 | 4.43E-35 | UID982 | 164.81 | DEKLFFDQFALSMIKMGQMNVLTGTLGEVRA | 1; 2 |
| 0.012 | -3.62 | A0A2N9E196; A0A2N9EGA0; A0A2S1KMY5; A0A5C7HAQ0; A0A5C7HNF2; A0A5C7IY72 | Actin | | Actin-1 | At5g59890 | 45;46 | 1 | 9.88E-12 | UID696 | 132.84 | AVFPSIVGRPRHTGVMVGMGQKDAYVGDEAQ | 1; 2 |
| 0.0002 | -3.63 | A0A5C7GRT8 | Uncharacterized protein | | Late embryogenesis abundant protein 14-like | At5g17165 | 242 | 1 | 0.007769 | UID174 | 113.69 | DKDDREKKMDGDVEEMRRRAGERDEKKNNKY | 1 |
| 0.00043 | -3.7 | A0A5C7GPW9 | DUF4005 domain-containing protein | | Regulator of rDNA transcription protein 15 | At3g44530 | 57 | 1 | 0.03148 | UID1540 | 66.27 | CPLNTLERSKSEMYQMLRMRPTLHGRGQNQH | 3 |
| 0.00034 | -3.71 | A0A2N9ECJ3 | SHSP domain-containing protein | | 17.8 kDa class I heat shock protein-like | At1g07400 | 132 | 1 | 0.000911 | UID14 | 82.85 | FRLPDNAKIDEVKACMENGVLTVTVPKEEEK | 1 |
| 0.017 | -3.81 | A0A2N9E196; A0A2N9EGA0; A0A2S1KMY5; A0A5C7HAQ0; A0A5C7HNF2; A0A5C7IY72 | Actin | | Actin-1 | At5g59890 | 48; 49 | 1 | 3.89E-12 | UID697 | 132.84 | PSIVGRPRHTGVMVGMGQKDAYVGDEAQSKR | 1; 2 |
| 0.000098 | -3.85 | A0A2N9IVB5 | RRM domain-containing protein | | U1 small nuclear ribonucleoprotein 70 kDa | At3g50670 | 74 | 1 | 0.017671 | UID147 | 83 | FKPPPEKRKCPPLTGMAQFVTKFAEPGDSEY | 1 |
| 0.024 | -3.95 | A0A5C7H346 | Uncharacterized protein | | Guanine nucleotide-binding protein subunit beta-like protein | At4g34460 | 115 | 1 | 5.43E-08 | UID251 | 163.38 | RFTGHTKDVLSVAFSMDNRQIVSASRDRTIK | 1 |
| 0.0048 | -3.98 | A0A2N9J6T8; A0A5C7IE15 | Peptidase A1 domain-containing protein | | Aspartic proteinase A1 | At1g11910 | 86 | 1 | 2.57E-68 | UID158 | 238.03 | LGGSGDADIVELKNYMDAQYFGEIGIGTPPQ; SGDPEDIDIVSLKNYMDAQYFGEIGIGTPPQ | 1 |
| 0.00004 | -4.03 | A0A5C7HMW5 | Uncharacterized protein | | Late embryogenesis abundant protein At3g53040-like | At3g53040 | 197 | 1 | 0.006137 | UID403 | 136.99 | LGELKDSATGAAKKAMGFLTGKTEEAKYKTA | 1 |
| 0.00018 | -4.08 | A0A5C7HPI8 | Gibberellin-regulated protein 14 |  | | At5g14920 | 240 | 1 | 4.41E-11 | UID415 | 188.99 | GTYGNREKCGKCYTEMTTHGNKPKCP_____ | 1 |
| 0.00014 | -4.09 | A0A5C7GZU4 | HIG1 domain-containing protein | | Hypoxia induced gene | At3g48030 | 1 | 1 | 1.06E-06 | UID222 | 115.82 | _______________MEAVQSWVLEHKLTSI | 1 |
| 0.00048 | -4.2 | A0A5C7HRW5 | Dehydrin | | Dehydrin Xero | At3g50970 | 21 | 1 | 1.21E-14 | UID430 | 159.72 | NQPGATNPQFDEHGNMIRQTDEAGFGSGLGT | 1 |
| 0.00015 | -4.36 | A0A2N9F507 | Reverse transcriptase domain-containing protein | | Retrotransposon. unclassified-like protein | At4g02960 | 587 | 1 | 0.006132 | UID45 | 92.19 | ISAFLSASERTQLVAMLKEYQDVFAWQYNEM | 1 |
| 0.00021 | -4.42 | A0A5C7IWA8 | Uncharacterized protein | | Enoyl-[acyl-carrier-protein] reductase [NADH], chloroplastic | At2g05990 | 219 | 1 | 1.82E-12 | UID648 | 118.43 | SIDILVHSLANGPEVMKPLLETSRKGYLAAL | 1 |
| 0.000098 | -4.46 | A0A5C7HHV4 | DUF4283 domain-containing protein |  | | At1g36960 | 1 | 1 | 0.014137 | UID364 | 67.981 | _______________MNAEDIERLCGYLSLE | 1 |
| 0.00004 | -4.54 | A0A5C7IQF1 | Outer envelope pore protein 16-2, chloroplastic |  | | At4g16160 | 99 | 1 | 0.029494 | UID615 | 86.866 | FPYFKGETNRKSLEAMVKNTSKESLRWGLAA | 1 |
| 0.000098 | -4.55 | A0A5C7HLQ9 | Uncharacterized protein | | Late embryogenesis abundant protein 46 | At5g06760 | 19 | 1 | 2.53E-06 | UID391 | 136.3 | MKETAANIAASATAGMEKTKATIQEKMEKIT | 1 |
| 0.00004 | -4.58 | A0A5C7GRT8 | Uncharacterized protein | | Late embryogenesis abundant protein 14-like | At5g17165 | 235 | 1 | 0.00291 | UID173 | 120.22 | ETVVGKDDKDDREKKMDGDVEEMRRRAGERD | 1 |
| 0.00015 | -4.58 | A0A5C7IFM5 | SUI1 domain-containing protein | | Protein translation factor sui1 homolog 2 | At1g54290 | 132 | 0.949126 | 2.02E-37 | UID1230 | 99.711 | RQIIEIAESIPSMCNMRPMECDFESSRSDMS | 1; 2 |
| 0.00012 | -4.62 | A0A5C7GZ64 | Ferritin | |  | At5g01600 | 85 | 1 | 0.000718 | UID213 | 108.98 | APFEEVKKELDLVPTMPQVSLARQKYSEESE | 1 |
| 0.000082 | -4.86 | A0A5C7GZL1 | Oleosin | |  | At4g25140 | 128 | 1 | 5.09E-07 | UID219 | 91.701 | ANFLRKVGDTTAPEQMQQFKRRIQDTAGQIG | 1 |
| 0.000098 | -5.09 | A0A5C7IFM5 | SUI1 domain-containing protein | | Protein translation factor sui1 homolog 2 | At1g54290 | 135 | 1 | 6.25E-38 | UID1228 | 97.741 | IEIAESIPSMCNMRPMECDFESSRSDMSRQW | 1; 2 |
| 0.000031 | -5.15 | A0A5C7IFM5 | SUI1 domain-containing protein | | Protein translation factor sui1 homolog 2 | At1g54290 | 129 | 0.996085 | 3.9E-45 | UID1229 | 213.2 | QQKRQIIEIAESIPSMCNMRPMECDFESSRS | 1; 2 |
| 0.00004 | -5.27 | A0A5C7I2L5 | Glutathione transferase | | Glutathione S-transferase F6 | At1g02930 | 123 | 1 | 1.02E-24 | UID1174 | 87.339 | DPVASKLNWEIVFKPMFGMTTDPAAVEELEA | 1; 2 |
| 0.0024 | -5.39 | A0A5C7HSM7 | KOW domain-containing protein | | 60S ribosomal protein L26-1 | At3g49910 | 243 | 1 | 1.17E-05 | UID433 | 96.143 | RKAHFTAPSSVRRILMSAPLSTDLRQKYNVR | 1 |
| 0.000033 | -5.76 | A0A2N9H2A0 | Uncharacterized protein | | Ribonuclease H | At5g26940 | 827; 788; 646; 1008 | 1 | 0.017138 | UID94 | 110.87 | MPFGLKNTGATYQRLMNKIVHGLLERNRGQP | 1 |
| 0.000082 | -5.79 | A0A5C7I2L5 | Glutathione transferase | | Glutathione S-transferase F6 | At1g02930 | 126 | 1 | 4.38E-32 | UID1175 | 87.339 | ASKLNWEIVFKPMFGMTTDPAAVEELEAKLS | 1; 2 |
| 0.00014 | -5.83 | A0A5C7IFM5 | SUI1 domain-containing protein | | Protein translation factor sui1 homolog 2 | At1g54290 | 129 | 0.996085 | 3.9E-45 | UID541 | 213.2 | QQKRQIIEIAESIPSMCNMRPMECDFESSRS | 1; 2 |
| 0.00004 | -6.54 | A0A2N9EXT3 | Phosphoacetylglucosamine mutase |  | | At5g18070 | 1 | 0.825115 | 0.028978 | UID35 | 52.391 | _______________MRICQMFNPEQELDSA | 1 |
| 0.000031 | -7.11 | A0A5C7H8G1 | Peroxidase | | Peroxidase 4 | At1g14540 | 316 | 1 | 8.26E-09 | UID295 | 164.81 | LFFDQFALSMIKMGQMNVLTGTLGEVRANCS | 1; 2 |
| 0.000031 | -8.28 | A0A5C7HDL0 | Oleosin | | Oleosin 5 | At3g01570 | 130 | 1 | 0.000314 | UID332 | 108.24 | WVLNSFRQATGTVPEMADQAKRRMADMAGYV | 1 |
