# Supplementary material for: Proteomics- and metabolomics-based analysis of the regulation of germination in Norway maple and sycamore embryonic axes
Source: Tree Physiol. 2025 Jan 6;45(2):tpaf003. doi: 10.1093/treephys/tpaf003 (PMC11791354; doi:10.1093/treephys/tpaf003)
Supplement: Table_S14_tpaf003 [file table_s14_tpaf003.docx]

Table S14. Functional analysis of proteins containing MetO significantly regulated in sycamore embryonic axes with protruded radicles at the germinated stage compared to Norway maple and in terms of molecular function, biological process, cellular compartment and protein class based on Gene Ontology annotation (PANTHER). Child categories are presented for each category. GO terms and numbers (given in brackets) according to PANTHER classification system are followed the number of genes in each category.

MetO sites ad Germinated stage

|  | **Upregulated in sycamore** | **Downregulated in sycamore** |
| --- | --- | --- |
| **MOLECULAR FUNCTION** | **catalytic activity (GO:0003824) 23**  hydrolase activity (GO:0016787) 11  oxidoreductase activity (GO:0016491) 5  catalytic activity, acting on a protein (GO:0140096) 4  transferase activity (GO:0016740) 4  catalytic activity, acting on a nucleic acid (GO:0140640) 2  isomerase activity (GO:0016853) 1  ligase activity (GO:0016874) 1  **binding (GO:0005488) 20**  organic cyclic compound binding (GO:0097159) 12  protein binding (GO:0005515) 5  protein-containing complex binding (GO:0044877) 3  small molecule binding (GO:0036094) 3  amide binding (GO:0033218) 1  carbohydrate derivative binding (GO:0097367) 1  **transporter activity (GO:0005215) 6**  transmembrane transporter activity (GO:0022857) 6  **translation regulator activity (GO:0045182) 5**  translation regulator activity, nucleic acid binding (GO:0090079) 5  **ATP-dependent activity (GO:0140657) 4**  ATPase-coupled transmembrane transporter activity (GO:0042626) 2  ATP hydrolysis activity (GO:0016887) 1  ATP-dependent activity, acting on DNA (GO:0008094) 1  **molecular adaptor activity (GO:0060090) 2**  protein-macromolecule adaptor activity (GO:0030674) 2  **structural molecule activity (GO:0005198) 2**  structural constituent of ribosome (GO:0003735) 2  **antioxidant activity (GO:0016209) 1**  no hit | **catalytic activity (GO:0003824) 21**  oxidoreductase activity (GO:0016491) 6  hydrolase activity (GO:0016787) 5  transferase activity (GO:0016740) 5  catalytic activity, acting on a protein (GO:0140096) 3  isomerase activity (GO:0016853) 3  ligase activity (GO:0016874) 1  lyase activity (GO:0016829) 1  **binding (GO:0005488) 15**  organic cyclic compound binding (GO:0097159) 10  protein binding (GO:0005515) 4  carbohydrate derivative binding (GO:0097367) 3  small molecule binding (GO:0036094) 3  amide binding (GO:0033218) 1  protein-containing complex binding (GO:0044877) 1  **structural molecule activity (GO:0005198) 6**  structural constituent of cytoskeleton (GO:0005200) 3  structural constituent of ribosome (GO:0003735) 3  **translation regulator activity (GO:0045182) 6**  translation regulator activity, nucleic acid binding (GO:0090079) 6  **transporter activity (GO:0005215) 3**  transmembrane transporter activity (GO:0022857) 3  **antioxidant activity (GO:0016209) 1**  no hit |
| **BIOLOGICAL PROCESS** | **cellular process (GO:0009987) 36**  cellular metabolic process (GO:0044237) 22  cellular component organization or biogenesis (GO:0071840) 7  cellular response to stimulus (GO:0051716) 6  cellular localization (GO:0051641) 5  protein folding (GO:0006457) 5  cell communication (GO:0007154) 3  signal transduction (GO:0007165) 3  transmembrane transport (GO:0055085) 3  actin filament-based process (GO:0030029) 1  maintenance of location in cell (GO:0051651) 1  vesicle-mediated transport (GO:0016192) 1  **metabolic process (GO:0008152) 26**  nitrogen compound metabolic process (GO:0006807) 24  organic substance metabolic process (GO:0071704) 24  primary metabolic process (GO:0044238) 23  cellular metabolic process (GO:0044237) 22  cellular nitrogen compound metabolic process (GO:0034641) 13  biosynthetic process (GO:0009058) 16  organonitrogen compound biosynthetic process (GO:1901566) 7  small molecule metabolic process (GO:0044281) 4  catabolic process (GO:0009056) 3  **response to stimulus (GO:0050896) 13**  response to stress (GO:0006950) 8  cellular response to stimulus (GO:0051716) 6  response to abiotic stimulus (GO:0009628) 6  response to chemical (GO:0042221) 3  response to endogenous stimulus (GO:0009719) 2  **localization (GO:0051179) 9**  cellular localization (GO:0051641) 5  macromolecule localization (GO:0033036) 5  establishment of localization (GO:0051234) 4  maintenance of location (GO:0051235) 3  **biological regulation (GO:0065007) 6**  regulation of biological process (GO:0050789) 5  regulation of biological quality (GO:0065008) 1  **homeostatic process (GO:0042592) 3**  cellular homeostasis (GO:0019725) 3  chemical homeostasis (GO:0048878) 2  **developmental process (GO:0032502) 1**  anatomical structure development (GO:0048856) 1  developmental process involved in reproduction (GO:0003006) 1  **multicellular organismal process (GO:0032501) 1**  multicellular organism development (GO:0007275) 1  post-embryonic development (GO:0009791) 1  **reproduction (GO:0000003) 1**  reproductive process (GO:0022414) 1  **reproductive process (GO:0022414) 1**  developmental process involved in reproduction (GO:0003006) 1 | **cellular process (GO:0009987) 28**  cellular metabolic process (GO:0044237) 19  protein folding (GO:0006457) 5  cellular component organization or biogenesis (GO:0071840) 4  cellular localization (GO:0051641) 4  cell cycle (GO:0007049) 3  microtubule-based process (GO:0007017) 3  cellular response to stimulus (GO:0051716) 2  transmembrane transport (GO:0055085) 2  cell communication (GO:0007154) 1  cell wall organization or biogenesis (GO:0071554) 1  maintenance of location in cell (GO:0051651) 1  signal transduction (GO:0007165) 1  vesicle-mediated transport (GO:0016192) 1  **metabolic process (GO:0008152) 20**  organic substance metabolic process (GO:0071704) 20  nitrogen compound metabolic process (GO:0006807) 19  primary metabolic process (GO:0044238) 19  cellular metabolic process (GO:0044237) 19  cellular nitrogen compound metabolic process (GO:0034641) 12  biosynthetic process (GO:0009058) 14  organonitrogen compound biosynthetic process (GO:1901566) 8  small molecule metabolic process (GO:0044281) 5  catabolic process (GO:0009056) 2  **localization (GO:0051179) 6**  cellular localization (GO:0051641) 4  establishment of localization (GO:0051234) 4  macromolecule localization (GO:0033036) 4  maintenance of location (GO:0051235) 1  **response to stimulus (GO:0050896) 4**  response to stress (GO:0006950) 3  cellular response to stimulus (GO:0051716) 2  **biological regulation (GO:0065007) 2**  regulation of biological process (GO:0050789) 2  regulation of biological quality (GO:0065008) 1  **homeostatic process (GO:0042592) 1**  cellular homeostasis (GO:0019725) 1 |
| **CELLULAR COMPONENT** | **cellular anatomical entity (GO:0110165) 43**  intracellular anatomical structure (GO:0005622) 38  cytoplasm (GO:0005737) 31  organelle (GO:0043226) 20  cytosol (GO:0005829) 10  membrane (GO:0016020) 10  cell periphery (GO:0071944) 4  envelope (GO:0031975) 3  chloroplast stroma (GO:0009570) 2  cell junction (GO:0030054) 1  endomembrane system (GO:0012505) 1  external encapsulating structure (GO:0030312) 1  extracellular region (GO:0005576) 1  extracellular space (GO:0005615) 1  extrinsic component of membrane (GO:0019898) 1  membrane-enclosed lumen (GO:0031974) 1  nuclear body (GO:0016604) 1  nucleoplasm (GO:0005654) 1  perinuclear region of cytoplasm (GO:0048471) 1  replication fork (GO:0005657) 1  side of membrane (GO:0098552) 1  **protein-containing complex (GO:0032991) 14**  intracellular protein-containing complex (GO:0140535) 2  membrane protein complex (GO:0098796) 4  ribonucleoprotein complex (GO:1990904) 4  catalytic complex (GO:1902494) 2  mitochondrial protein-containing complex (GO:0098798) 2  protein-DNA complex (GO:0032993) 2  Sm-like protein family complex (GO:0120114) 1  nuclear protein-containing complex (GO:0140513) 1 | **cellular anatomical entity (GO:0110165) 32**  intracellular anatomical structure (GO:0005622) 28  cytoplasm (GO:0005737) 26  organelle (GO:0043226) 15  cytosol (GO:0005829) 12  cell periphery (GO:0071944) 5  membrane (GO:0016020) 4  supramolecular complex (GO:0099080) 4  external encapsulating structure (GO:0030312) 3  endomembrane system (GO:0012505) 2  envelope (GO:0031975) 2  cell junction (GO:0030054) 1  extracellular region (GO:0005576) 1  membrane-enclosed lumen (GO:0031974) 1  organelle subcompartment (GO:0031984) 1  **protein-containing complex (GO:0032991) 9**  ribonucleoprotein complex (GO:1990904)5  intracellular protein-containing complex (GO:0140535) 2  membrane protein complex (GO:0098796) 2  inner mitochondrial membrane protein complex (GO:0098800) 2  proton-transporting two-sector ATPase complex (GO:0016469) 1  mitochondrial protein-containing complex (GO:0098798) 2  catalytic complex (GO:1902494) 1  ATPase complex (GO:1904949) 1 |
| **PROTEIN CLASS** | **metabolite interconversion enzyme (PC00262) 14**  oxidoreductase (PC00176) 9  transferase (PC00220) 2  hydrolase (PC00121) 2  isomerase (PC00135) 1  **translational protein (PC00263) 11**  translation factor (PC00223) 8  ribosomal protein (PC00202) 3  **protein modifying enzyme (PC00260) 7**  protease (PC00190) 4  protein phosphatase (PC00195) 2  ubiquitin-protein ligase (PC00234) 1  **transporter (PC00227) 7**  primary active transporter (PC00068) 4  secondary carrier transporter (PC00258) 3  **chaperone (PC00072) 6**  Hsp70 family chaperone (PC00027)2  chaperonin (PC00073) 2  Hsp90 family chaperone (PC00028) 1  **RNA metabolism protein (PC00031) 2**  RNA processing factor (PC00147) 2  **chromatin/chromatin-binding, or -regulatory protein (PC00077) 2**  no hit  **DNA metabolism protein (PC00009) 1**  No hit  **cytoskeletal protein (PC00085) 1**  actin or actin-binding cytoskeletal protein (PC00041) 1  **membrane traffic protein (PC00150) 1**  no hit  **protein-binding activity modulator (PC00095) 1**  G-protein (PC00020) 1  **scaffold/adaptor protein (PC00226) 2**  no hit  **storage protein (PC00210) 1**  no hit  **transfer/carrier protein (PC00219) 1**  no hit | **metabolite interconversion enzyme (PC00262) 14**  oxidoreductase (PC00176) 7  transferase (PC00220) 5  isomerase (PC00135) 1  lyase (PC00144) 1  **translational protein (PC00263) 9**  translation factor (PC00223) 5  ribosomal protein (PC00202) 4  **chaperone (PC00072) 4**  chaperonin (PC00073) 2  **cytoskeletal protein (PC00085) 4**  microtubule or microtubule-binding cytoskeletal protein (PC00157) 3  actin or actin-binding cytoskeletal protein (PC00041) 1  **transporter (PC00227) 3**  primary active transporter (PC00068) 1  secondary carrier transporter (PC00258) 1  **RNA metabolism protein (PC00031) 2**  RNA helicase (PC00032) 1  **DNA metabolism protein (PC00009) 1**  No hit  **membrane traffic protein (PC00150) 1**  no hit  **protein modifying enzyme (PC00260) 1**  ubiquitin-protein ligase (PC00234) 1  **scaffold/adaptor protein (PC00226) 1**  no hit |
