# Supplementary material for: Proteomics- and metabolomics-based analysis of the regulation of germination in Norway maple and sycamore embryonic axes
Source: Tree Physiol. 2025 Jan 6;45(2):tpaf003. doi: 10.1093/treephys/tpaf003 (PMC11791354; doi:10.1093/treephys/tpaf003)

Figure S2. Volcano plots showing significantly upregulated (blue) and significantly downregulated (red) metabolites in imbibed (A) and germinated (B) Norway maple seeds as compared to the sycamore seeds; significantly upregulated (red) and significantly downregulated (blue) proteins in imbibed (C) and germinated (D) Norway maple seeds as compared to sycamore seeds; significantly upregulated (red) and significantly downregulated (blue) peptides with MetO sites at imbibed (E) and germinated (F) Norway maple seeds as compared to the sycamore seeds. For each protein and metabolite, the fold change (in $\log_2$) is indicated on the X-axis while the statistical significance (in $-\log_{10}$ p-value) is indicated on the Y-axis (FDR < 0.05 and $|\log_2 FC| = 2$). Individual metabolites are listed in Table S2. Individual proteins are listed in Tables S4, S6, S8 and S10. APL – Norway maple, APS – sycamore, I – imbibed, G – germinated.

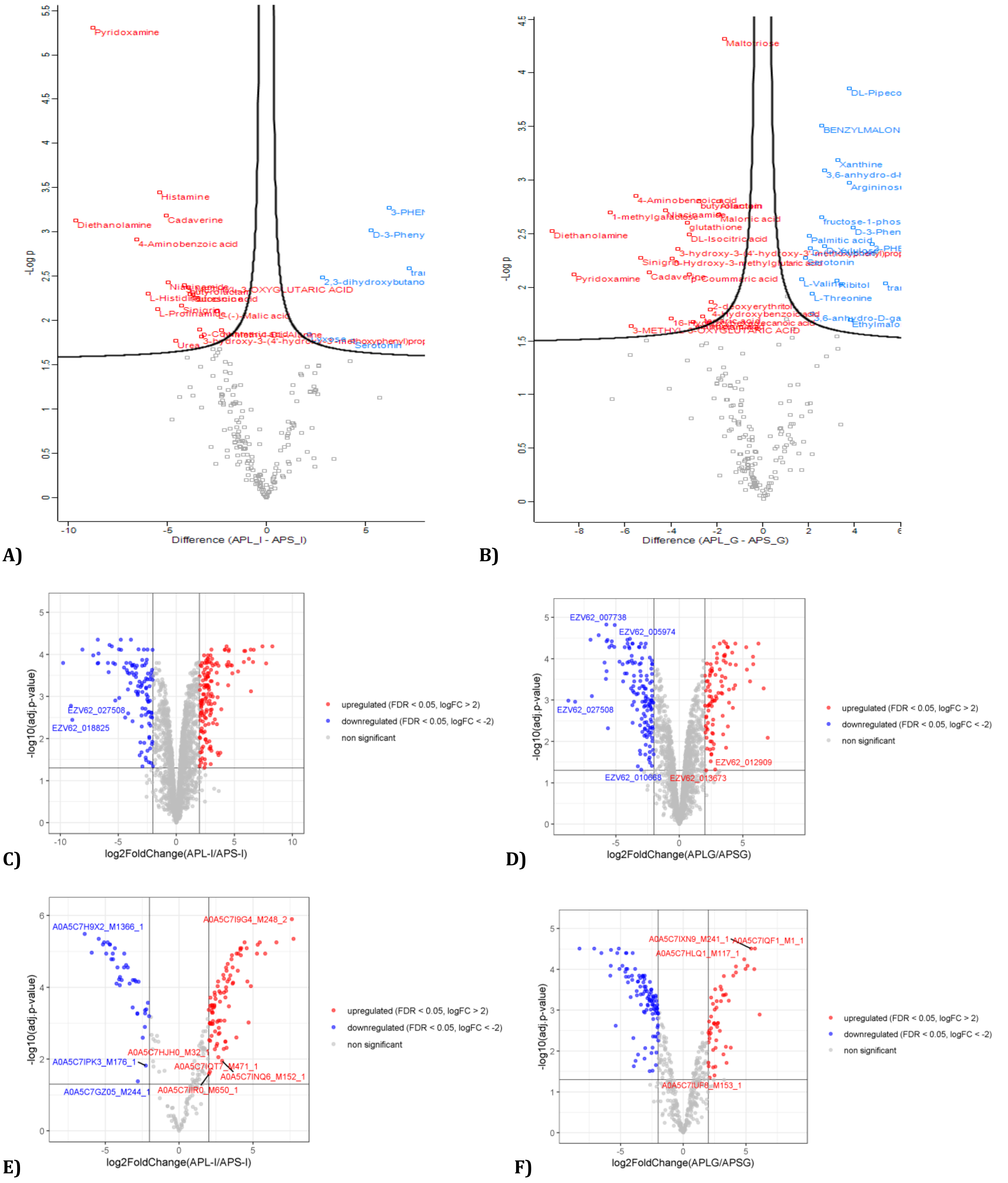

Supplement: Figure_S2_tpaf003 [file figure_s2_tpaf003.pdf]
